# Cholesterol Hot Spot Automated Mapping Protocol (CHAMP): Identifying Cholesterol-Binding Hot Spots in Membrane Proteins

**DOI:** 10.1021/acs.jcim.6c01419

**Published:** 2026-09-23

**Authors:** David Sotillo-Núñez, Matteo Nardi Cesarini, Gian Marco Elisi, Mattia Bernetti, Giovanni Bottegoni

**Affiliations:** † Department of Biomolecular Sciences, 19044 Università degli Studi di Urbino Carlo Bo, Via Ca’ Le Suore, 2/4, Urbino 61029, Italy; ‡ School of Pharmacy, University of Birmingham, Edgbaston, Birmingham B15 2TT, U.K.

## Abstract

Biological membranes are dynamic and heterogeneous environments that actively influence protein structure and function. Cholesterol, in particular, has been widely reported to modulate membrane proteins through binding to specific interaction sites. However, the identification of these sites remains challenging due to the intrinsic complexity and dynamics of lipid bilayers, especially when multiple systems and membrane environments are considered. Here, we present CHAMP (Cholesterol hot spot automated mapping protocol), an automated Python-based workflow that combines coarse-grained molecular dynamics simulations with a dual analysis based on contact persistence and spatial density to identify cholesterol-binding hot spots at protein–membrane interfaces. The protocol requires limited user intervention and is designed to enable consistent and reproducible analyses across different targets. We validated CHAMP on the serotonin transporter and the cannabinoid receptor 1 (CB_1_), where it recovers known cholesterol-binding regions reported in experimental and computational studies. In the CB_1_ system, the method also identifies a potential additional interaction site. To assess the generality of the approach, we applied the protocol to the CB_1_ negative allosteric modulator Org27569, showing that it can capture relevant interaction hot spots beyond sterol molecules. Overall, CHAMP provides a practical framework for mapping interaction hot spots at protein–membrane interfaces and may support large-scale and comparative studies of membrane proteins. Limitations of the current implementation and possible extensions are discussed.

## Graphical abstract

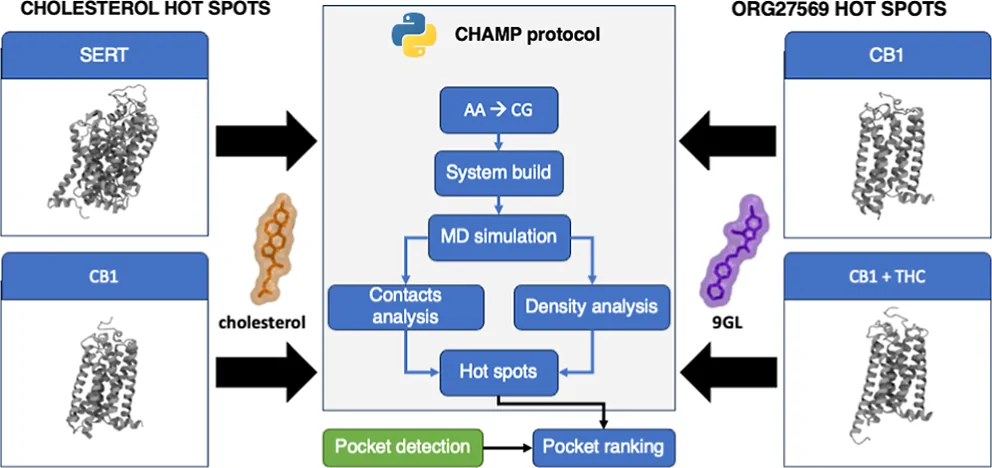

## Introduction

Biological membranes are not inert solvents for membrane proteins but chemically diverse, dynamically structured environments. Hundreds of lipid species, asymmetrically distributed across the bilayer, collectively generate a heterogeneous landscape in which lipid–protein interactions actively shape protein stability, conformational equilibria, and cellular trafficking. In this context, lipids act as molecular regulators rather than passive scaffolds.^1^ Lipid–protein interactions can be broadly classified as nonspecific, where membrane components modulate protein behavior through the bulk physical properties of the bilayer, or specific, involving discrete binding sites at which lipids act in a manner analogous to allosteric ligands. Identifying such lipid-binding hot spots is therefore a powerful strategy for uncovering regulatory sites at the membrane–protein interface, with direct relevance for drug discovery, as these sites represent potential targets for the design of novel allosteric modulators.^2^ However, the dynamic and heterogeneous nature of biological membranes makes the systematic identification and characterization of these interactions challenging, particularly when extended across multiple targets and membrane environments.

Among membrane lipids, cholesterol has emerged as a paradigmatic example of a specific allosteric modulator across diverse classes of membrane proteins.^2^ Numerous experimental and computational studies have demonstrated that cholesterol can bind to defined regions of membrane proteins and modulate their stability, conformational equilibria, and signaling behavior.^2^ Representative examples include the tyrosine kinase receptor TrkB, where cholesterol binding to a specific motif in the transmembrane region facilitates brain-derived neurotrophic factor signaling,^3^ and the T-cell receptor–CD3 complex, in which cholesterol association at the transmembrane interface inhibits complex activity.^4^ In G protein-coupled receptors (GPCRs), cholesterol has been shown to directly influence ligand-binding properties and functional activity,^5,6^ acting as either a positive or negative allosteric modulator (NAM) depending on the receptor and context,^7–9^ and in some cases modulating receptor oligomerization.^10^ GPCRs, like many other membrane proteins, populate multiple conformations that underpin their pleiotropic signaling behavior.^11,12^ This intrinsic conformational plasticity makes allosteric modulation a particularly attractive therapeutic strategy for fine-tuning receptor activation and downstream signaling.^13^ At the same time, insights derived from individual receptors may not always generalize across a protein family. Nevertheless, comparative analyses across multiple GPCR families are especially valuable, as they can leverage the conserved structural fold and shared activation mechanisms to identify common patterns of lipid interaction that may not be apparent when studying receptors in isolation.

Molecular dynamics (MD) simulations have played a central role in characterizing lipid–protein interactions and in identifying potential allosteric hot spots for cholesterol binding.^14^ Given the size and complexity of membrane–protein systems, coarse-grained (CG) MD simulations have proven especially effective, enabling the exploration of lipid organization and protein interactions over extended time scales and at manageable computational cost. The Martini force field for CG-MD, in particular, has been widely applied to study cholesterol interactions with GPCRs and other membrane proteins.^14–16^ In 2017, Genheden and colleagues used the Martini force field to study how cholesterol interacts with the β2-adrenergic receptor (β_2_AR) and the A_2_A adenosine receptor by embedding these receptors in 1-palmitoyl-2-oleoyl-sn-glycero-3-phosphocholine (POPC)/cholesterol (30%) membranes.^17^ They, then, simulated these systems for an extended 50 μs production run. The authors mapped density distribution of cholesterol polar heads around the receptors and tracked the cholesterol occupancy by using a 5 Å cutoff distance to determine whether a polar head bead was contacting the protein. Their analyses produced several clusters of residues with high cholesterol occupancy for both receptors, with the highest occupied cluster reaching an occupancy of more than 70% in both cases. The cluster with the highest occupancy in the β_2_AR (72%) was located at the TM1-7 region, while in the adenosine A_2_AR (74%) it was located at the TM4-5 area. Their CG-MD simulations suggested that regular atomistic MD usually cannot identify certain interaction areas because of being unable to reach full equilibration. In the same year, Rouviere and colleagues used Martini MD simulations in combination with atomistic simulations.^18^ They used a high-resolution structure of the adenosine A_2_AR to build a system where the protein was embedded in a POPC/cholesterol (30%) membrane. The system was simulated for an extended period of 200 μs to obtain an increased sampling of cholesterol distribution around the protein, allowing us to predict preferred areas of interaction. While volumetric mapping of cholesterol density is effective for identifying preferentially occupied regions when stringent cutoffs are applied, it is not well suited for predicting true binding sites. To address this limitation, the authors introduced a scoring scheme that rewards persistent interaction events while discarding transient contacts shorter than 1 μs. Scores are accumulated for residues involved in long-lived interactions that are repeatedly revisited over time. Mapping the highest-scoring residues onto the protein surface revealed several putative cholesterol-binding sites, the most prominent of which is located at the TM5–TM6 interface. This site coincides with the crystallographic cholesterol position observed in structures bound to the ZM241385 inverse agonist and was subsequently validated through atomistic simulations. In 2019, Hedger and colleagues investigated cholesterol interactions with the class F GPCR Smoothened.^19^ To achieve extended sampling and enable the simulation of large systems, they employed CG-MD simulations of Smoothened embedded in POPC membranes containing 20% cholesterol. Eight independent systems were constructed and simulated for 10 μs each. Analysis of these trajectories revealed specific cholesterol interaction regions located at the extracellular tips of the TM2–TM3 helices. To further assess the robustness of these interactions, the simulations were repeated using a five-component membrane consisting of cholesterol and of four types of phospholipids. The absence of significant differences between the two membrane compositions supported a genuine affinity of cholesterol for this region, which was subsequently confirmed by atomistic simulations. In the same year, Song and colleagues examined lipid interactions of the A_2_AR, including cholesterol.^20^ To assess how receptor activation influences lipid binding, they performed CG-MD simulations on three receptor conformations: inactive, active, and active bound to a mini-G_s_ protein. Each structure was embedded in a complex, asymmetric membrane containing ten lipid species, including cholesterol. For each activation state, ten independent systems were constructed with randomized initial lipid distributions, yielding a total simulation time of 8 μs. Lipid density maps and residue-level residence times were then computed, revealing seven lipid-binding sites with varying degrees of activation-state dependence. Binding sites located at the TM1–TM2–TM4 and TM3–ECL2–TM4 interfaces exhibited increased persistence in both active conformations. This behavior was attributed to conformational shifts of TM4 upon activation and to tight packing of PIP2 around these regions, which restricts cholesterol dissociation. Notably, the authors also reported evidence of competition between cholesterol and PIP2 at the TM5–TM6 site.

Despite these advances, large-scale computational studies spanning multiple GPCR families remain relatively scarce. While insightful, existing efforts have often focused on limited subsets of receptors^21^ or have required substantial manual effort in system preparation, simulation management, and data curation.^22^ Moreover, many studies have not been explicitly designed to systematically interrogate the protein–membrane interface, leaving GPCR–cholesterol interactions underexplored from a comparative, family-wide perspective. These challenges are compounded by the fact that, despite extensive evidence for cholesterol binding, consensus sequence motifs such as CRAC and CARC identified for other protein families do not consistently capture cholesterol interaction sites across GPCRs,^23–25^ highlighting the need for structure- and dynamics-based approaches.

To facilitate large-scale and systematic analyses of GPCRs, as well as other membrane protein families, existing workflows for identifying cholesterol interaction hot spots require adaptation into more general and automated frameworks. Notable examples of software addressing lipid–protein interaction analysis include ProLint^26,27^ and PyLipID,^28^ which enable the systematic analysis of both atomistic and CG MD trajectories to identify potential lipid-binding hot spots. Although these tools provide standardized and automated analysis of simulation trajectories, the user is still required to take care of simulation setup and execution, possibly combining multiple software packages and manually connecting the output of one stage to the input of the next. In this context, Ferraro and colleagues introduced a protocol to identify cholesterol interaction sites at the protein–membrane interface of the serotonin transporter (SERT).^16^ The core idea of this approach is to detect cholesterol-binding hot spots directly from CG MD trajectories by combining statistical analysis of protein–cholesterol contact durations with the identification of reproducible cholesterol density regions around the protein. Specifically, statistically significant outliers corresponding to unusually long-lived protein–cholesterol contacts are matched to persistent spatial density maxima, enabling the robust identification of putative cholesterol binding sites.

Building on this previous study, we sought to extend and generalize this strategy into a more robust, automated, and scalable framework. While Ferraro’s protocol^16^ proved highly effective for the system under investigation, its application to larger sets of proteins would benefit from increased automation, standardized setup procedures, and streamlined analysis. Here, we present CHAMP (Cholesterol Hot spot Automated Mapping Protocol), a fully automated protocol that identifies cholesterol hot spots using only the protein structure as input, enabling consistent application across multiple targets and membrane environments. We also show how, although developed with cholesterol in mind, the framework can be readily extended to other allosteric modulators acting at the membrane–protein interface, providing a general computational strategy for the investigation of protein–membrane interaction-mediated allostery.

## Methods

### Input Structures

The structures of SERT and of the sodium–potassium ATPase pump were cocrystallized with one cholesterol (PDB ID 7LIA,^29^ and 3A3Y,^30^ respectively), while the structure of the cannabinoid 1 (CB_1_) receptor was cocrystallized with two cholesterol molecules (PDB ID 7FEE).^31^ Each crystal structure was processed according to the protocol described in the following sections. As initial step, the preoriented protein structure was retrieved from the orientations of proteins in membranes (OPM) database^32^ and any nonprotein atom, such as ligands and other small molecules, removed and missing heavy atoms and explicit hydrogens added using PDBFixer.^33^

### CG Conversion and System Build

To enable extensive contact sampling, the protocol relied on the Martini 3 CG representation. The atomistic input structure was converted to its CG form using martinize2, in the version of the script distributed within the Vermouth package.^34^ Default conversion settings were applied whenever possible to maintain robustness and minimize failures due to PDB formatting inconsistencies. However, several parameters were customized and explicitly defined to ensure consistent and reliable output. The *-p* backbone option created a section in the topology that allowed positional restraints to be applied to backbone beads during CG simulations. Elastic network terms were introduced with -*elastic*, which generated elastic bonds (700 kJ mol^–1^ nm^–2^) between backbone beads within a 9 Å cutoff and separated by at least three residues. This configuration stabilized the overall tertiary structure without restricting side-chain motion. Initial residue numbering was retained to simplify downstream analysis and hydrogens were ignored (*-ignh*) to prevent atom name conflicts. The *-merge* all flag forced martinize2 to write a single topology, avoiding unwanted fragmentation around unresolved regions. As a final safety step, the *-eunit* system option ensured that the elastic network was built for the complete protein before merging, preventing inconsistencies when gaps were present. A Martini 3 CG PDB file corresponding to the original structure together with its topology were generated.

After CG conversion, the protein–membrane system was assembled using the insane.py utility,^35^ a grid-based tool that allows generating a Martini 3 membrane of defined composition. As a result, a cholesterol-containing bilayer is built around the CG protein obtained in the previous step. In this system, the protein is placed at the center of a 14.5 × 14.5 × 10.5 nm orthorhombic box. The procedure consists in constructing the membrane around the protein and filling the simulation box with water beads and ions to neutralize the total charge. After the conversion, the CG representations employed 1334 (SERT) and 705 (CB_1_) beads, respectively. Embedding the protein in a 75:25 POPC/cholesterol membrane and adding counterions, resulted in the following protein–membrane systems: (i) SERT: 5832 POPC, 1449 cholesterol, 10280 water, 4 chlorine beads, for a total of 18899 beads including the protein; (ii) CB_1_: 6096 POPC, 1521 cholesterol, 10388 water beads, 9 chlorine beads, for a total of 18719 beads including the protein. For SERT, an additional system was assembled using a DSPC(50%):POPE (25%):CHOL(25%) membrane composition. This ternary membrane system encompassed: 3888 DSPC, 1932 POPE, 1449 cholesterol, 10287 water and 4 chlorine beads, for a total of 18894 beads including the protein.

In addition, another system corresponding to a sodium–potassium pump, embedded in a POPC(75%)/CHOL(25%) membrane, was also processed. The resulting beads for that system consisted of: 1100 protein, 5844 POPC, 1449 cholesterol, 10568 water and 4 sodium, for a total of 18965 beads. Results for this additional system have been reported in Supporting Information S3.

Because lipids were placed randomly during system assembly, the generated replicas of each system showed slight variations in the absolute numbers of nonprotein components, while the component ratios remained strictly constant.

### Equilibration and Molecular Dynamics Simulations

Because grid-based membrane construction can introduce unfavorable contacts or rigid conformations, the system underwent a standardized equilibration protocol before production simulations. According to best practices, from this point onward, each step of the protocol was performed N times, with N defined by the number of replicas specified in the run settings. The MD engine adopted for all simulations was GROMACS 2021.5.^36,37^ System charges are handled by the Martini 3 topology, which was assembled during the system building process. The reaction-field model was used for short-ranged electrostatics, no long-range PME was used. The v-rescale thermostat^38^ was used through all equilibration steps and in the production run, while the barostat switched from Berendsen^39^ to Parrinello–Rahman^40^ at production. Solute (protein and membrane) and solvent (water and ions) were kept into separate temperature coupling groups. Equilibration began with 5000 steps of steepest-descent energy minimization, followed by a 20 ps NVT simulation to stabilize the temperature at 300 K. Three consecutive NPT simulations were then performed. In the first, backbone restraints were applied (1000 kJ mol^–1^ nm^–2^) while the membrane relaxed during a 100 ns run. In the second, restraints were removed, and the system was equilibrated for 1 ns using a 1 fs time step to limit abrupt motions of newly unrestrained atoms. The final NPT step consisted of a 1 ns simulation using the recommended 20 fs Martini time step. After equilibration and before production, membrane quality was evaluated. Membrane thickness, area per lipid, and lipid order parameters were calculated with MDAnalysis^41,42^ and LiPyphilic,^43^ providing quantitative descriptors of bilayer structure and organization. Partial-density profiles along the *Z*-axis were generated to assess leaflet continuity. Production simulations were then carried out for 30 μs to provide sufficient sampling across systems and replicas.

### Contacts Analysis

Cholesterol–protein contacts were quantified to identify long-lived interaction events along the simulations. Contact detection was performed with MDAnalysis by iterating over all frames of the trajectory and identifying residues within 6 Å of any cholesterol bead. This cutoff was selected based on previously published work.^16^ Contacts were evaluated only after the first 500 ns of the production simulation to ensure that measurements were taken within the fully equilibrated regime. For each residue, consecutive frames in which cholesterol remained within the cutoff were grouped into continuous contact events. This approach was adopted to distinguish sustained interactions from intermittent binding and unbinding. Although two residues may exhibit the same total number of contact frames, their biological relevance differs if one forms a single long-lived event while the other engages in multiple short, transient encounters. For this reason, the analysis relied on continuous-contact durations rather than simple frame occupancy. Only residues exhibiting at least one contact were considered for further analysis to avoid inflating the data set with residues that could not physically interact with the membrane (e.g., residues facing the extracellular, intracellular, or internal cavity regions). From the resulting list of events, the longest contact per residue (*t*_max_) was extracted. To identify statistically significant long-lived contacts, the interquartile range method was applied using eq 1

1 T=1.5×IQR+Q3

where *T* defines the upper threshold for outlier detection. This approach flagged residues with contact durations that substantially exceeded the central distribution. The output of this step consisted of a comma-separated table listing the residues classified as statistical outliers in each replica. The use of the IQR-based criterion provides a robust, distribution-independent approach for identifying extreme events without assuming an underlying statistical model.

### Density Analysis

The spatial density of cholesterol around the receptor was quantified by computing the Spatial Density Function (SDF), which describes the probability of cholesterol occupying different regions of the simulation box. The 3D space was discretized into voxels, each assigned a value proportional to the frequency with which cholesterol beads occupied that region over the course of the trajectory. Retaining only the voxels with the highest values enabled identification of the spatial regions most frequently sampled by cholesterol. Prior to SDF calculation, the trajectory underwent two preprocessing steps using the GROMACS trjconv tool: the first corrected for periodic boundary conditions and centered the protein in every frame; the second removed protein rotation and translation, fixing the receptor at the center of the box while allowing the remaining components of the system to diffuse freely. The SDF was then computed with the GROMACS spatial tool, which produced a Gaussian cube file containing the voxel-based volumetric data. Voxel coordinates were converted from Bohr to Ångström units, and top 0.01% of voxels, which correspond to the regions with the highest occupation probability, were extracted, converted to Cartesian coordinates, and saved as dummy atoms in an .xyz file.

### Replica Merging

The results of the contact and density analyses across multiple replicas were merged to generate a final file representing the system’s hot spots. For contact-based outliers, the *t*_max_ values from all replicas were compared, and the union of outlier residues was stored. Duplicate residues were common, as the most relevant contacts typically appeared as outliers in multiple replicas; in these cases, the longest contact observed across replicas was retained. For the density analysis, the voxel coordinates from all replicas were combined through a union merge, removing duplicate points.

### Output

For each system, four output files were generated. The merged contact outliers were written as a CSV file containing the residue ID, residue name, and *t*_max_. Because one of the aims of the pipeline was to enable large-scale GPCR analyses, an optional column with GPCRdb^44,55^ generic residue numbers (class A) can be added for comparative studies. The cholesterol spatial distribution was stored as a set of dummy atoms in an .xyz file, preserving the element label and Cartesian coordinates for direct visualization. A PDB file of the starting structure, aligned to the last frame of the trajectory used for SDF calculation, was also generated to facilitate inspection of density regions relative to the receptor. Finally, a reduced PDB file containing only the outlier residues and the reproducible density points within 5 Å of those residues was produced for straightforward visualization. This file was generated from the aligned structure so that it could be directly visualized alongside the density cloud.

### Pocket Scoring

A scoring procedure was developed to rank membrane-interface pockets according to their predicted propensity to accommodate cholesterol, using the information generated during hot spot detection. Pockets were defined as sets of residues identified by a pocket-detection algorithm. Each pocket was evaluated based on (i) the number of residues classified as outliers in the contact analysis and (ii) the number of SDF density points located near the pocket. Density points associated with a given pocket were defined as all density points lying within 5 Å of any atom of any residue forming that pocket. Scoring weights were assigned as 1000 for each outlier residue and 1 for each associated density point. The score was applied to all detected pockets, which were then ranked accordingly. Although the procedure is in principle compatible with the output of any pocket-detection protocol, the examples shown in this study were based on NanoShaper, a probe-based method for cavity detection.^45^ NanoShaper was run with a small probe of 1.4 Å and a large probe of 6.5 Å, together with a minimum water count of 2. The size of the larger probe was required to appropriately capture the shallow and planar cavities commonly found at membrane–protein interfaces. Pockets detected by NanoShaper were filtered to retain only membrane-interface sites compatible with cholesterol binding. Internal or extra-/intracellular pockets were removed by discarding any cavity whose centroid laid within 5 Å of the protein’s vertical axis or outside the transmembrane region defined by the OPM pseudolayers. The remaining pockets were then evaluated using the scoring procedure described above.

Results were compared for dynamic pocket detection and ranking on the SERT system through the Pocketron utility,^46^ as shown in the Supporting Information S1.^46^ To ensure the protocol is not biased by a specific starting crystal structure, an alternative SERT structure (PDB ID: 5I6X)^47^ with a different cholesterol binding site was tested. Analysis of pocket detection and scoring is provided through the application of NanoShaper and reported in Supporting Information S2.

### Parameterization of the Ligands

Org27569 topology was generated through manual atom-to-bead mapping and definition of bonded terms, as extensively illustrated in Supporting Information S6. Briefly, the distribution of Org27569 CG bonded terms was compared with full-atom MD distributions, observed in standard MD and Hamiltonian replica exchange MD.^48^ Twelve beads were used to represent the allosteric ligand, using the mapping depicted in Figure S7. Conversely, the tetrahydrocannabinol (THC) topology, consisting of 8 beads, was retrieved from a previous work by Bartocci and colleagues.^49^

### CB_1_ Receptor-Org27569 System

For this system, we started from the crystal structure of the complex between the CB_1_ receptor and the Org27569 allosteric ligand (PDB ID: 6KQI).^50^ The ligand was removed and, following the standard procedure in our protocol, the CB_1_ receptor was converted to its CG representation and inserted into the bilayer. We then adapted the insane.py stage of our protocol to replace cholesterol insertion with the ligand Org27569. The ligand in its crystallographic conformation was aligned along the *z*-axis and then inserted into the membrane, uniformly distributing ligand copies across both leaflets. In this case, the membrane displayed a 95:5 POPC/ligand ratio, as previously reported.^49^ This resulted in 17 copies of Org27569 per membrane leaflet, with a total of 34 ligand copies distributed across the bilayer. The CG representation encompassed 686 beads for the protein, 7752 for POPC, 408 for Org27569, 10625 for water, 11 for chlorine, for a total of 19482 beads including ligands for replica 1.

### CB_1_ Receptor-Org27569-THC System

To construct the system including the THC ligand in the orthosteric binding site, we started from the crystal complex between the THC synthetic analog HU-210 and the CB_1_ receptor (PDB ID: 9ERX).^51^ With minor modifications to the HU-210 structure, we converted this ligand into THC and the resulting complex was minimized with MacroModel,^52,53^ restraining the position of protein atoms. We then used the same procedure described in the previous section to insert the protein–ligand complex in a 95:5 POPC/Org27569 ratio membrane. The CG representation encompassed 686 beads for the protein, 7692 for POPC, 384 for Org27569, 8 for THC, 10585 for water, 13 for chlorine, for a total of 19397 beads including ligands for replica 1.

### Org27569-Containing Systems’ Production Runs

Our standard procedure was employed for equilibrating both systems. In the production stage, 10 runs lasting 10 μs each were performed for the system with Org27569 in the membrane, and 5 runs of 10 μs for the system with Org27569 in the membrane and THC in the orthosteric site.

## Results and Discussion

### Simulations and Analysis Pipeline

An automated protocol was developed to identify binding hot spots of cholesterol and, with minimal adaptation, other membrane components at the protein–membrane interface, requiring minimal user input beyond specifying run parameters. The protocol is summarized in Figure 1 and described in detail in the following sections.

**Figure 1:**
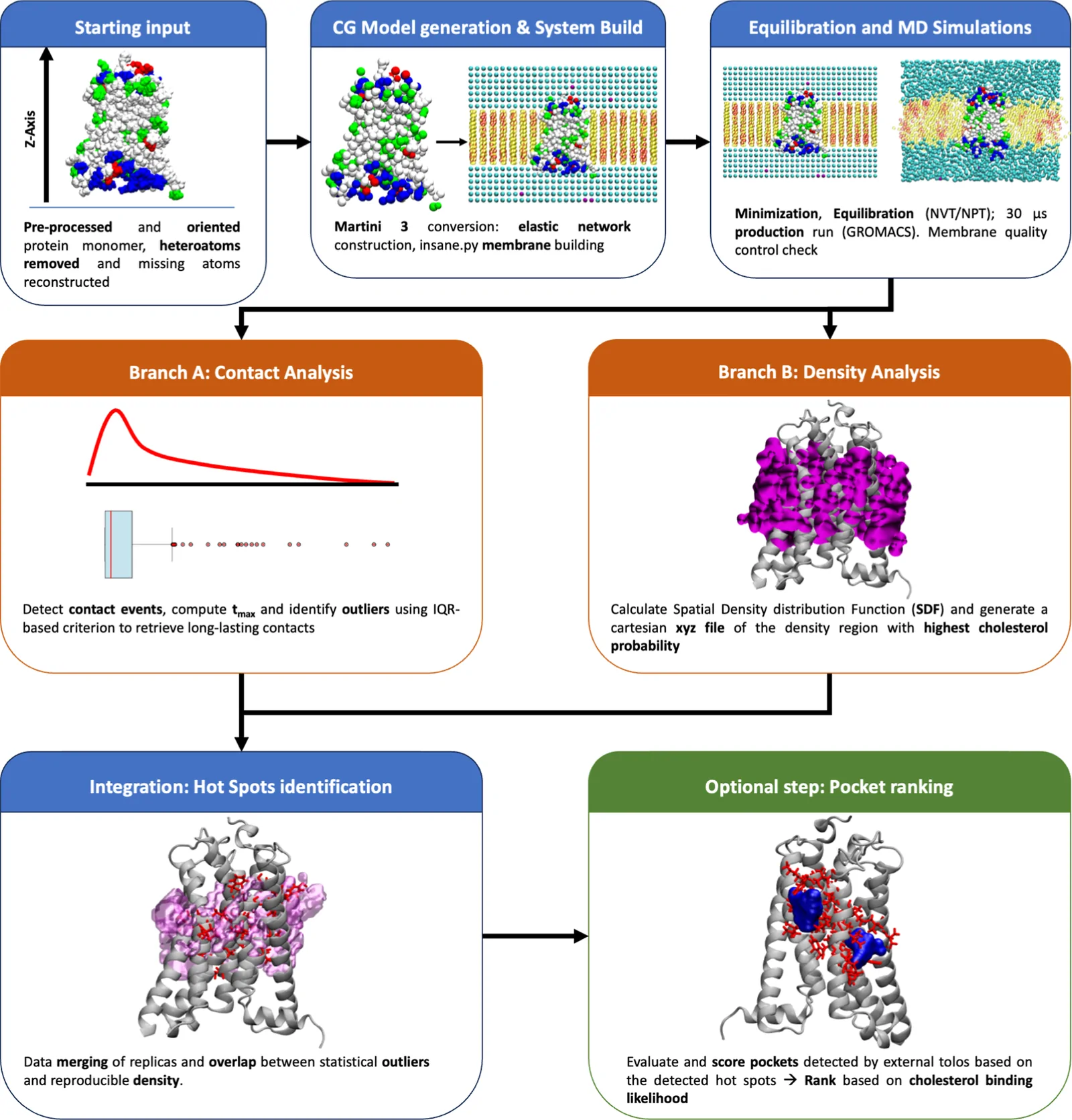
Overview of the cholesterol hot spot automated mapping protocol (CHAMP). The workflow begins with a preoriented membrane protein, which is converted to the Martini 3 CG representation and embedded in a lipid bilayer of the desired composition. Following system equilibration and MD production runs, trajectories are processed through two approaches: a contact-based analysis (A) and a density distribution computation (B). The results are integrated to identify agreement regions, labeled as hot spots. Optionally, the protocol can incorporate a user-supplied file with predefined pockets, allowing for pocket scoring and ranking based on their predicted cholesterol binding propensity.

### Starting Input

The protocol requires a preprocessed, membrane-oriented PDB structure. Structures obtained from the OPM database^32^ are directly compatible, as they are already positioned within the membrane (typically aligned along the *Z*-axis) and include dummy atoms marking the upper and lower bilayer boundaries. All ligands and nonprotein components must be removed and missing atoms restored. The final input consists of a single oriented monomer with complete receptor residues and no heteroatoms.

### CG Model Generation

The CG conversion procedure yields a single, consistent Martini 3 representation for each receptor. Fragmentation issues near unresolved regions, including ICL3 in many GPCR crystal structures, are pre-emptively addressed, residue numbering is preserved, and each output includes a complete elastic network and unified topology. Application of insane.py on these standardized models resulted in a complete protein–membrane system with uniform box dimensions, the intended membrane composition, and neutralizing solvent. Each system had the protein correctly centered and embedded in the bilayer, providing consistent inputs for downstream equilibration and sampling stages.

### Equilibration and MD Simulations

The equilibration stage generates fully prepared membrane–protein systems ready for CG-MD simulations. After completing minimization, NVT, and NPT steps, the pipeline produces quality-control outputs, including membrane structural descriptors and density profiles along the *Z*-axis. These files allow users to verify that the system has been assembled and processed correctly before production sampling. At the end of this stage, the pipeline provides a standardized, simulation-ready input for the 30 μs production run performed with the adopted MD engine, GROMACS.

### Contact Analysis

The contact-analysis stage yields a curated data set that captures uninterrupted cholesterol–protein interactions. Grouping frames into continuous contact events enables the pipeline to differentiate biologically meaningful, long-lived cholesterol interactions from rapidly exchanging contacts, even when residues exhibit identical total contact occupancies. After filtering noninteracting residues and computing *t*_max_ values, the IQR-based criterion is applied, and residues classified as long-lived contact outliers for each replica can be identified. This standardized output provides a consistent summary of cholesterol-binding events for downstream comparison.

### Density Analysis

The density-analysis stage generates a volumetric map describing the regions that are most frequently occupied by cholesterol around the receptor. After trajectory preprocessing and SDF calculation, a Gaussian cube file and a corresponding .xyz file are produced, containing the Cartesian coordinates of the top-density voxels. These points represent the highest-probability cholesterol locations. All frames of the MD trajectories were retained for analysis, since trajectory striding can influence the resolution of cholesterol density maps (see Supporting Information S5).

The overlap of persistent cholesterol-residue contacts and high-density regions across all replicas defines the actual interaction hot spots, highlighting residues and spatial regions that consistently show the most relevant cholesterol binding.

### Output

The pipeline outputs a unified set of cholesterol-interaction features for each system. Contact outliers from all replicas are consolidated into a single list, while density clouds from individual replicas are combined into a consensus spatial distribution. Together, these outputs define reproducible cholesterol-binding hot spots, enabling direct visual and quantitative assessment of the most relevant interaction regions on the receptor.

### Pocket Rank

The final, optional step of the pipeline uses the information derived from hot spot detection to evaluate membrane-interface pockets, whether obtained from any pocket-prediction tool or defined directly by the user, for their compatibility with cholesterol binding. By combining outlier residues from the contact analysis with high-probability regions from the SDF, pockets supported by both types of evidence are assigned greater relevance, thus separated from pockets that lack predicted affinity for cholesterol. The chosen weighting scheme reflects the relative importance of persistent contacts over diffuse density contributions, ensuring that pockets supported by statistically significant long-lived interactions are prioritized. Alternative weighting schemes could be explored in future applications depending on the specific system under investigation.

### Python Package

To integrate all the stages into a unified protocol and allow straightforward usage by the community, we developed a dedicated Python package named CHAMP (Cholesterol Hot spot Automated Mapping Protocol). The package includes all required external tools and modules, except for GROMACS, which must be compiled separately. To ensure flexibility and scalability, the workflow exposes a wide set of configurable parameters rather than relying on hardcoded values. While the great advantage of our protocol is the ability to provide results automatically by running the full pipeline starting from a PDB, CHAMP can also be used in a modular fashion, allowing users to repeat or customize specific steps if necessary or to perform only the analysis stages on independently generated trajectories. A full tutorial is provided in the dedicated GitHub repository (https://github.com/Urbino-CAMD-Lab/CHAMP) and is reported as Supporting Information File 2. Key options include specifying membrane composition, selecting the number of replicas, or providing a predefined pocket list to directly obtain their scoring and ranking at the end of a run. When using CHAMP on a GPCR, users can also supply a GPCRdb-annotated file to maintain Ballesteros-Weinstein numbering in the output.^54^ The package also includes a convenient toolbox to assist users with input preparation. Briefly, one script generates a template parameter file needed to launch the protocol. Another can simply align a structure along the *Z*-axis when no oriented model is available, although this should be used with care, as some proteins have complex orientation and tilt. A third tool is a cleanup utility that can automatically remove unnecessary intermediate files or perform a more extensive cleanup, preserving the files required to reproduce and inspect the final results. Additionally, we provide an automatic GROMACS benchmarking routine to help users determine the optimal configuration for Martini 3 CG simulations.

Note that in the Martini 3 cholesterol topology, a virtual site is used to reproduce the sterol ring geometry. Currently, while most tasks, such as short-range nonbonded interactions, PME, and bonded forces, can still be off-loaded to the GPU, the presence of virtual sites prevents the MD update step (position and velocity integration and virtual-site reconstruction) from running on the GPU.^37^ This forces the integrator to run on the CPU, making overall performance sensitive to CPU–GPU communication and strongly dependent on the number of CPU cores available. Given only the number of available cores, the provided script selects suitable test configurations, runs the benchmarks, and produces a concise performance summary.

A final point concerns the size of the data generated. For a standard run (approximately 11.5k beads in a 14.5 × 14.5 × 10.5 nm box) consisting of two 30 μs replicas, the total output can reach up to 800 GB. However, except for debugging purposes, most users will only retain around 500 GB, corresponding to raw and centered trajectories, protein-cholesterol contacts output, density files, and the hot spots structure file. The remaining space is occupied by intermediate files, and other outputs. Importantly, the hot spot identification itself relies on highly reduced representations of the data (contact summaries and filtered density points), meaning that intermediate files can be safely discarded after analysis without affecting the final results. In this respect, we also assessed the impact of trajectory striding to optimize data handling requirements without compromising the accuracy of the density maps; as detailed in Supplementary S5, a saving stride of 200 ps, i.e. every 10th frame saved relative to the original trajectory, reduces the final trajectory size while maintaining a consistent pocket ranking for CB_1_ receptor.

### Serotonin Transporter

We first applied CHAMP to SERT to enable direct comparison with the results reported by Ferraro et al.^16^ The transporter was analyzed in its outward-facing conformation, taken from a cryo-EM structure of human SERT bound to serotonin, which also features a cholesterol molecule resolved at the membrane-exposed surface (PDB ID: 7LIA,^29^). To assess the effect that a realistic membrane could have in comparison with a simpler composition, we applied the protocol twice, each time using a different membrane. The first system used a simple POPC(75%)/CHOL(25%) bilayer. Although modern simulations increasingly favor complex lipid mixtures, POPC/CHOL membranes remain a robust and widely used benchmark, capable of reproducing liquid-ordered and liquid-disordered behavior depending on sterol content.^55^ This composition provides a generalizable reference point that facilitates comparison across systems. The second membrane reproduced the lipid environment used by Ferraro et al., a ternary DSPC (50%)/POPE (25%)/CHOL(25%) mixture designed to better approximate raft-like membrane domains.^56^ From each run, two independent replicas of 30 μs were generated.

Interestingly, membrane composition had only a modest influence on the residues classified as statistically significant long-lived contacts (Table 1), with *t*_max_ values of the outliers ranging from 73.72 ns (POPC/CHOL) and 82.66 ns (DSPC/POPE/CHOL) to 422.46 ns (POPC/CHOL) and 471.46 ns (DSPC/POPE/CHOL). The POPC/CHOL system yielded 39 outliers, whereas 36 were detected in the DSPC/POPE/CHOL system, with substantial agreement: 27 residues were shared between the two distributions. This level of agreement indicates that, in this case, the protocol consistently recovers the principal features of cholesterol recognition, even when applied across different membrane environments. Both sets also showed notable convergence with the 16 outliers reported by Ferraro et al. Six residues appeared in all three data sets, three of which belong to the cholesterol binding pocket resolved in the experimental structure, considering those residues within a shell of 5 Å of the cholesterol molecule (Figure 2).

**Table 1:** Comparison of Outlier Residues^a^

source	detected outliers (residue IDs)
Ferraro et al. 2016	121, 125, **289**, 337, 362, **363**, 365, **366**, **367**, **369**, **495**, 531, 561, 572, 575, 579
POPC/CHOL	93, 122, 123, 126, 127, 166, 170, 171, 174, 191, 256, 264, 265, 268, 280, 283, 287, **289**, 293, 326, **363**, **366**, **367**, **369**, 370, 373, 429, 436, 454, 474, 475, 478, 479, **495**, 514, 577, 581, 584, 597
DSPC/POPE/CHOL	122, 123, 127, 163, 167, 170, 171, 174, 191, 253, 256, 265, 269, 280, 283, **289**, 313, 320, 326, **363**, **366**, **367**, **369**, 370, 373, 374, 436, 468, 474, 478, 479, **495**, 514, 584, 590, 597

a The table lists the residues identified by Ferraro et al.^16^ Alongside those detected in the two membrane compositions analyzed in this study. Residues consistently classified as outliers across all three datasets are highlighted in bold.

**Figure 2:**
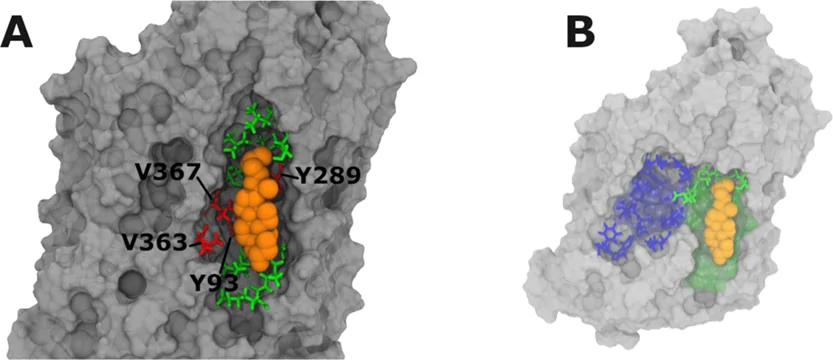
Results of the CHAMP protocol on the SERT transporter. (A) Crystallographic pocket (green licorice) of cholesterol (orange) in SERT. The residues consistently detected as outliers across all three data sets are highlighted in red and explicitly labeled. (B) Spatial overlap between previously reported cholesterol interaction sites (blue and green surfaces) and our top and fourth scoring (licorice blue and green, respectively) NanoShaper-detected pockets.

Some differences between our results and those of Ferraro et al.^16^ were expected. Their study employed longer simulations, more replicas, and a customized workflow, all of which can increase the separation between long-lived contacts and the central distribution, resulting in fewer residues being flagged as outliers. Additionally, previous work was based on a homology model, obtained from a bacterial member of the neurotransmitter sodium symporters family, the leucine transporter, and used a previous version of the Martini force field (Martini 2.2 for proteins and Martini 2.0 for lipids), both of which introduce inherent structural and methodological differences. Nonetheless, given that the goal of the present pipeline is to provide an automated and computationally efficient approach, the close correspondence with Ferraro’s more intensive setup supports both the robustness of the method and its suitability for large-scale analyses. Although our methodology inherently represents a compromise between speed and accuracy, the agreement observed here indicates that this trade-off remains well balanced for practical applications.

Analyzing the static structure of the transporter, NanoShaper identified 36 pockets, 16 of which met the geometric criteria for membrane-interface accessibility and were retained as cholesterol-binding candidates. These pockets were then ranked using the hot spot information derived from the contact outliers and SDF density points. The analysis was performed independently for both membrane compositions (Table 2).

**Table 2:** Top-Scoring NanoShaper Pockets Identified in SERT^a^

rank	pocket id	resIDs	system					
			POPC/CHOL			DSPC/POPE/CHOL		
			density points	outlier score	total score	density points	outlier score	total score
1	27	105 118 121 122 125 126 130 362 365 366 369 370 372 373 536 537	952	5000	5952	1504	6000	7504
2	33	184 187 261 262 265 280 283 284 287 429 432 433 436 440	1223	4000	5223	534	6000	6534
3	26	170 171 174 245 248 476 479 488 491 492 573 577 581	796	4000	4796	216	6000	6216
4	31	289 292 293 370 371 374	793	3000	3793	601	3000	3601
5	30	148 163 166 167 169 170 443 446 450 454 465 469 472 473	494	3000	3494	303	3000	3303

a The top five pockets are reported for the POPC/CHOL and DSPC/POPE/CHOL systems along with the corresponding number of density points and scores.

Comparison of the two membrane conditions showed that the top five ranked pockets were identical, indicating that membrane composition did not meaningfully alter the pocket ranking at the cholesterol ratio tested. The DSPC/POPE/CHOL system exhibited slightly higher scores for the top three pockets, but the differences were small and likely not sufficient to suggest a composition-dependent effect, particularly given that only four trajectories were analyzed. Notably, the pocket ranked fourth overlapped with the crystallographic cholesterol site. We next compared our highest-ranked pockets with the cholesterol-binding sites reported by Ferraro et al. Including the crystallographic site, two of the previously reported pockets were recovered. As shown in Figure 2B, our first-ranked pocket (licorice blue) and fourth-ranked pocket (licorice green) overlapped with the previous study’s K (blue surface) and C (green surface) sites, respectively.

To complement the static analysis with a dynamic characterization of pockets along the trajectory, NanoShaper was also applied in its Pocketron variant, as implemented in the BiKi Life Sciences software.^57^ Pocketron evaluates pockets on a frame-by-frame basis along the MD trajectory, enabling the monitoring of pocket appearance, persistence, and geometric evolution over time. The resulting pocket ranking was largely consistent with that obtained from the static NanoShaper analysis, with detailed results reported in the Supporting Information S2. Although the CG-MD simulations employ an elastic network, and therefore do not sample large-scale conformational rearrangements, the close agreement between static and dynamic pocket analyses indicates that the approach is robust to small local fluctuations, such as side-chain motions. This robustness comes at the explicit cost of restricting global protein flexibility, a trade-off that is inherent to the efficiency gains afforded by CG-MD. In light of this consistency, and to avoid unnecessary methodological redundancy, subsequent analyses were carried out using the static NanoShaper-based pocket identification.

Furthermore, several crystal structures of the transporter consistently report a cholesterol molecule bound to a region defined by residues 573, 576, 577, and 580.^47,58^ While the pocket analysis identifies a putative binding pocket in this region, it does not rank it among the top-scoring sites in terms of cholesterol-binding propensity (with the closest pocket ranking in the third position), suggesting that the limited conformational sampling intrinsic to the CG model may influence the relative prioritization of such sites. In order to assess this hypothesis, the full protocol and the NanoShaper analysis were also run on one of the crystal structures where the cholesterol appears in the aforementioned position, nearing residues 573–580 (PDB ID: 5I6X).^47^ Interestingly, as shown in Figure S1, the top scoring pocket corresponded to the region closest to the crystallographic cholesterol, formed by the residues 170, 171, 174, 245, 248, 476, 479, 488, 491, 492, 577 and 581, equivalent to that scoring third in the previous structure. Additionally, within the top 5 scoring pockets, equivalent cavities to those in the previous structure were identified. This analysis further strengthens the idea that while the starting structure of the same protein might have little influence in pocket detection itself, it is a key factor for the hot spot detection, as the analyses showed a certain level of preference in prioritizing the original pocket.

To evaluate the impact of simulation length on the identification of statistical outliers, we computed outliers at multiple time points for all SERT systems. As shown in Figures S12–S14, the principal residues delineating the main cholesterol-protein contacts are typically recovered within the first 10 μs of simulation. Nevertheless, additional outliers emerge at longer time scales, indicating that extended simulations are required to capture persistent interaction events that would be missed with insufficient sampling.

### Sodium–Potassium ATPase Pump

To further explore the transferability of the CHAMP protocol, as well as the ability to correctly identify membrane-exposed pockets bound by cholesterol, an additional membrane protein of similar size, corresponding to a sodium–potassium pump protein, was evaluated. The chosen structure (shark sodium/potassium-transporting ATPase α subunit, PDB ID: 3A3Y)^30^ presents a cholesterol bound near the surface of the protein. Our results, shown in detail in the Supporting Information S3, reveal that the canonical binding site for cholesterol is identified, ranking in the first position. The second-ranked pocket is as well occupied by a cholesterol molecule in a few crystal structures.^59^ Moreover, we identified other potential binding sites with relatively high scores, where at least three to five residues correspond to our identified statistical outliers. These results have been discussed in S3.

### Cannabinoid 1 Receptor

We next examined the CB_1_ receptor, as a representative class A GPCR characterized by allosteric modulators with pharmacological interest and identified binding pockets.^60^ We selected the crystal structure of CB_1_ receptor bound to the orthosteric agonist CP55940 and to the positive allosteric modulator ZCZ011,^31^ which also contains two resolved cholesterol molecules. This system is a suitable test case because both experimental studies and recent atomistic simulations have highlighted cholesterol as a modulator of CB_1_ receptor function, reporting specific interaction regions and long-lived binding events.^61,62^ Using CB_1_ receptor therefore allowed us to evaluate whether the proposed workflow can recover cholesterol-accessible sites in line with experimental observations and with results obtained from more computationally demanding methodologies.

Applying CHAMP, the structure was converted to CG using the Martini 3 topology, embedded in a POPC(75%)/CHOL(25%) membrane and simulated and analyzed on two replicas. After contact analysis, we obtained 27 outliers, with *t*_max_ ranging from 96.72 to 867.10 ns (Table 3). Overall, we identified three cholesterol-interaction hot spots at the protein–membrane interface, defined as regions where long-residence-time outlier residues and high SDF cholesterol density co-occur. Notably, two of these hot spots coincide with the cholesterol positions resolved in the crystal structure (colored in red and blue in Figure 3). In addition to these two expected regions, the analysis revealed a third area (green in Figure 3) showing a clear consensus between persistent contacts and enriched cholesterol density, suggesting a possible, previously unreported, interaction region. Although candidate site identification relies on a consensus criterion that provides a conservative and robust basis for highlighting cholesterol-interaction regions, whether this third site represents a genuine binding preference remains to be established experimentally.

**Table 3:** Outlier Residues Identified in CB_1_ Simulations after the Replica Merging

membrane	detected outliers (residue ID^BW^)
POPC/CHOL (75:25)	142^1.58^, 161^2.48^, 165^2.52^, 168^2.55^, 169^2.56^, 172^2.59^, 191^3.27^, 195^3.31^, 198^3.34^, 201^3.37^, 202^3.38^, 208^3.44^, 209^3.45^, 212^3.48^, 232^4.41^, 236^4.45^, 240^4.49^, 241^4.50^, 243^4.52^, 245^4.54^, 282^5.46^, 287^5.51^, 291^5.55^, 350^6.42^, 353^6.45^, 360^6.52^, 361^6.53^

**Figure 3:**
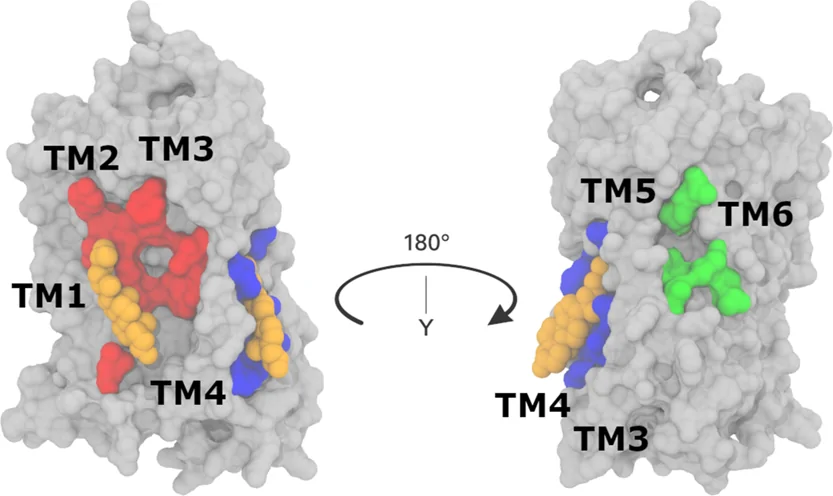
Hot spots detected in the CB_1_ receptor based on the agreement of outliers with reproducible density. For visual clarity, only the residues are shown, while the density cloud is omitted. Three distinct interaction areas are identified: two holding cholesterol molecules (red and blue pockets, centered around TM1-4 and TM3-5 respectively), and a third representing a putative, unreported, novel interaction site (in green, centered around TM5-6).

**Table 4:** Top Five Scoring Pockets Detected by NanoShaper for the CB_1_ Receptor

rank	pocket id	resIDs (outliers bolded)	system		
			cannabinoid receptor 1		
			density points	outlier score	total score
1	11	**165** ^**2.52**^ **169** ^**2.56**^ **172** ^**2.59**^ 190^3.26^ **191** ^**3.27**^ 192^3.28^ 194^3.30^ **195** ^**3.31**^ **198** ^**3.34**^ 199^3.35^ **241** ^**4.50**^ **245** ^**4.54**^ 248^4.57^ 249^4.58^ 252^4.61^	1761	8000	9761
2	15	**201** ^**3.37**^ 204^3.40^ 205^3.41^ **208** ^**3.44**^ **209** ^**3.45**^ **240** ^**4.49**^ **243** ^**4.52**^ 244^4.53^ 247^4.56^ 279^5.43^ **282** ^**5.46**^ 283^5.47^ 286^5.50^	1634	6000	7634
3	12	283^5.47^ 284^5.48^ **287** ^**5.51**^ 288^5.52^ **291** ^**5.55**^ 352^6.44^ **353** ^**6.45**^ 356^6.48^ 357^6.49^ 360^6.52^	1986	4000	5986
4	14	**291** ^**5.55**^ 294^5.58^ 295^5.59^ 346^6.38^ 349^6.41^ **350** ^**6.42**^ **353** ^**6.45**^	1232	3000	4232
5	9	138^1.54^ 141^1.57^ 142^1.58^ 148^98.83^ 153^2.40^ 154^2.41^ 157^2.44^ 158^2.45^	287	0	1287

One of the identified hot spots overlaps very well with the binding site reported as BS1 in a study by Vanegas and co-workers.^62^ This region includes TRP241^4.50^ (Ballesteros-Weinstein numbering superscript) This residue is the most highly conserved in TM4 across class A GPCRs and has been consistently reported to participate in cholesterol interactions,^63^ not only in CB_1_ receptor but also in other members of the subfamily, including β2AR^7,64^ or the nociceptin opioid peptide receptor.^65^ TRP241^4.50^ plays a key role in stabilizing the receptor’s transmembrane structure through hydrophobic contacts. Its side chain can form π–π interactions and hydrogen bonds, which help preserve the proper conformation of the receptor. Importantly, TRP241^4.50^ is involved in the recognition of allosteric modulators. Even the NAM Org27569 binds to a site outside the transmembrane core that includes TRP241^4.50^, with the indole group of Org27569 engaging in aromatic stacking with the indole side chain of TRP241^4.50^, underscoring the residue’s relevance in allostery-related methods. Furthermore, two more hot spots among those identified here partially overlap with the sites named BS2 and BS5 by Vanegas and colleagues.

We next characterized putative cholesterol-binding pockets at the protein–membrane interface and ranked them according to binding propensity, as shown in Figure 4. NanoShaper initially identified 16 geometrically defined cavities, which were reduced to 7 after applying the filtering criteria described in the Methods section. Importantly, the highest-ranked pockets (see Table 4) closely correspond to the interaction regions identified by the hot spot analysis, providing independent confirmation that the detected hot spots map onto bona fide, well-defined geometric pockets rather than diffuse regions of lipid enrichment. Specifically, the top-ranked (red in Figures 3 and 4) and fifth-ranked (yellow in Figures 3 and 4) pockets together correspond to the previously proposed CRAC binding site^2^ and encompass the conserved TRP241^4.50^ residue. Consistent with crystallographic evidence, one of the cocrystallized cholesterol molecules occupies this site. The second cholesterol molecule is located within the second-ranked pocket (blue in Figures 3 and 4), partially overlapping with the BS3 site reported by Vanegas and co-workers.^62^ Notably, the third-ranked pocket corresponds to the previously discussed hot spot that suggests a potential cholesterol interaction site that has not been previously reported. The correspondence between dynamically identified hot spots and independently detected geometric pockets renders these sites structurally well-defined and readily exploitable in structure-based drug design workflows, such as docking and pharmacophore modeling.

**Figure 4:**
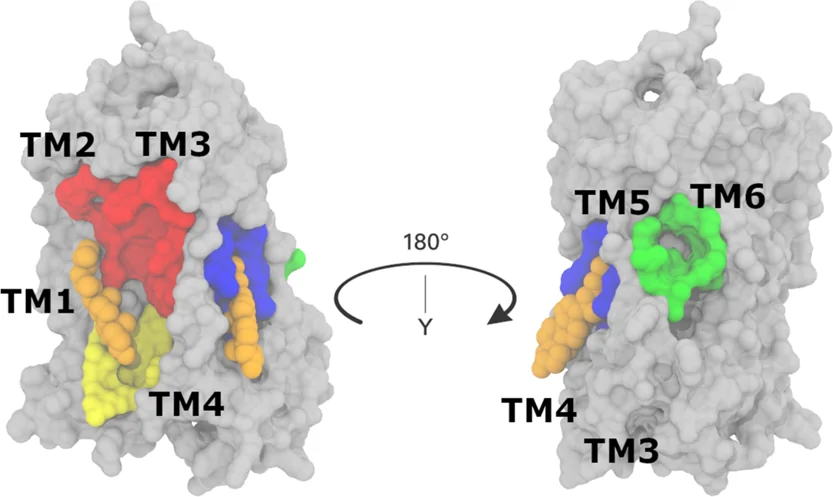
Spatial distribution of the top-scoring NanoShaper pockets in the CB_1_ receptor. The identified pockets are ranked according to their CHAMP cholesterol binding propensity and color-coded as follows: first (red), 2nd (blue), third (green), and 5th (yellow).

The cholesterol interaction sites characterized by CHAMP are consistent with the outcome of a recent Bayesian nonparametric analysis reported by Sexton et al.^66^ on CG-MD simulations. Specifically, some of the residues contacted by cholesterol with higher residence time in their work matched with those retrieved by our protocol, including residues bordering experimental pockets with bound cholesterol. Importantly, CHAMP successfully identified the experimentally observed cholesterol-binding pocket centered around the invariant TRP241^4.50^, assigning it the highest rank. Moreover, tracking the evolution of outliers over simulation time on both replicas, this binding pocket was already successfully characterized by 10 μs, showing stable contacts with several residues, i.e., LEU165^2.52^, ILE169^2.56^, TYR172^2.59^, ALA198^3.34^, and ILE245^4.54^, already identified by Hua et al. Conversely, other cholesterol interaction sites could only be recovered through the analysis of the full 30 μs trajectory. The results of these analyses are reported in Supporting Information S6. Consistently with data from SERT, these findings indicate that shorter simulations would risk missing relevant long-lived contacts. Conversely, the 30 μs simulation length for each replica ensures more robust and comprehensive outlier identification.

Finally, comparing our results with CB_1_ crystal structures featuring bound cholesterol, we found that three among our top five scoring pockets (namely, those ranked first, second, and fifth) matched the regions where cholesterol is consistently observed, as detailed in Supporting Information S7.

### Cannabinoid 1 Receptor Negative Allosteric Modulator

Our fully automated protocol was designed with cholesterol in mind, aiming to identify critical hot spots at the membrane–protein interface with potential allosteric implications. However, in principle, our framework can be readily extended to other allosteric modulators acting at the membrane–protein interface providing the framework for the identification of privileged interaction sites of small molecules and drug-like compounds. To provide a preliminary proof of concept in this direction, we applied the protocol to Org27569. This molecule is a NAM of the CB_1_ receptor.^67^ Additionally, a crystal structure of the CB_1_ – Org27569 complex is available to be used as a reference (PDB ID: 6KQI).^50^ The objective here is not to provide a comprehensive assessment across chemotypes, but to demonstrate the methodological extensibility of the framework beyond sterol-like molecules.

For system construction, we build upon a previous work by Bartocci and colleagues,^49^ where the authors studied allosteric cannabinoid binding at the glycine receptor α1. Specifically, we embedded the CB_1_ receptor in a POPC lipid membrane with 5% of Org27569 (i.e., 95:5 POPC/ligand ratio), corresponding to a ligand concentration of about 25 mM. We sought to determine whether at least one of the detected binding hot spots corresponded to the binding site observed in the crystal structure. Such semiquantitative validation would underscore the actual potential for prospective use.

Here, in analogy with Bartocci et al. and to increase the statistical robustness of the observation for this molecule, we performed 10 replicas of 10 μs each. By analyzing the trajectories generated by CHAMP, we identified the most persistent contacts between the CB_1_ receptor and the ligand Org27569. The main findings are summarized in Table 5, which reports residues from the CB_1_ receptor ranked according to how frequently they appear across replicas and the related timing information. Interestingly, in six out of ten runs the longest contact is established by residues belonging to (i.e., TRP241^4.50^) or in proximity of (i.e., GLY195^3.31^ and ALA202^3.38^) the allosteric site observed in the crystal complex.

**Table 5:** Ranking of the Most Persistent Contacts Established by Residues from the CB_1_ Receptor with the Org27569 Ligand^a^

residue	frequency	average duration (ns)	shortest duration (ns)	longest duration (ns)	most persistent in no. replicas
TRP241^4.50^	10	396	238	577	4
ALA202^3.38^	10	286	147	419	1
ALA198^3.34^	10	247	133	438	0
TYR172^2.59^	10	227	183	275	0
GLY195^3.31^	10	226	137	447	1
VAL161^2.48^	9	201	143	286	0
PHE208^3.44^	8	213	123	329	0
ILE243^4.52^	7	296	169	486	0
THR201^3.37^	7	242	142	337	0
PHE191^3.27^	7	144	100	215	0
VAL282^5.46^	6	344	145	718	1
PHE379^7.35^	3	1582	261	2892	2
SER284^5.48^	1	328	320	328	1

a The top ten residues are reported, with additional residues that exhibited highest persistency in at least one of the simulations.

Notably, all residues engaging persistent contacts in 90% of the simulations belong to the allosteric binding site. The only exception is TYR172^2.59^, which is still relatively close to the allosteric site. It is worth noting that TRP241^4.50^, a central residue in the allosteric site ranks first in Table 5, being observed in all ten CG-MD replicas while also displaying longest average duration. PHE379^7.35^ emerges as the residue with the longest average contact, although observed in only three runs. Its average and longest duration are particularly prolonged with 1582 and 2892 ns (Table 5), respectively, suggesting a stable interaction. This residue is located within the transmembrane helical bundle and forms part of the orthosteric binding site. Therefore, in some replicas with see the engagement of the orthosteric site by Org27569 (see Supporting Information S8), which has not been previously reported in the literature. However, the frequency of this interaction is substantially lower than that observed for residues within the allosteric site, indicating a clear preference for allosteric engagement. By aggregating the results from the ten replicas, ligand density maps projected on the atomistic structure of the CB_1_ receptor provide a straightforward visualization of prominent binding hot spots (Figure 5).

**Figure 5:**
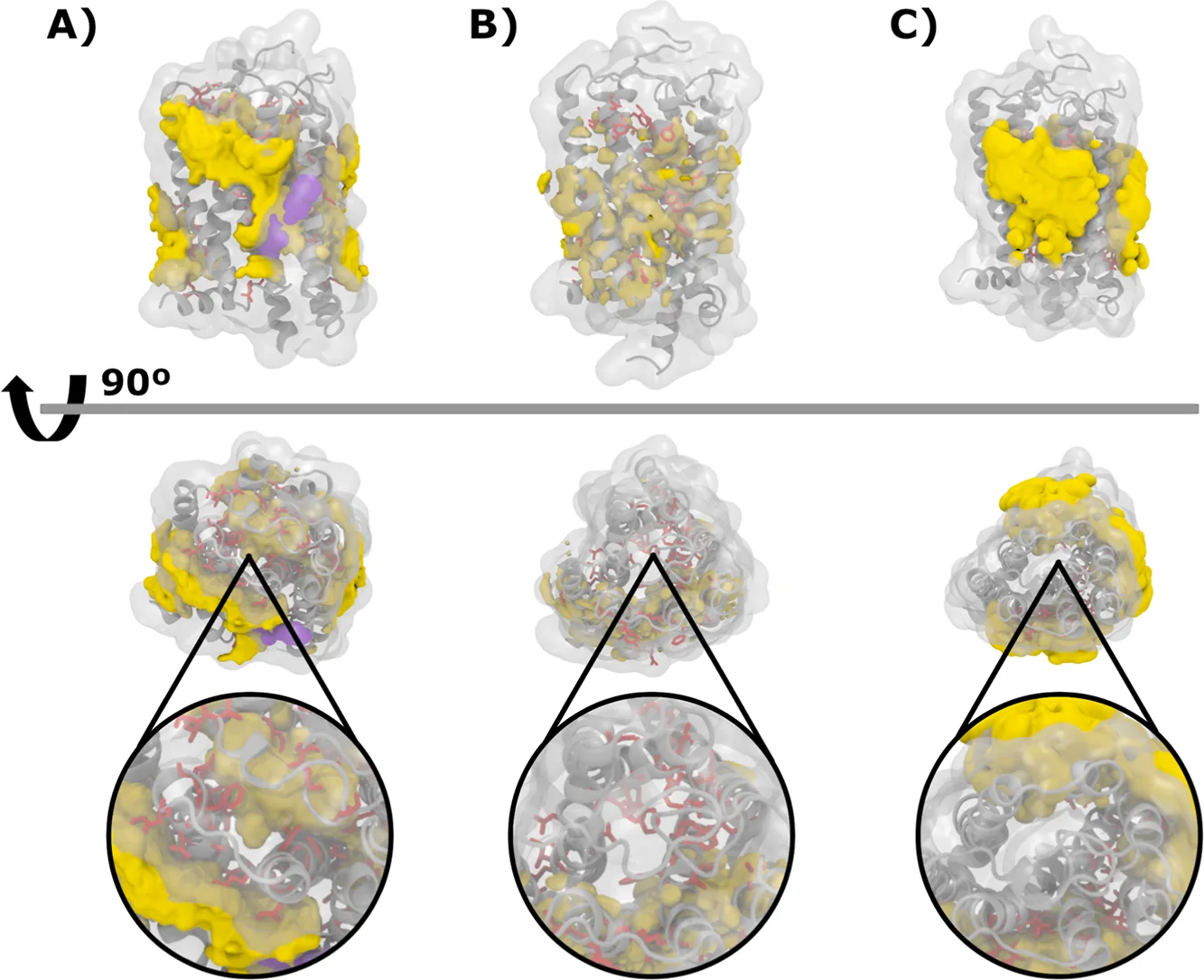
Ligand density over the CB_1_ receptor surface for (A) Org27569 and (B) Org27569 in the presence of THC in the orthosteric site, and (C) cholesterol. For all systems, both front and extracellular views are shown. In the extracellular view, an enlarged inset highlights the orthosteric binding site within the transmembrane helical bundle. Ligand densities (yellow) are mapped onto the corresponding CB_1_ receptor crystal structures used to construct the systems (PDB IDs: 6KQI,^51^ 9ERX,^68^ and 7FEE^31^ for panels A, B and C, respectively). In panel A, the crystallographic pose of Org27569 is shown in purple to indicate the corresponding allosteric binding site. Residues identified as statistically significant contact outliers are highlighted in red.

The results obtained for Org27569 were compared with those from the standard CHAMP protocol applied to cholesterol on the CB_1_ receptor (Figure 5C). Overall, the two profiles were qualitatively consistent. Although less pronounced than for Org27569 itself, cholesterol also showed significant sampling of the Org27569 binding site, whereas no significant cholesterol occupancy was detected within the orthosteric cavity. On the one hand, this observation underscores the ability of the pipeline to discriminate between different ligands. On the other hand, the comparison highlights a peculiar tropism of the allosteric modulator for the orthosteric binding site.

To further assess the tendency of Org27569 to occupy the orthosteric site in realistic conditions, we repeated the simulations in the presence of the active-site ligand tetrahydrocannabinol (THC). To this end, we used the complex between the CB_1_ receptor and THC and embedded it in the same membrane environment containing POPC and Org27569 as in the original simulations. As expected from five 10 μs exploratory simulations, Org27569 did not form contacts with residues from the orthosteric site of the receptor (see Supporting Information S9, Tables S12 and S13). This underscores the importance of performing simulations in realistic conditions to circumvent, and possibly discriminate, potential simulation artifacts. Additionally, despite the relatively more limited sampling, residues TRP241^4.50^ and VAL161^2.48^ from the allosteric site still displayed the most prolonged contact duration (Table S12). A graphical representation of these findings is reported in Figure S20, where we highlight the location of the residues within the 3D structure of the CB_1_ receptor.

As a final application, both CB_1_ structures used in this section, the one cocrystallized with THC (PDB ID: 9ERX) and the one without (PDB ID: 6KQI), were processed using NanoShaper. We used the CHAMP outcome to rank the detected pockets according to their predicted propensity to bind cholesterol. The highest scoring pocket belongs to the allosteric site of Org27569 (Figure 6), which overlaps with a predicted binding site for cholesterol. Interestingly, the other cholesterol binding site in CB_1_ (blue surface in Figure 6) also achieved a high score, ranking second in the system without THC and fourth when THC is present. Additionally, the rest of the pockets among the top scoring five also show significant agreement with those identified for cholesterol. This overlap between cholesterol and Org27569 hot spots could indicate similar behavior of both molecules around the CB_1_ receptor, as the cholesterol hot spots remain consistent across the available CB_1_ structures with bound cholesterol (see Tables 5 and S13).

**Figure 6:**
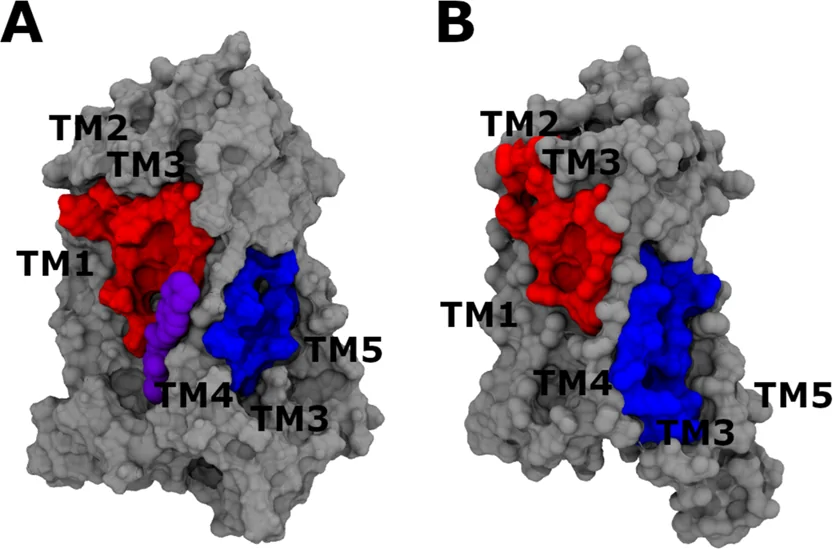
Comparison of overlapping cholesterol and Org27569 binding hot spots in the CB_1_ receptor. NanoShaper-detected pockets are ranked to identify spatial consensus between cholesterol and the Org27569 ligand. Results are shown for the CB_1_ receptor simulated in the absence (A, PDB ID: 6KQI) and the presence of THC (B, PDB ID: 9ERX).

## Conclusions and Perspective

In this work, we introduce CHAMP, a robust and largely automated pipeline for building and simulating CG protein–membrane systems with the aim of identifying cholesterol interaction hot spots. The only starting requirement is an oriented atomistic protein structure that underwent standard preprocessing steps. Through the systematic integration of several publicly available tools, the protocol reliably provided results that are consistent with previous computational and experimental studies. Demonstrating the flexibility of CHAMP, the pipeline could be readily applied, with minimal adaptations, to investigate two different membrane compositions and molecules other than cholesterol or membrane lipids. To this end, the CB1 receptor NAM Org27569 was used as a retrospective validation case, focusing on the identification of interaction hot spots. The protocol successfully prioritized the region surrounding TRP241^4.50^, corresponding to the known binding site of Org27569, thereby providing a proof of concept for its use with a nonlipid ligand. While previous approaches, such as that of Ferraro et al.,^16^ have demonstrated the effectiveness of combining contact persistence and density analysis to identify cholesterol interaction sites, their application has remained largely system-specific and required significant manual intervention. In contrast, CHAMP is designed as a fully automated and standardized workflow that integrates system preparation, simulation, analysis, and hot spot ranking into a unified framework. By bridging the gap between automated setup of simulations and state-of-the-art analysis tools for protein–membrane interactions, our pipeline enables reproducible application across multiple targets and membrane environments, making the protocol suitable for large-scale and comparative studies of membrane proteins. Furthermore, the use of CHAMP does not require extensive expertise in computational chemistry, programming, or MD simulation software. At the same time, CHAMP can also be run in a modular fashion, allowing more experienced users to customize specific stages of the workflow or to perform the analyses on MD trajectories generated independently of the CHAMP pipeline.

A key limitation of the current implementation of CHAMP is the use of ENM restraints in CG-MD simulations, which preserve structural integrity but limit large-scale conformational sampling. As a result, the method is primarily sensitive to interaction sites accessible within the conformational ensemble represented by the starting structure. For highly dynamic systems, this limitation can be mitigated by applying the protocol to multiple receptor conformations, either derived from experimental structures or generated through enhanced-sampling simulations.

The availability of the pipeline as an installable Python package makes it an accessible and user-friendly tool that is readily deployable for novel applications involving ligand interactions at the protein–membrane interface. For example, more than 500 class A GPCR structures are publicly available, including over 200 that contain at least one resolved cholesterol molecule.^69^ A systematic application of CHAMP to this set would enable not only a robust retrospective assessment of known cholesterol binding sites, but also the identification of previously unrecognized sites and their comparative classification across receptor families. On this basis, future work will focus on applying the protocol to larger data sets to comprehensively characterize cholesterol interaction sites at the membrane interface within protein families.

## Supplementary Material





## Data Availability

All source code is available in the dedicated GitHub repository of the Computer-assisted Molecular Design (CAMD) laboratory, University of Urbino, at https://github.com/Urbino-CAMD-Lab/CHAMP. The repository includes full documentation, a comprehensive step-by-step tutorial describing both the fully automated and modular workflows (see CHAMP/tutorial_and_outputs/tutorial.md), and a minimal example data set illustrating the output directory structure and generated data. To facilitate reproduction of the reported results, full trajectories for a representative system have been deposited in Zenodo with accession code 10.5281/zenodo.19555568. Deposition of the complete MD trajectory data set in a public repository is currently not feasible because of its size. Nevertheless, the full set of trajectories is available from the corresponding author upon reasonable request. CG-MD topology and parameters of Org27569 are deposited in the Martini Database (https://mad.ens-lyon.fr/explore).

## Notes

Identification and characterization of SERT pockets bound to cholesterol; analysis of the sodium–potassium pump bound to cholesterol; CG-MD parameters of Org27569 and their validation; impact of trajectory striding in contact analysis; analysis of residue outliers over simulation time for SERT and CB_1_ receptor; comparison of cholesterol binding pockets identified via CG-MD simulations with experimental structures; and analysis of CG-MD simulations in which Org27569 binds to the orthosteric site (PDF)
Step-by-step tutorial describing the complete CHAMP workflow, covering system setup, MD simulations, hot spot analysis, and advanced modular usage (PDF)
