# Supplementary material for: Cholesterol Hot Spot Automated Mapping Protocol (CHAMP): Identifying Cholesterol-Binding Hot Spots in Membrane Proteins
Source: J Chem Inf Model. 2026 Sep 23;66(19):12667–81. doi: 10.1021/acs.jcim.6c01419 (PMC13693706; doi:10.1021/acs.jcim.6c01419)
Supplement: Supplementary file 1 [file ci6c01419_si_001.pdf]

SUPPORTING INFORMATION FILE 1

Cholesterol Hot spot Automated Mapping Protocol: Identifying Cholesterol-Binding Hot Spots in Membrane Proteins

David Sotillo-Núñez[1], Matteo Nardi Cesarini[1], Gian Marco Elisi[1], Mattia Bernetti[1], Giovanni Bottegoni[1,2]*

[1]Dipartimento di Scienze Biomolecolari, Università degli Studi di Urbino Carlo Bo, Via Ca' Le Suore, 2/4, 61029 Urbino, Italy.

[2]School of Pharmacy, University of Birmingham, Edgbaston, B15 2TT, Birmingham, United Kingdom.

[*] Corresponding author.

Email address: giovanni.bottegoni@uniurb.it

Index of Supporting Information File 1

S1. Pocketron analysis of SERT trajectories 3

S2. Analysis on a SERT structure with alternative cholesterol pocket 6

S3. Analysis on the shark sodium-potassium pump structure with a cholesterol bound 8

S4. Org27569 parametrization and validation 12

4.1 Full-atom system preparation and parametrization of Org27569 12

4.2 Atomistic simulations of Org27569 in water 12

4.3 Org27569 conformational analysis from atomistic simulations 13

4.4 Atom-to-bead mapping and definition of bonded terms for the ligand Org27569 16

4.5 CG-MD simulations of Org27569 in water 22

4.6 Comparison of Org27569 bonded parameter distribution between HREX full-atom and CG MD simulations 22

S5. Impact of trajectory striding on density map quality and pocket ranking 25

S6. Analysis of residue outliers over simulation time 30

S7. Comparison of the cholesterol pockets found with the pipeline with the positions in experimental structures 34

S8. Org27569 dynamics within the orthosteric binding site 35

S9. Org27569 contact analysis from the CG MD simulations with occupied orthosteric site ..37

S1. Pocketron analysis of SERT trajectories

Pocketron evaluates pockets on every MD frame, allowing their appearance, persistence, and geometric evolution to be monitored over time. To this aim, a protein-centered, strided trajectory generated during SDF preparation was sampled by saving every 10.000th frame. Each frame was backmapped into its atomistic representation using CG2AT ([1]) and the resulting structures were concatenated into a trajectory approximating the protein's conformational fluctuations during the CG simulation. Nonrelevant cavities were removed using the same geometric filters applied in the static NanoShaper analysis, excluding pockets with centroids too close to the protein's vertical axis or outside the transmembrane region. Pocketron also reports how frequently each residue contributes to a pocket; residues contributing to fewer than 10 frames were discarded. Because the dynamic analysis often produces multiple closely related cavity geometries corresponding to the same site, pockets were consolidated when their centroids fell within 7 Å and when they shared at least 40% residue overlap (using the smaller pocket as reference). This consolidation reduced redundancy and yielded a set of unique membrane-interface pockets suitable for subsequent scoring with the outlier residues and density points identified earlier in the protocol.

The Pocketron analysis initially identified 93 cavities along the trajectory. After applying the geometric filters and residue-contribution threshold, 43 pockets remained. The consolidation procedure reduced the final set to 27 unique membrane-interface pockets. The top-ranked pockets (Table S1) closely matched those obtained from the static NanoShaper analysis, although the dynamic evaluation yielded larger and deeper cavities, reflecting the conformational fluctuations sampled during the CG simulation.

[1] Vickery, O. N.; Stansfeld, P. J. CG2AT2: an enhanced fragment-based approach for serial multi-scale molecular dynamics simulations. *J. Chem. Theory Comput.* **2021**, 17, 6472– 6482, DOI: 10.1021/acs.jctc.1c00295

Table S1. Filtered and ranked clusters of Pocketron pockets scored using the CHAMP analysis output in both SERT systems. The column "Pocket id" indicates which of the original cavities identified by Pocketron were merged in the clustering step.

System	Pocket id	ResIDs	Contributing Points	Outlier Score	Total Score
POPC:chol	20_27_28_33_88	100 101 104 105 106 109 110 114 115 117 118 121 122 125 126 130 309 312 313 332 333 336 337 362 365 366 368 369 370 372 373 377 380 383	1171	6000	7171
	12_26_72	170 171 174 244 245 246 247 248 476 479 480 482 484 487 488 491 492 495 573 577 581	116	7000	7116
	23_58_59	93 94 97 101 102 285 288 289 292 293 367 370 371 372 374 375 430 433 434 437 438	726	5000	5726
	16	126 127 130 131 134 358 362 536 537 539 540 544	1418	2000	3418
	38_54	180 184 262 265 266 269 277 280 432 436 437 440	240	3000	3240
DSPC:POPE:chol	20_27_28_33_88	100 101 104 105 106 109 110 114 115 117 118 121 122 125 126 130 309 312 313 332 333 336 337 362 365 366 368 369 370 372 373 377 380 383	767	6000	6767
	12_26_72	170 171 174 244 245 246 247 248 476 479 480 482 484 487 488 491 492 495 573 577 581	607	5000	5607

	23_58_59	93 94 97 101 102 285 288 289 292 293 367 370 371 372 374 375 430 433 434 437 438	786	4000	4786
	38_54	180 184 262 265 266 269 277 280 432 436 437 440	533	4000	4533
	47	251 256 259 260 263 474 477 478 481	412	3000	3412

S2. Analysis on a SERT structure with alternative cholesterol pocket

In the main text, we used a serotonin transporter structure with a cholesterol molecule present to test and validate our method. However, other structures where cholesterol appears to be bound in a different position also exist. To determine the effect of the original position of the cholesterol in the results obtained using this protocol, we analyzed one of the structures where the cholesterol molecule is crystalized within the vicinity of residues 573, 576, 577, and 580. PDB id 5I6X[2] was selected for simulation and analysis. The results, shown in Figure S1 and Tables S2 and S3, are consistent with those obtained for the other SERT systems.

Most of the top five scoring pockets identified here closely matched those found in the structure used for the original analysis, with minor variations in cavity size and shape arising from the use of a different crystal structure. Although the same highest-scoring pockets were identified overall, their relative ordering changed. In particular, the top-ranked pocket, defined by residues 170, 171, 174, 245, 248, 476, 479, 488, 491, 492, 577, and 581, corresponds to the pocket closest to the crystallographic cholesterol position, whereas it ranked third in the previous structure. The pocket previously ranked second remained second, the pocket previously ranked fourth was not detected, and the pocket previously ranked fifth shifted to fourth.

Table S2. Outliers detected for the alternative SERT structure. Bolded residues are those also present in the other 3 systems: both membrane compositions and in Ferraro and colleagues' work.

Source	Detected outliers (residue IDs)
**PDB ID: 5I6X**	127, 170, 171, 191, 251, 256, 265, 280, 282, 283, 286, **289**, 326, **363**, **366**, **367**, **369**, 374, 429, 436, 458, 469, 474, 478, 479, 514, 577, 580, 581, 584, 586, 597

2 Coleman, J., Green, E. & Gouaux, E. X-ray structures and mechanism of the human serotonin transporter. *Nature*. **2016**, 532, 334–339. DOI: 10.1038/nature17629.

Table S3. Top 5 scoring NanoShaper pockets for the alternative SERT structure.

Rank	Pocket id	ResIDs	Contributing Points	Outlier Score	Total Score
1	16	170 171 174 245 248 476 479 488 491 492 577 581	857	5000	5857
2	22	184 187 261 262 265 280 283 284 287 429 432 433 436	765	5000	5765
3	18	118 121 122 125 126 130 362 365 366 369 370 373 537	1268	2000	3268
4	19	166 169 170 443 446 450 465 469 472 473	468	2000	2468
5	24	123 124 127 544 547 548 551	676	1000	1676

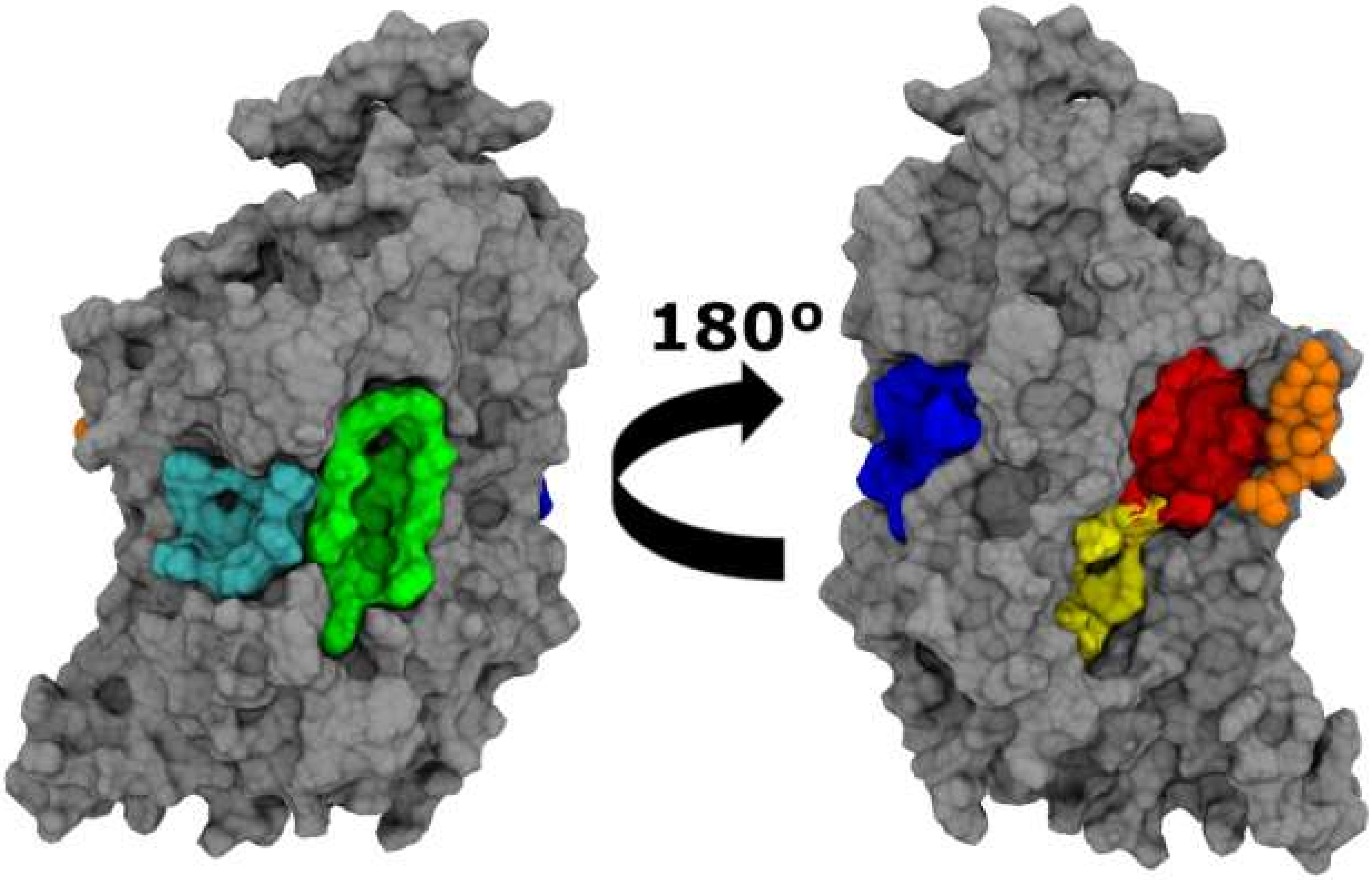

Figure S1. Top 5 scoring NanoShaper pockets. As represented in the figure, ranked from first to fifth: red, blue, green, yellow, cyan. Cholesterol is colored in orange. The top-ranked pocket (red) is also the closest the original position of the cholesterol molecule. The pocket corresponding to the cholesterol binding location in the other SERT structure is ranked in third position.

S3. Analysis on the shark sodium-potassium pump structure with a cholesterol bound

To further assess the applicability of CHAMP across different classes of membrane proteins, we applied the same protocol to the sodium–potassium pump (PDB ID: 3A3Y),[3] which presents a cholesterol in the vicinity of the protein surface on the transmembrane region. The 40 relevant residues for cholesterol contacts which were labeled as statistical outliers are shown in Table S4. The NanoShaper run identified 23 pockets.

One of the identified pockets (Pocket 1) was substantially larger than the others, extending over a broad region of the protein surface. This pocket encompassed the crystallographic cholesterol-binding site, centered around M7, and, owing to its larger size, accumulated more outlier residues than the remaining pockets, resulting in the highest overall score. The second-ranked pocket also corresponded to an experimentally reported cholesterol-binding site. The complete pocket ranking is reported in Table S5, and the spatial distribution of the pockets is shown in Figure S2A.

Because the highest-ranked pocket was substantially larger than the remaining cavities, we assessed whether its size alone influenced the scoring. The centroid of the pocket coincided with the crystallographic cholesterol position, confirming that the cavity indeed encompassed the experimentally observed binding site. To reduce the potential contribution of pocket size to the score, we defined a subset of this cavity consisting only of the residues surrounding the crystallographic cholesterol (blue surface in Figure S2, both panels). Under this alternative definition, the first two pockets exchanged positions in the ranking (Table S5, "Subset"), yet both experimentally observed cholesterol-binding pockets still remained the two highest-ranked sites. Thus, the overall conclusions of the analysis were unaffected by the definition of the larger pocket. For completeness, Figure S3 compares the identified pockets with the cholesterol molecules resolved in the available crystal structures.

Table S4. Outliers detected for the sodium-potassium pump.

Source	Detected outliers (residue IDs)
**PDB ID: 3A3Y**	106, 135, 142, 146, 298, 300, 304, 324, 338, 788, 791, 809, 812, 813, 816, 854, 859, 866, 931, 932, 934, 935, 944, 960, 962, 964, 966, 988, 989, 992, 996, 997, 999, 1000, 1001, 1004, 1016, 1040, 1042, 1047

3 Ogawa, H.; Shinoda, T.; Cornelius, F.; Toyoshima, C. Crystal Structure of the Sodium–Potassium Pump (Na^+, K^+ ATPase) with Bound Potassium and Ouabain. *Proc. Natl. Acad. Sci. U.S.A.* **2009**, 106 (33), 13742–13747. DOI: 10.1073/pnas.0907054106.

Table S5. Top 5 scoring NanoShaper pockets for the sodium-potassium pump. Pockets are ranked either based on the default selection of pocket 1 (Whole) or on a refined criterion that selects a specific subset centered around the centroid (Subset).

<table>
<tr><th>Pocket selection</th><th>Rank</th><th>Pocket id</th><th>ResIDs</th><th>Contributing Points</th><th>Outlier Score</th><th>Total Score</th></tr>
<tr><td rowspan="5">Whole</td><td>1</td><td>7</td><td>290 294 298 301 302 306 766 767 769 770 771 773 774 784 787 788 791 792 795 796 798 826 827 828 829 830 831 833 834 835 836 837 841 842 843 846 847 848 850 851 854 855 856 859 862 863 866 870 880 881 884 940 941 997 1000 1001 1004 1005 1008 1009 1016 1017 1018 1019 1020 1021 1022 1023 1024 1025 1028 1029 1032 1033 1035 1036 1038 1039 1040 1042 1043 1044 1046 1047 1048 1051 1052 1055</td><td>719</td><td>14000</td><td>14719</td></tr>
<tr><td>2</td><td>14</td><td>928 931 932 935 992 995 996 999</td><td>301</td><td>6000</td><td>6301</td></tr>
<tr><td>3</td><td>20</td><td>139 142 143 146 334 335 338 812 816</td><td>954</td><td>5000</td><td>5954</td></tr>
<tr><td>4</td><td>21</td><td>813 814 816 817 818 960 961 964</td><td>1451</td><td>4000</td><td>5451</td></tr>
<tr><td>5</td><td>18</td><td>103 106 299 300 303 324 325 328 332</td><td>274</td><td>3000</td><td>3274</td></tr>
<tr><td rowspan="5">Subset</td><td>1</td><td>14</td><td>928 931 932 935 992 995 996 999</td><td>877</td><td>6000</td><td>6877</td></tr>
<tr><td>2</td><td>7</td><td>290 294 297 298 301 302 305 306 309 784 787 788 791 792 846 847 848 850 851 854 855 856 859 862 863 866</td><td>301</td><td>6000</td><td>6301</td></tr>
<tr><td>3</td><td>20</td><td>139 142 143 146 334 335 338 812 816</td><td>954</td><td>5000</td><td>5954</td></tr>
<tr><td>4</td><td>21</td><td>813 814 816 817 818 960 961 964</td><td>1451</td><td>4000</td><td>5451</td></tr>
<tr><td>5</td><td>18</td><td>103 106 299 300 303 324 325 328 332</td><td>274</td><td>3000</td><td>3274</td></tr>
</table>

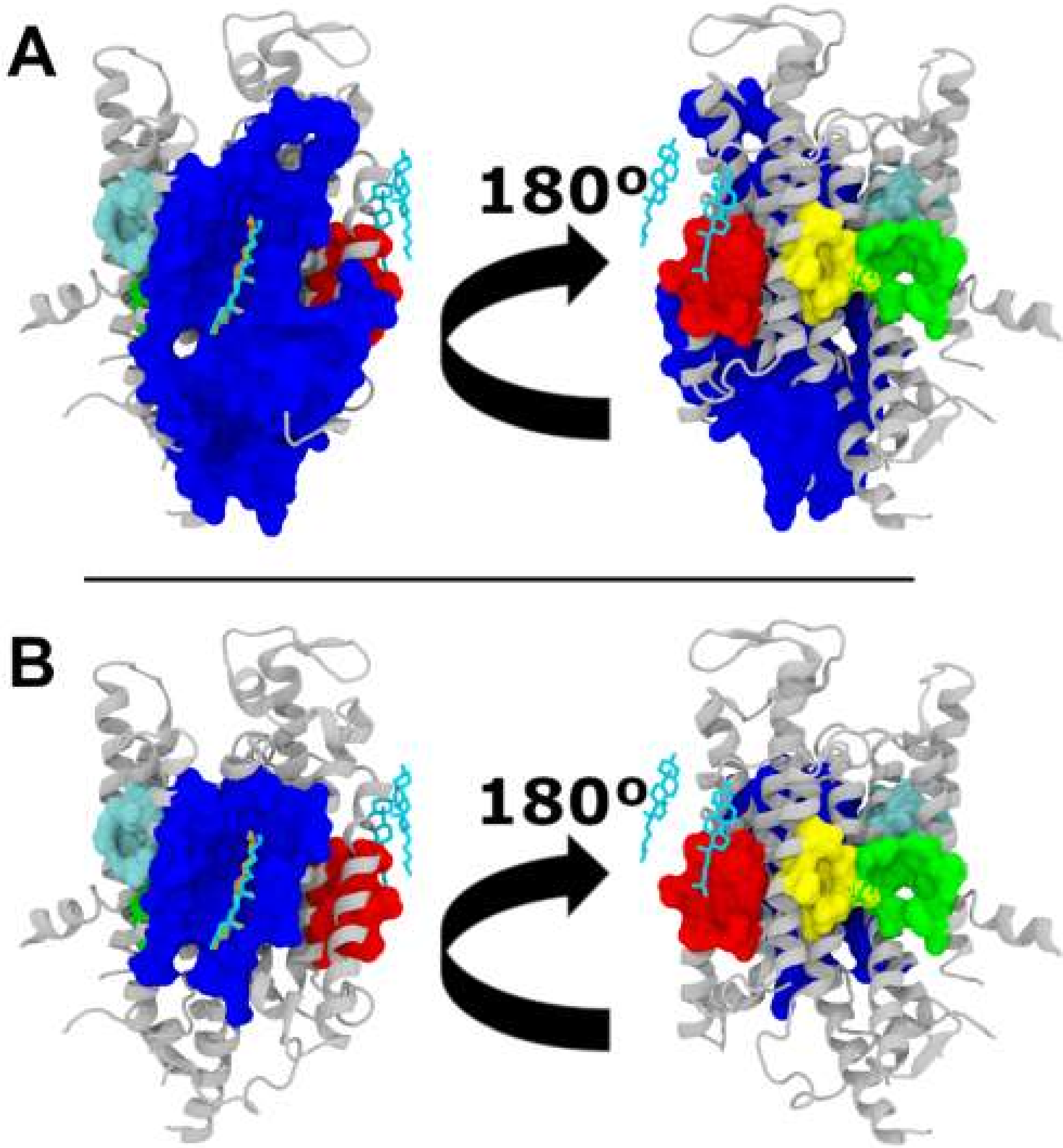

Figure S2. Top five scoring NanoShaper pockets. Cholesterol is colored in orange for those corresponding to 3A3Y and in cyan for those in 7WYU.[4] The blue pocket is always bound by cholesterol in the crystal structure, while the red one is a second site present in some of the available structures. A. Results from the automatic NanoShaper analysis. As represented in the figure, ranked from first to fifth: blue, red, green, yellow, cyan. B. Results with the revised selection of residues identifying pocket 1. In this case, the ranking is changed for the first two positions (the first is red, the second one is blue).

4 Kanai, R.; Cornelius, F.; Vilsen, B.; Toyoshima, C. Cryoelectron Microscopy of Na^+, K^+ ATPase in the Two E2P States with and without Cardiotonic Steroids. *Proc. Natl. Acad. Sci. U.S.A.* **2022**, 119 (15), e2123226119. DOI: 10.1073/pnas.2123226119.

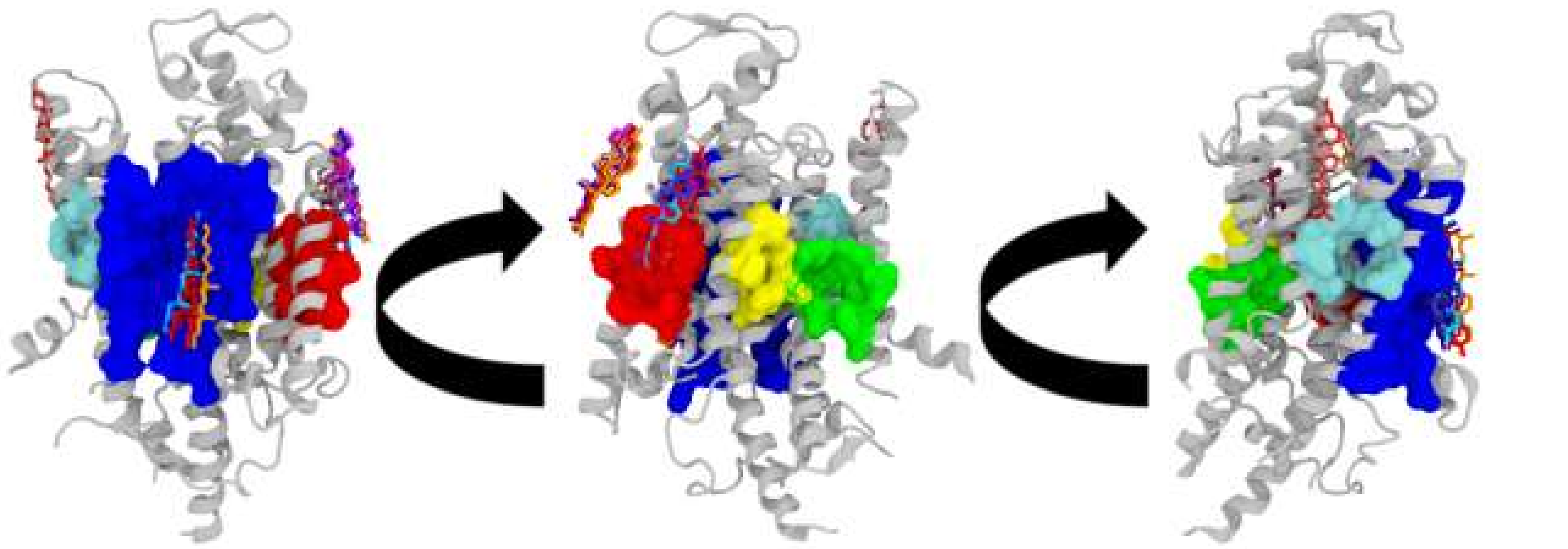

Figure S3. Overlap of every available sodium/potassium-transporting ATPase structure with bound cholesterol. Cholesterols included in the picture correspond to the structures with PDB IDs: 2ZXE, 3A3Y, 5AVQ, 5AVR, 5AVS, 5AVT, 5AVU, 5AVV, 5AVW, 5AVX, 5AVY, 5AVZ, 5AW0, 5AW1, 5AW2, 5AW3, 5AW4, 5AW5, 5AW6, 5AW7, 5AW8, 5AW9, 7WYU, 7WYV, 7WYW, 7WYX, 7WYY, 7WYZ, 7WZ0, 7Y45, 7Y46, 8JFZ, 9VIY, 9VIZ, 9VJ0 and 9VJ1.

S4. Org27569 parametrization and validation

4.1 Full-atom system preparation and parametrization of Org27569

All simulations were carried out with GROMACS 2021.5. ([5]) The ligand Org27569 was parametrized using the GAFF2 force field ([6]) and further solvated in a cubic water box of 38 $Å^3$ modelled with the TIP3P water model. ([7]) Ligand atomic partial charges were obtained through the atomic electrostatic potential charges (ESP) procedure implemented in Jaguar ([8]) on the coordinates retrieved from the X-ray crystal structure 6KQI ([9]) and minimized with MacroModel ([10]) in implicit water solvent with the conjugate gradient method. Specifically, the procedure consists of a single-point energy calculation performed at B3LYP/6-31G** level of theory, including Grimme's D3 empirical correction for long-range London dispersion forces. ([11])

4.2 Atomistic simulations of Org27569 in water

Conformational dynamics of the ligand was probed by performing three replicas of unbiased atomistic MD simulations, lasting 500 ns each and using the Gromacs 2021.5 MD engine ([5]) and setting a temperature of 298.15 K.

---

[5] Abraham, M. J.; Murtola, T.; Schulz, R.; Pall, S.; Smith, J. C.; Hess, B.; Lindah, E. Gromacs: High Performance Molecular Simulations through Multi-Level Parallelism from Laptops to Supercomputers. *SoftwareX* **2015**, 1–2, 19– 25, DOI: 10.1016/j.softx.2015.06.001

[6] Wang, J.; Wolf, R. M.; Caldwell, J. W.; Kollman, P. A.; Case, D. A. Development and testing of a general amber force field. *J. Comput. Chem.* **2004**, 25, 1157– 74, DOI: 10.1002/jcc.20035

[7] Jorgensen, W. L.; Chandrasekhar, J.; Madura, J. D.; Impey, R. W.; Klein, M. L. Comparison of Simple Potential Functions for Simulating Liquid Water. *J Chem Phys* **1983**, 79 (2), 926–935. DOI: 10.1063/1.445869.

[8] Schrödinger Release 2024-3: Jaguar, Schrödinger, LLC, New York, NY, 2024.

[9] Shao, Z.; Yan, W.; Chapman, K.; Ramesh, K.; Ferrell, A. J.; Yin, J.; Wang, X.; Xu, Q.; Rosenbaum, D. M. Structure of an Allosteric Modulator Bound to the CB1 Cannabinoid Receptor. *Nat. Chem. Biol.* **2019**, 15 (12), 1199– 1205, DOI: 10.1038/s41589-019-0387-2.

[10] Schrödinger Release 2024-3: MacroModel, Schrödinger, LLC, New York, NY, 2024.

[11] Grimme, S.; Antony, J.; Ehrlich, S.; Krieg, H. A Consistent and Accurate Ab Initio Parametrization of Density Functional Dispersion (DFT-D) for the 94 Elements H−Pu. *J Chem Phys* **2010**, 132 (15). DOI: 10.1063/1.3382344.

To achieve a more extensive sampling of ligand conformations, we used the Hamiltonian replica-exchange (HREX) method ([12]) with scaling applied to solute atoms, ([13]) and prepared the necessary ligand topologies with Plumed 2.9. ([14]) The HREX simulation consisted of five parallel replicas with lambda scaling factors of 1, 0.878752, 0.772205, 0.678576, 0.5963, corresponding to an "effective" temperature range from 298.15 K to 500 K. Each replica was simulated for 250 ns, resulting in an average replica exchange of 57%. The non-scaled (equivalent to room temperature) replica was used for further analysis.

Production simulations were conducted in the NPT and NVT ensembles for the unbiased and HREX MD simulations, respectively, and employing the v-rescale thermostat ([15]) and the c-rescale barostat ([16]) to control temperature and pressure. Prior to production, the system was equilibrated through the following steps: energy minimization consisting of 1000 steps with the steepest descent algorithm, followed by two equilibration phases of 500 ps each, the first in the NVT ensemble with the v-rescale thermostat, and the second in the NPT ensemble with the v-rescale thermostat and c-rescale barostat.

4.3 Org27569 conformational analysis from atomistic simulations

For a straightforward comparison of the conformational space explored by the unbiased and HREX simulations, the resulting trajectories were aggregated and subjected to principal component analysis (PCA). This was also intended at assessing the possible enhancement of sampling in the HREX simulation. As a first stage, all trajectory frames were aligned to the equilibrated reference structure of the ligand in order to remove irrelevant rotational and translational motions. Secondly, PCA was performed using the Cartesian coordinates of ligand heavy atoms as input features and taking into account possible symmetry in ligand conformations. From the PCA of the concatenated trajectories,

---

[12] Sugita Y., Okamoto Y. Replica-exchange multicanonical algorithm and multicanonical replica-exchange method for simulating systems with rough energy landscape. *Chem. Phys. Lett.* **2000**; 329:261–270. DOI: 10.1016/S0009-2614(00)00999-4.

[13] Bussi G. Hamiltonian replica exchange in GROMACS: a flexible implementation. *Mol. Phys.* **2014**; 112:379–384. DOI: 10.1080/00268976.2013.824126.

[14] The PLUMED consortium Promoting transparency and reproducibility in enhanced molecular simulations. *Nat. Methods* **2019**, 16, 670– 673, DOI: 10.1038/s41592-019-0506-8

[15] Bussi, G.; Donadio, D.; Parrinello, M. Canonical Sampling through Velocity Rescaling. *J. Chem. Phys.* **2007**, 126 (1), 014101, DOI: 10.1063/1.2408420

[16] Bernetti, M.; Bussi, G. Pressure Control Using Stochastic Cell Rescaling. *J. Chem. Phys.* **2020**, 153 (11), 114107, DOI: 10.1063/5.0020514

the first five principal components (PC) were retained for analysis. Figure S4 illustrates the projection of the data onto the first two PCs, which together explain 63% of the total variance. The frames from the three unbiased trajectories are all shown in red, while those from the HREX MD simulation are colored in grey. As expected, a broader distribution of the data points from the HREX across the PCA space confirms a wider exploration of the conformational space by the ligand. Thus, the HREX was selected as the reference trajectory for subsequent validation of ligand parametrization.

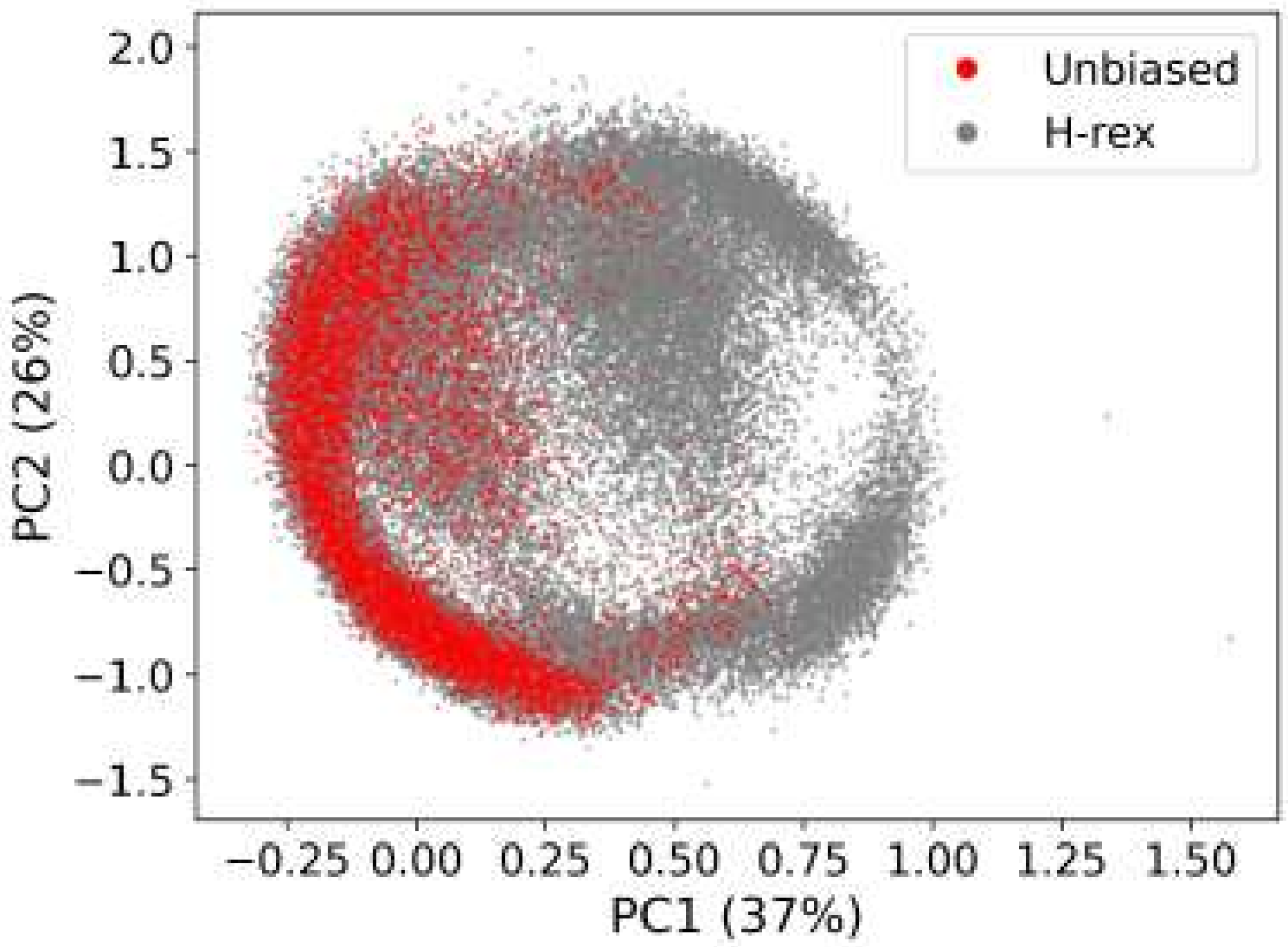

Figure S4. PCA on the unbiased (red) and HREX (grey) trajectories.

To identify the most representative atomistic conformations of the ligand we performed a cluster analysis. In particular, we employed the Quality Threshold (QT) clustering algorithm. ([17]) QT clustering was performed on the pairwise RMSD matrix over all frames of the HREX trajectory, after optimal alignment of each structure pair, ([18]) thus removing the effects of rotational and translational motions, considering only internal conformational rearrangements. Clusters were then ranked by population size, and the first five most populated clusters were selected to provide a representative set of conformations. The resulting representative structures are displayed in Figure S5, where the distribution of cluster members is projected onto the PCA space. In the plot, clusters are highlighted in different colors, thereby illustrating how the identified conformations are distributed across the ligand conformational landscape.

---

[17] González-Alemán, R.; Hernández-Castillo, D.; Caballero, J.; Montero-Cabrera, L. A. Quality Threshold Clustering of Molecular Dynamics: A Word of Caution. *J. Chem. Inf. Model.* **2020**, 60, 467– 472, DOI: 10.1021/acs.jcim.9b00558

[18] Kearsley, S. L. On the orthogonal transformation used for structural comparisons. *Acta Cryst. A* **1989**, 45, 208– 210, DOI: 10.1107/S0108767388010128

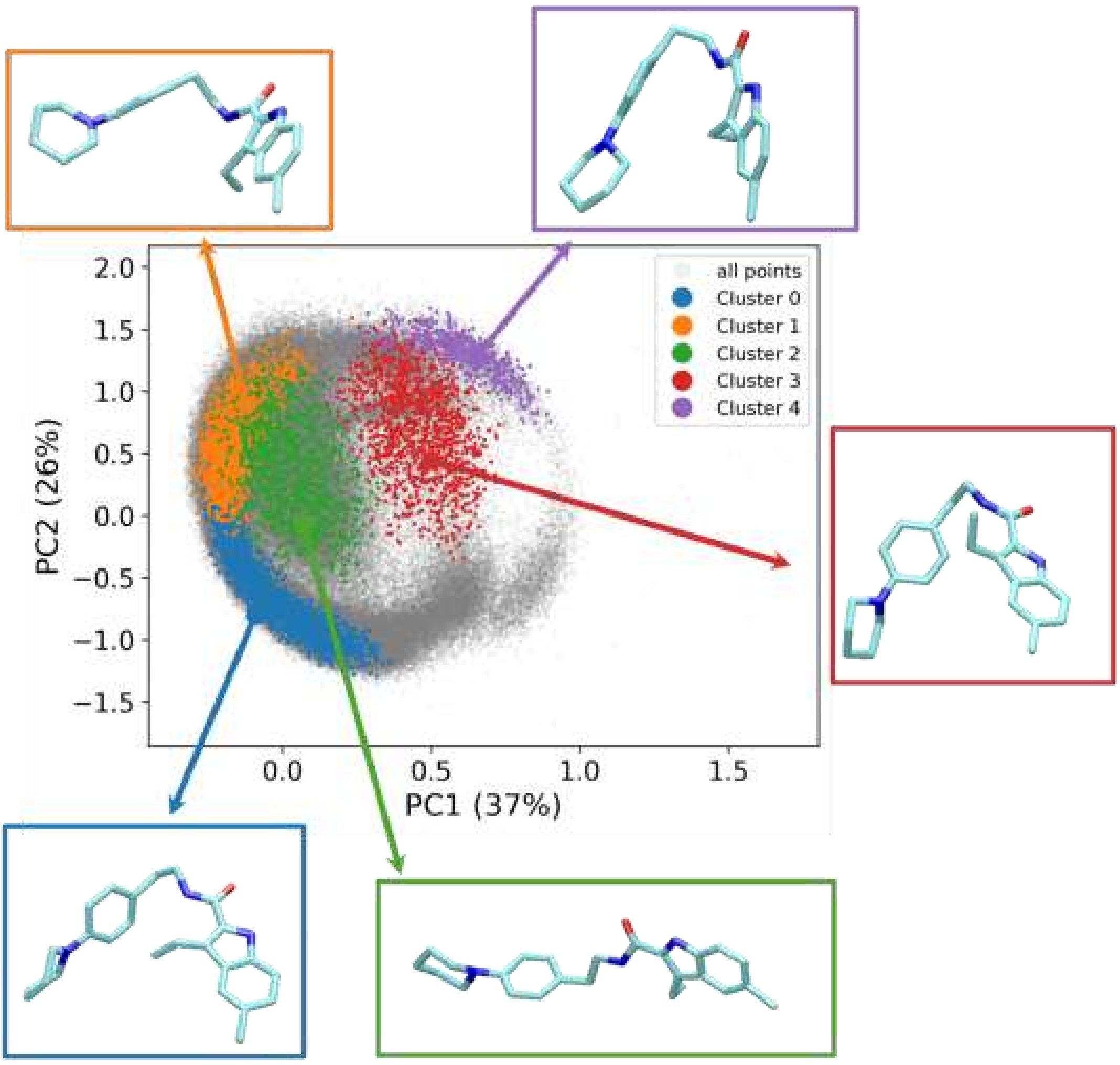

Figure S5. Top-5 clusters from the HREX simulation displayed on the PCA space with different colors. For each cluster, the representative structure is shown in a correspondingly colored panel. All the points from the HREX simulations are displayed in the background, colored in grey.

4.4 Atom-to-bead mapping and definition of bonded terms for the ligand Org27569

For the topology of the Org27569 ligand, only small and tiny beads were employed. These bead sizes are most appropriate for small, drug-like molecules without elongated aliphatic chains. Mapping decisions (reported in Figure S6) were guided by the rules formalized by Alessandri et al., ([19]) using existing Martini Database (MAD) fragments ([20]) to maintain compatibility with validated Martini 3 topology components.

In particular, the indole ring was modelled using the corresponding fragment existing in the MAD. This representation incorporates the hinge-model mapping strategy, which introduces a mass-less virtual site whose position is defined by its constructing beads. In the adopted model, four beads (1-4) describe the virtual-site hinge. The flexible linker, connecting the indole to the piperidine, was treated to preserve the structural and chemical integrity of the amide group as well as the symmetricity of the beads describing the phenyl ring. The amide functionality, in particular, was represented using a small polar bead. The piperidine group was mapped using the corresponding building block present in the MAD.

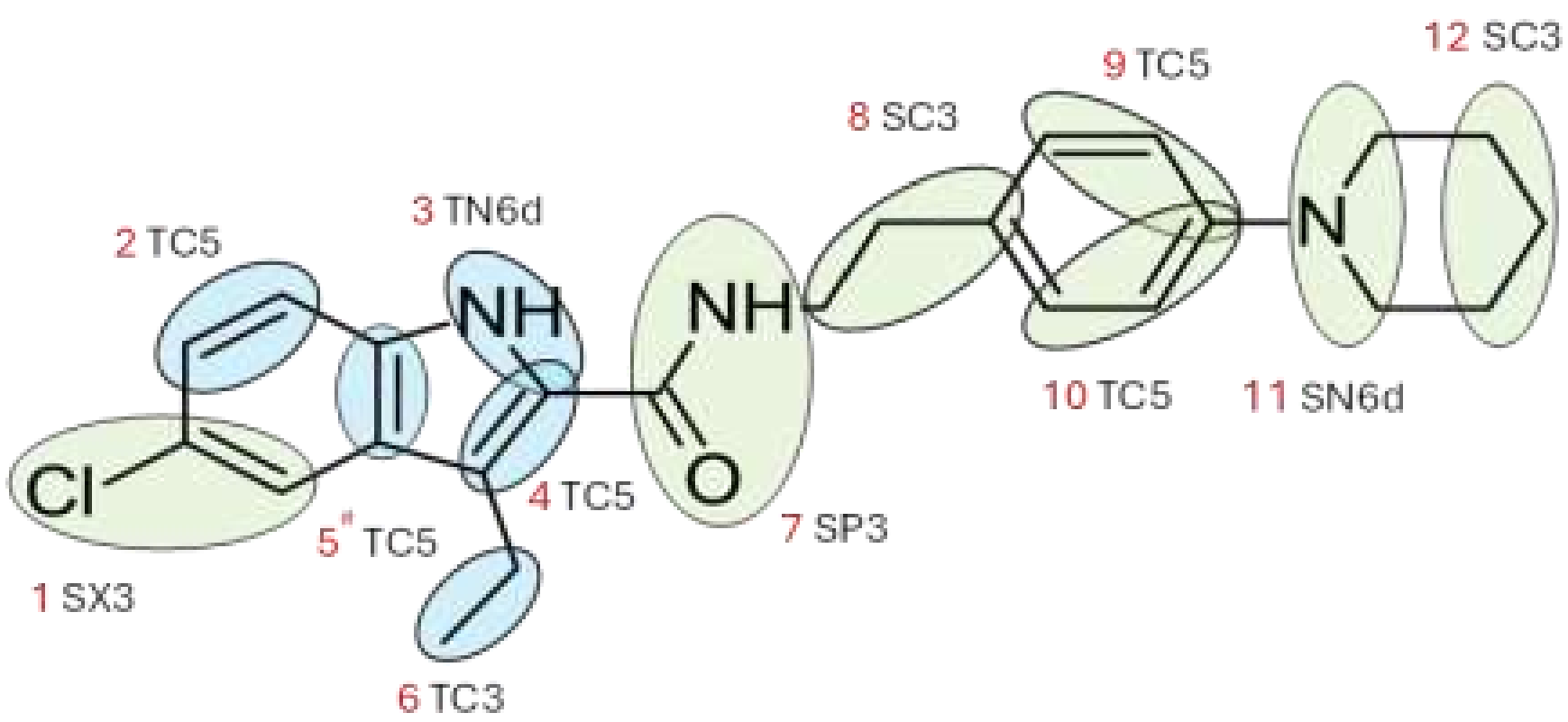

Figure S6. Atom-to-bead mapping used for Org27569. Atoms are grouped into CG beads are highlighted by ovals in different colors indicating bead size, specifically light green for *small beads*, composed of three heavy atoms, and light blue for *tiny beads*, composed of two heavy atoms. The red labels placed near each bead reports bead index, while the bead type is shown in black. The presence of a virtual site is denoted by the symbol "#".

Once the atom-to-bead mapping is established, the following step for topology construction is the definition of the bond network. Figure S7 illustrates the bond network adopted for Org27569 in the

[19] Alessandri, R.; Barnoud, J.; Gertsen, A. S.; Patmanidis, I.; de Vries, A. H.; Souza, P. C. T.; Marrink, S. J. Martini 3 Coarse-Grained Force Field: Small Molecules. *Adv. Theory Simul* **2022**, 5 (1), 2100391, DOI: 10.1002/adts.202100391

[20] MArtini Data base (MAD). Available from: https://mad.ibcp.fr/force_fields.

present work. Beads are directly bonded whenever the corresponding atoms are bonded in the atomistic representation.

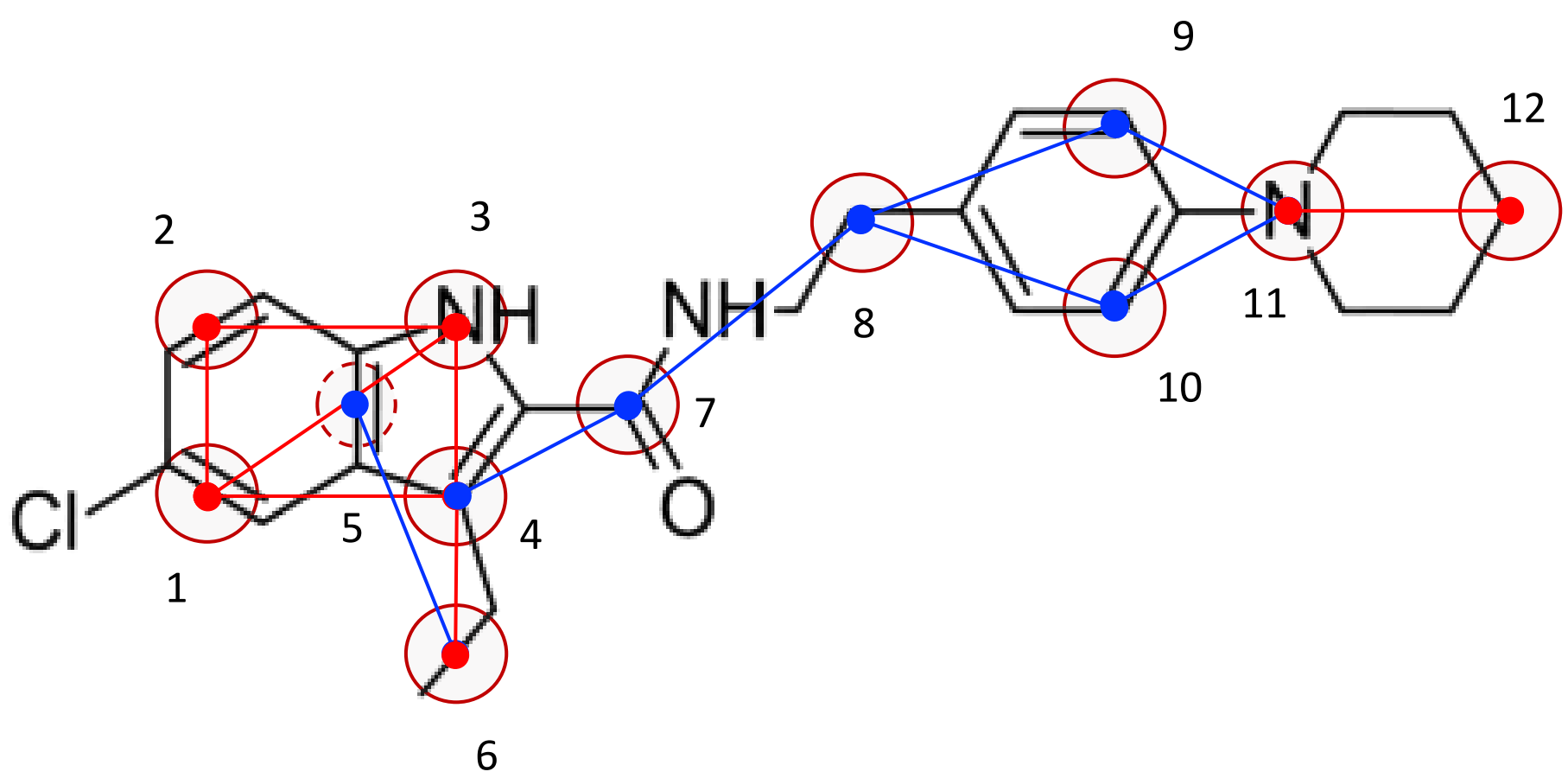

Figure S7. Schematic of the bond network. Constraints are illustrated in red, while blue lines highlight bonds modelled with harmonic potentials.

The indole moiety requires an advanced mapping strategy to yield an accurate and stable constraints network. On the one hand, its rigid and planar structure can only be preserved through the careful inclusion of constraints and improper dihedrals. On the other hand, introducing too many constraints can overload the LINCS constraint solver ([21]) and cause instabilities during the simulation. To avoid this issue, Alessandri and co-workers ([19]) proposed the so-called hinge model, which is now included in the Martini database. In this scheme, a virtual site is defined in the middle of the fused ring system, acting as a hinge for the surrounding beads. Additionally, an improper dihedral involving beads 1, 2, 3, and 4 enforces planarity, thereby preserving the flat geometry of the indole group. The resulting scheme is depicted in Figure S8.A. An alternative is to introduce a higher number of constraints (Fig. S8B); however, while theoretically valid, this scheme is more prone to numerical instabilities during MD simulations. ([19])

[21] Hess, B.; Bekker, H.; Berendsen, H. J. C.; Fraaije, J. G. E. M. LINCS: A linear constraint solver for molecular simulations. *J. Comput. Chem.* **1997**, 18, 1463– 1472, DOI: 10.1002/(SICI)1096-987X(199709)18:12<1463::AID-JCC4>3.0.CO;2-H

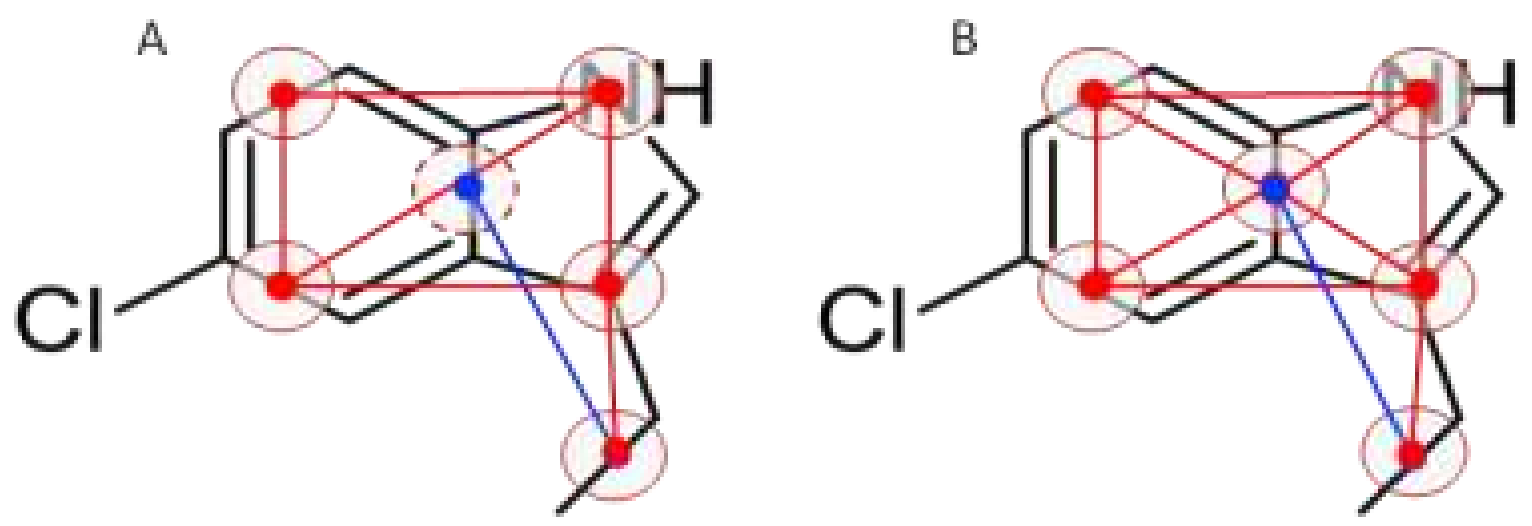

Figure S8. Bond network for the indole moiety (A) as proposed in (16) and used in this work, and (B) an alternative network without virtual site.

For this specific fragment, a set of improper dihedrals is also necessary to preserve the planarity of the aromatic moiety and to maintain the right orientation of its substituents. For instance, the improper dihedral involving the bead 1-2-3-4 enforces the flatness of the indole ring, while the improper dihedrals 6-3-2-1 and 4-6-5-3 constraint the ethyl group to the proper position relative to the indole moiety. Finally, the improper 3-7-4-5 ensures that bead number 7, representing the amide moiety of the molecule linker, remains correctly oriented. The overall scheme for bonded terms is summarized in Table S6.

Table S6. Bond terms included in the topology of Org27569. The indices refer to the beads as illustrated in Fig. S5.

Bonds	Constraints	Angles	Improper dihedrals
6-5	6-4	4-7-8	1-2-3-4
7-8	7-4	3-4-7	6-3-2-1
11-9	11-12	3-4-6	4-6-5-3
11-10	1-2	7-8-10	3-7-4-5
8-9	2-3	8-9-10	
8-10	3-4	8-10-11	
	4-1	10-11-12	
	1-3		
	9-10		

Once the bonded terms to include in the topology are defined, reference values and force constants allowing to reproduce the atomistic model behavior need to be determined. In particular, the reference values for bonds, angles, proper and improper dihedrals can be extracted from frequency histograms

obtained after mapping reference atomistic trajectories to CG. These histograms are then fitted with appropriate potential function to identify the values to insert in the CG topology. Herein, both the reference values and the force constants were extracted using the semi-automated tool Bartender. ([22]) It is worth noticing that the final ligand topology does not include proper dihedrals, which definition led to numerical instabilities causing simulation crashes. The topology parameters associated to the terms reported in Table S3 is detailed in GROMACS formatting in Tables S7-10.

[22] Pereira, G. P.Bartender: Martini 3 Bonded Terms via Quantum Mechanics-Based Molecular Dynamics. *J. Chem. Theory Comput.* **2024**, 20, 5763– 5773, DOI: 10.1021/acs.jctc.4c00275

Table S7. Bonds entries in the GROMACS formatting.

Bead index	Function	Length (nm)	Force constant ($kJ\ mol^{-1}\ nm^{-2}$)
6-5	1	0.262	21376
7-8	1	0.371	17433
11-9	1	0.258	16962
11-10	1	0.343	17174
8-9	1	0.365	13426
8-10	1	0.367	12444

Table S8. Constraints entries in the GROMACS formatting.

Bead index	Function	Length (nm)
6-4	1	0.262
7-4	1	0.244
11-12	1	0.229
1-2	1	0.297
2-3	1	0.347
3-4	1	0.164
4-1	1	0.396
1-3	1	0.456
9-10	1	0.266

Table S9. Angles entries in the GROMACS formatting.

Bead index	Function	Angle (deg°)	Force constant (kJ mol^{-1} nm^{-2})
4-7-8	1	175.25	80
3-4-7	1	88.01	1633
3-4-6	1	160.48	181
7-8-10	1	121.48	11
8-9-10	1	69.09	515
8-10-11	1	134.77	287
10-11-12	1	125.88	29

Table S10. Improper dihedrals entries in the GROMACS formatting.

Bead index	Function	Angle (deg°)	Force constant (kJ mol^{-1} nm^{-2})
1-2-3-4	2	0.00	238.41
6-3-2-1	2	10.27	187.60
4-6-5-3	2	13.72	238.07
3-7-4-5	2	0.00	49.91

4.5 CG-MD simulations of Org27569 in water

After defining the parameters for the Org27569 ligand in the CG topology file, their reliability was tested in CG simulations. An initial minimization of 10.000 steps with the steepest descent algorithm was followed by two NPT equilibration steps:

• 1 ns at 300 K using v-rescale and Berendsen barostat, ([23]) with the atomistic time step of 1 fs to preserve the simulation stability;

• 5 ns at 300 K using v-rescale and Berendsen, with the CG time step of 20 fs.

For production, we performed a 500 ns CG simulation of the ligand in water in the NPT ensemble at 300 K through the v-rescale thermostat ([15]) and the c-rescale barostat, ([16]) employing the Martini water model. The default 20 fs time step for CG simulations employing Martini 3 force field. ([24]) Hence, this simulation served as preliminary verification of parameters stability, with physiochemically realistic conditions.

4.6 Comparison of Org27569 bonded parameter distribution between HREX full-atom and CG MD simulations

Frequency histograms of the bonded terms were extracted from both atomistic HREX and CG plain MD trajectories. By assessing the agreement between these simulations, the numerical values of the bonded terms can be gradually refined. The comparison of the frequencies for the bonded terms in Org27569 from the atomistic HREX and CG plain MD trajectories is reported in Figures S9-11.

---

[23] Berendsen, H. J. C.; Postma, J. P. M.; van Gunsteren, W. F.; DiNola, A.; Haak, J. R. Molecular dynamics with coupling to an external bath. *J. Chem. Phys.* **1984**, 81, 3684– 3690, DOI: 10.1063/1.448118

[24] Souza, P. C. T.; Alessandri, R.; Barnoud, J.; Thallmair, S.; Faustino, I.; Grünewald, F.; Patmanidis, I.; Abdizadeh, H.; Bruininks, B. M. H.; Wassenaar, T. A.; Kroon, P. C.; Melcr, J.; Nieto, V.; Corradi, V.; Khan, H. M.; Domański, J.; Javanainen, M.; Martinez-Seara, H.; Reuter, N.; Best, R. B.; Vattulainen, I.; Monticelli, L.; Periole, X.; Tieleman, D. P.; de Vries, A. H.; Marrink, S. J. Martini 3: A General Purpose Force Field for Coarse-Grained Molecular Dynamics. *Nat. Methods* **2021**, 18 (4), 382– 388, DOI: 10.1038/s41592-021-01098-3

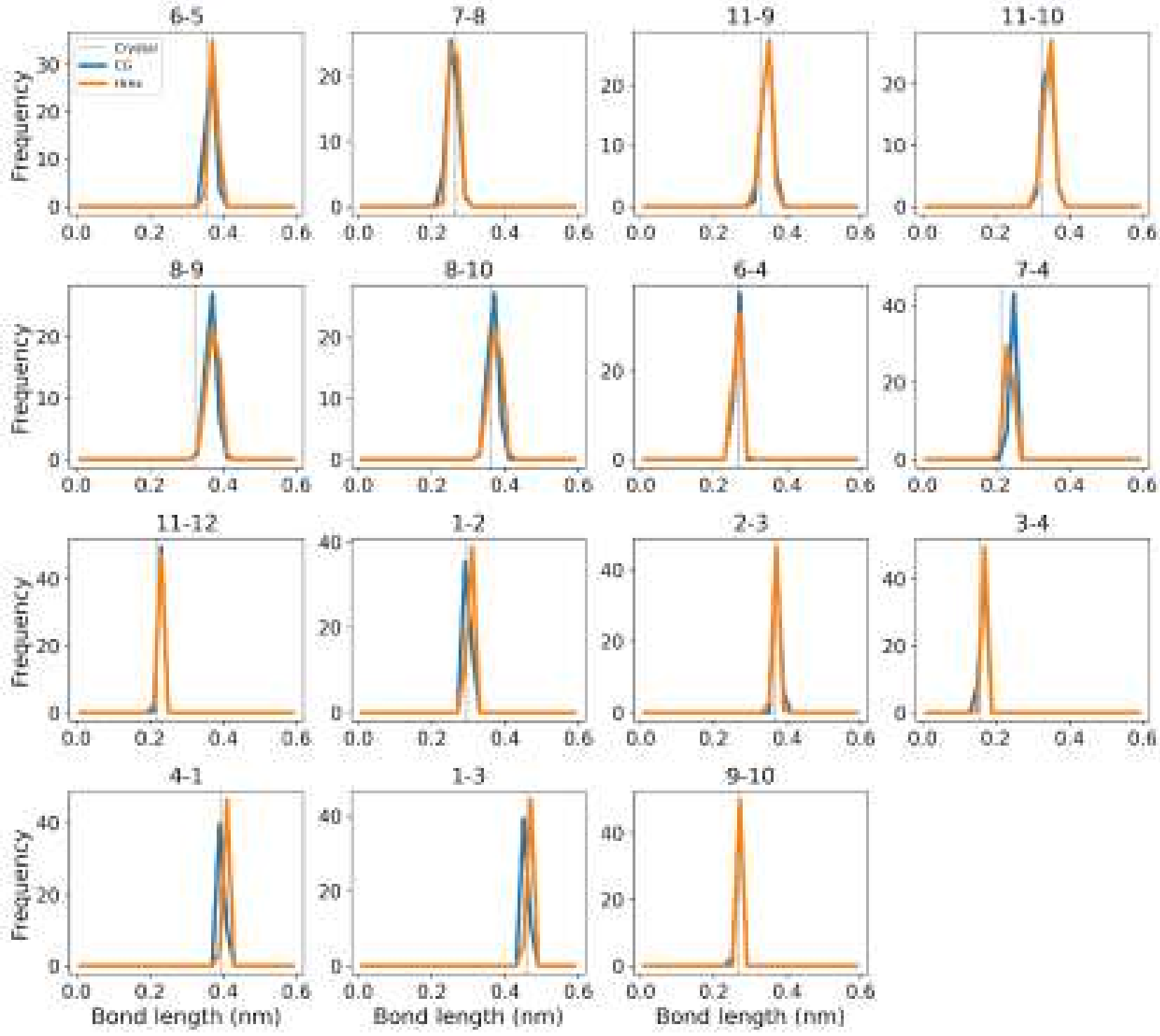

Figure S9. Frequency histograms of bonds and constraints in CG simulation (blue) and HREX atomistic simulation (orange). The dashed vertical grey line highlights the value adopted by the ligand in the crystallographic binding pose.

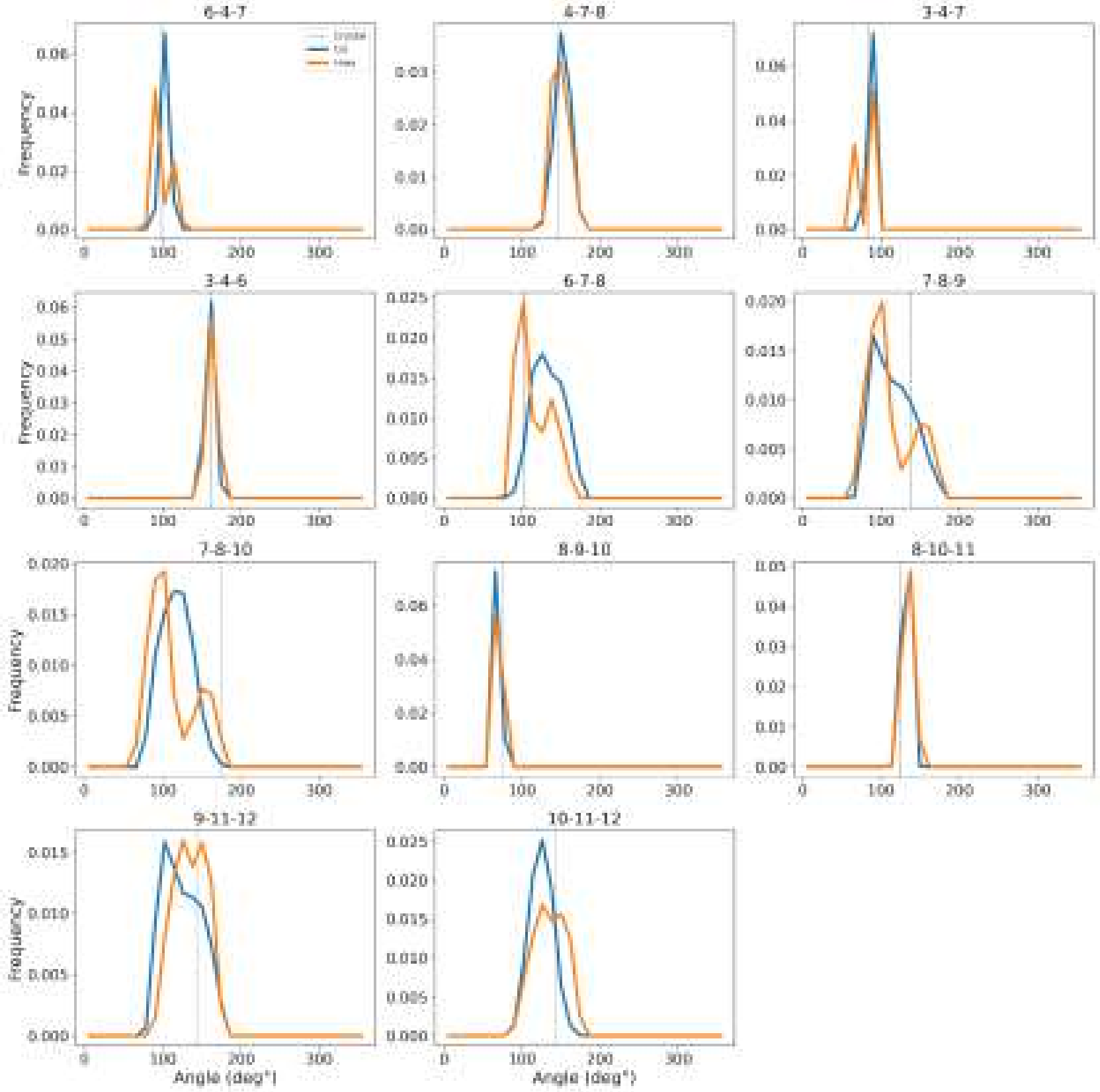

Figure S10. Frequency histograms of angles in CG simulations (blue) and HREX atomistic simulation (orange). The dashed vertical grey line highlights the value adopted by the ligand in the crystallographic binding pose.

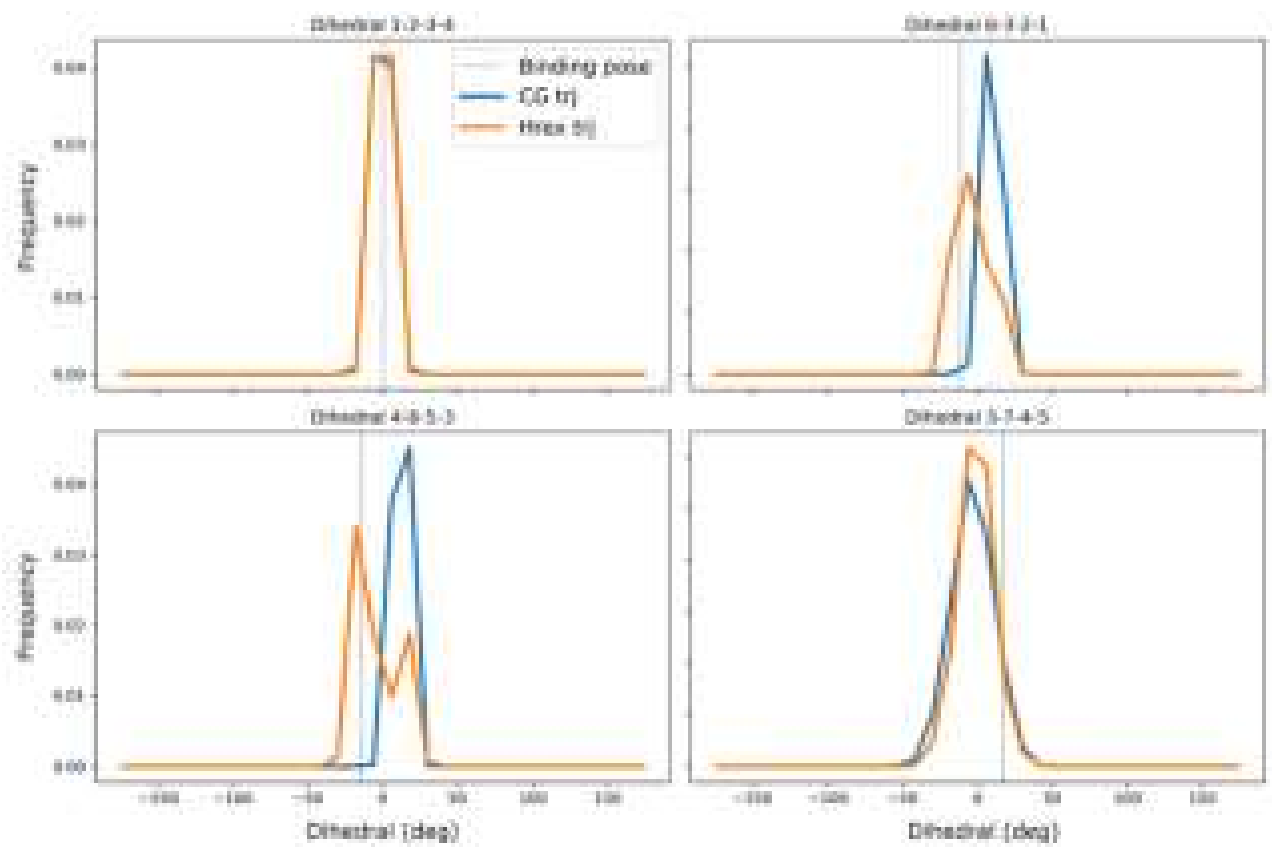

Figure S11. Frequency histograms of dihedrals in CG simulations (blue) and HREX atomistic simulation (orange). The dashed vertical grey line highlights the value adopted by the ligand in the crystallographic binding pose.

S5. Impact of trajectory striding on density map quality and pocket ranking

We evaluated the impact of trajectory striding on cholesterol density maps by generating reduced trajectories in which only every N picoseconds a frame was written (N = 100, 200, 2000, 20000, 200000) prior to density calculation. The resulting strided trajectories were analyzed by ranking NanoShaper pockets to assess whether reducing the number of frames affected pocket identification.

Visual inspection shows that increasing the stride leads to a progressive loss of density resolution: retained cholesterol density points become increasingly diffused relative to the reference protein, and their overall number decreases with larger strides (Figure S12). Despite these changes in the density maps, the ranking of the highest-scoring pockets remained unchanged across all tested strides (Table S10). In this specific case, pocket scores were dominated by statistically significant outliers, making the ranking insensitive to moderate variations in density point distribution.

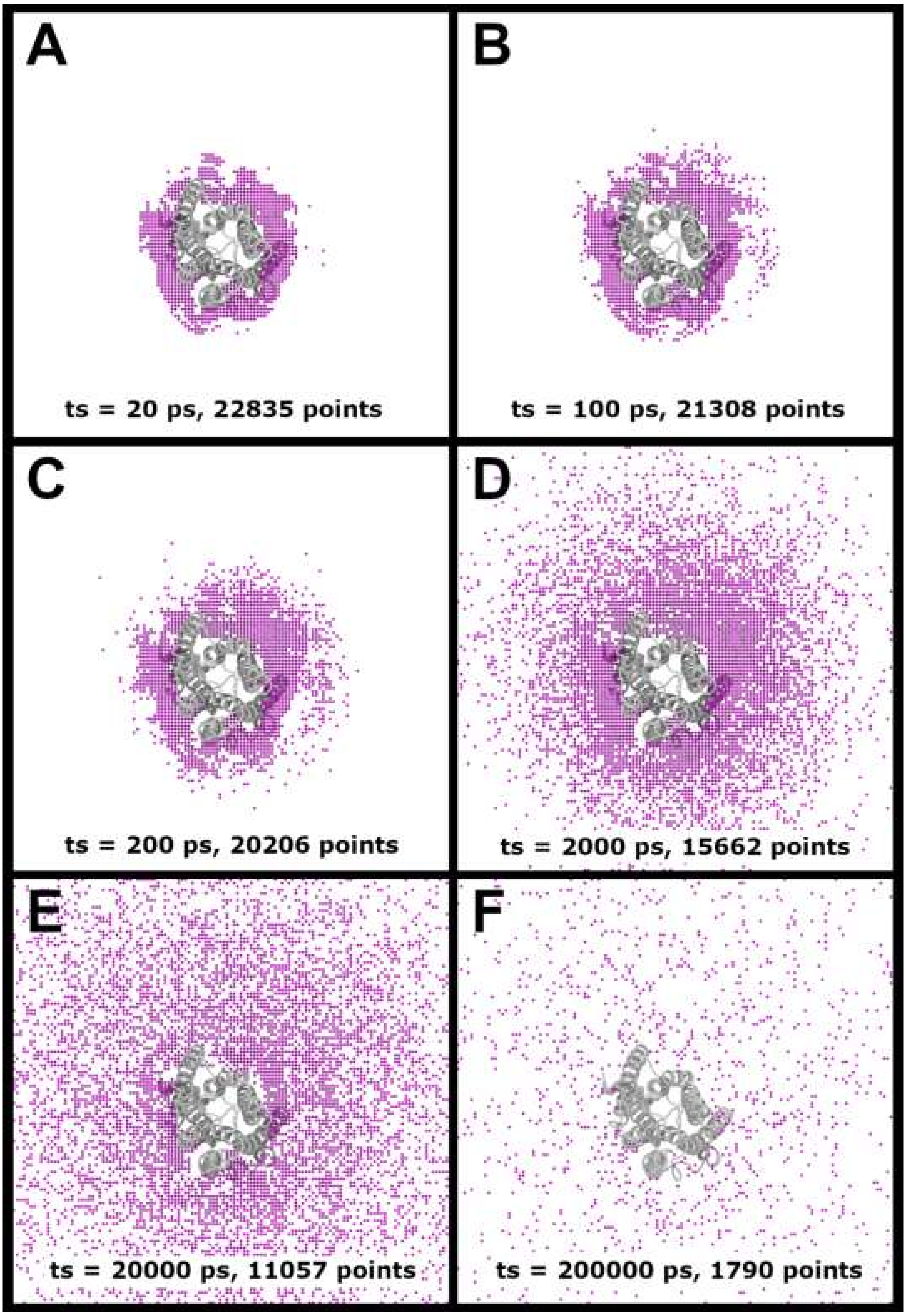

Figure S12. Relevant density (XYZ files) of the trajectories with the different introduced strides (from the default 20 ps to 200000 ps), corresponding to the panels A to E, respectively) on one of the MD replicas of the CB_1 receptor. The scatter of the density, and therefore, the real meaningfulness of the retrieved point dilutes the more stride it is introduced, stating the importance of increased sampling and the use of all frames.

However, this behavior cannot be assumed to hold universally. In systems characterized by a small number of well-defined cholesterol binding sites accompanied by weaker, more diffuse interaction regions, the precise distribution of cholesterol density points may influence pocket ranking. In such cases, reduced density resolution could alter the relative ordering of pockets with comparable scores. Among the tested options, retaining every 10th frame represented the only stride that preserved a density distribution comparable to the original trajectory, while offering a substantial reduction in storage requirements (approximately 100 GB per replica). Nevertheless, because intermediate trajectory files can be deleted after analysis, we found no strong justification for introducing striding into the default workflow. Moreover, implementing trajectory striding introduces additional technical complexity. Applying a stride via the *-skip* flag during the initial trjconv step, used to process system geometry and periodic boundary conditions, produced corrupted trajectories for small strides that failed GROMACS integrity checks. This issue could be resolved by splitting the procedure into two separate trjconv runs, one to introduce the stride and one to process geometry and periodicity, but at the cost of additional steps and runtime. As an alternative, the *-dt* flag in the GROMACS spatial tool was tested to sample frames at fixed time intervals (e.g., every 200 ps, corresponding to every 10 frames). While this approach produced density maps equivalent to those obtained from manually strided trajectories, it did not reduce the size of intermediate trajectory files and only marginally decreased the size of the final density grids (≈200 MB), at the expense of reduced spatial precision. Given the limited practical benefit, added runtime, and increased complexity, we concluded that introducing trajectory striding is not justified in the current protocol.

Table S11. Ranked NanoShaper pockets using the strided densities (strides of 20 and 100 ps has been omitted) with outlier residues bolded. In every case the order of the top five scoring pockets remains unchanged.

Stride (ps)	Rank	Pocket id	ResIDs	Contributing Points	Outlier Score	Total Score
**200**	1	11	**165$^{2.52}$** **169$^{2.56}$** **172$^{2.59}$** 190$^{3.26}$ **191$^{3.27}$** 192$^{3.28}$ 194$^{3.30}$ **195$^{3.31}$** **198$^{3.34}$** 199$^{3.35}$ **241$^{4.50}$** **245$^{4.54}$** 248$^{4.57}$ 249$^{4.58}$ 252$^{4.61}$	6000	8000	14000
	2	15	**201$^{3.37}$** 204$^{3.40}$ 205$^{3.41}$ **208$^{3.44}$** **209$^{3.45}$** **240$^{4.49}$** **243$^{4.52}$** 244$^{4.53}$ 247$^{4.56}$ 279$^{5.43}$ **282$^{5.46}$** 283$^{5.47}$ 286$^{5.50}$	3408	6000	9408
	3	12	283$^{5.47}$ 284$^{5.48}$ **287$^{5.51}$** 288$^{5.52}$ **291$^{5.55}$** 352$^{6.44}$ **353$^{6.45}$** 356$^{6.48}$ 357$^{6.49}$ 360$^{6.52}$	3875	4000	7875
	4	14	**291$^{5.55}$** 294$^{5.58}$ 295$^{5.59}$ 346$^{6.38}$ 349$^{6.41}$ **350$^{6.42}$** **353$^{6.45}$**	2430	3000	5430
	5	9	138$^{1.54}$ 141$^{1.57}$ 142$^{1.58}$ 148$^{98.83}$ 153$^{2.40}$ 154$^{2.41}$ 157$^{2.44}$ 158$^{2.45}$	337	1000	1337
**2000**	1	11	**165$^{2.52}$** **169$^{2.56}$** **172$^{2.59}$** 190$^{3.26}$ **191$^{3.27}$** 192$^{3.28}$ 194$^{3.30}$ **195$^{3.31}$** **198$^{3.34}$** 199$^{3.35}$ **241$^{4.50}$** **245$^{4.54}$** 248$^{4.57}$ 249$^{4.58}$ 252$^{4.61}$	3150	8000	11150
	2	15	**201$^{3.37}$** 204$^{3.40}$ 205$^{3.41}$ **208$^{3.44}$** **209$^{3.45}$** **240$^{4.49}$** **243$^{4.52}$** 244$^{4.53}$ 247$^{4.56}$ 279$^{5.43}$ **282$^{5.46}$** 283$^{5.47}$ 286$^{5.50}$	1824	6000	7824
	3	12	283$^{5.47}$ 284$^{5.48}$ **287$^{5.51}$** 288$^{5.52}$ **291$^{5.55}$** 352$^{6.44}$ **353$^{6.45}$** 356$^{6.48}$ 357$^{6.49}$ 360$^{6.52}$	1852	4000	5852
	4	14	**291$^{5.55}$** 294$^{5.58}$ 295$^{5.59}$ 346$^{6.38}$ 349$^{6.41}$ **350$^{6.42}$** **353$^{6.45}$**	1206	3000	4206
	5	9	138$^{1.54}$ 141$^{1.57}$ 142$^{1.58}$ 148$^{98.83}$ 153$^{2.40}$ 154$^{2.41}$ 157$^{2.44}$ 158$^{2.45}$	196	1000	1196
**20000**	1	11	**165$^{2.52}$** **169$^{2.56}$** **172$^{2.59}$** 190$^{3.26}$ **191$^{3.27}$** 192$^{3.28}$ 194$^{3.30}$ **195$^{3.31}$** **198$^{3.34}$** 199$^{3.35}$ **241$^{4.50}$** **245$^{4.54}$** 248$^{4.57}$ 249$^{4.58}$ 252$^{4.61}$	816	8000	8816
	2	15	**201$^{3.37}$** 204$^{3.40}$ 205$^{3.41}$ **208$^{3.44}$** **209$^{3.45}$** **240$^{4.49}$** **243$^{4.52}$** 244$^{4.53}$ 247$^{4.56}$ 279$^{5.43}$ **282$^{5.46}$** 283$^{5.47}$ 286$^{5.50}$	440	6000	6440
	3	12	283$^{5.47}$ 284$^{5.48}$ **287$^{5.51}$** 288$^{5.52}$ **291$^{5.55}$** 352$^{6.44}$ **353$^{6.45}$** 356$^{6.48}$ 357$^{6.49}$ 360$^{6.52}$	341	4000	4341

	4	14	**291**$^{\mathbf{5.55}}$ 294$^{5.58}$ 295$^{5.59}$ 346$^{6.38}$ 349$^{6.41}$ **350**$^{\mathbf{6.42}}$ **353**$^{\mathbf{6.45}}$	222	3000	3222
	5	9	138$^{1.54}$ 141$^{1.57}$ 142$^{1.58}$ 148$^{98.83}$ 153$^{2.40}$ 154$^{2.41}$ 157$^{2.44}$ 158$^{2.45}$	68	1000	1068
**200000**	1	11	**165**$^{\mathbf{2.52}}$ **169**$^{\mathbf{2.56}}$ **172**$^{\mathbf{2.59}}$ 190$^{3.26}$ **191**$^{\mathbf{3.27}}$ 192$^{3.28}$ 194$^{3.30}$ **195**$^{\mathbf{3.31}}$ **198**$^{\mathbf{3.34}}$ 199$^{3.35}$ **241**$^{\mathbf{4.50}}$ **245**$^{\mathbf{4.54}}$ 248$^{4.57}$ 249$^{4.58}$ 252$^{4.61}$	71	8000	8071
	2	15	**201**$^{\mathbf{3.37}}$ 204$^{3.40}$ 205$^{3.41}$ **208**$^{\mathbf{3.44}}$ **209**$^{\mathbf{3.45}}$ **240**$^{\mathbf{4.49}}$ **243**$^{\mathbf{4.52}}$ 244$^{4.53}$ 247$^{4.56}$ 279$^{5.43}$ **282**$^{\mathbf{5.46}}$ 283$^{5.47}$ 286$^{5.50}$	21	6000	6021
	3	12	283$^{5.47}$ 284$^{5.48}$ **287**$^{\mathbf{5.51}}$ 288$^{5.52}$ **291**$^{\mathbf{5.55}}$ 352$^{6.44}$ **353**$^{\mathbf{6.45}}$ 356$^{6.48}$ 357$^{6.49}$ 360$^{6.52}$	23	4000	4023
	4	14	**291**$^{\mathbf{5.55}}$ 294$^{5.58}$ 295$^{5.59}$ 346$^{6.38}$ 349$^{6.41}$ **350**$^{\mathbf{6.42}}$ **353**$^{\mathbf{6.45}}$	12	3000	3012
	5	9	138$^{1.54}$ 141$^{1.57}$ 142$^{1.58}$ 148$^{98.83}$ 153$^{2.40}$ 154$^{2.41}$ 157$^{2.44}$ 158$^{2.45}$	10	1000	1010

S6. Analysis of residue outliers over simulation time

In order to assess the effect of the simulation time on the outliers detected by the protocol, we tracked the evolution of the residues labeled as statistical outliers at different timestamps (1, 5, 10, 15, 20, 25 and 30 μs). This analysis was performed for both replicas on the simulations of the serotonin transporter (for both membrane compositions) and of the CB_1 receptor. Since each replica represents a different starting system due to the random nature of lipid positioning during the system build, the establishment of the relevant protein-cholesterol contacts can rapidly happen or be delayed depending on the starting distance of cholesterol molecules to these relevant residues. The results, as observed in Figures S13, S14 and S15 are diverse, even across the replicas of the same system. While most of the longest and persistent contacts tend to appear early, some of the less apparent but still relevant residues can remain concealed until the latest part of the simulations. As the aim of this protocol was to detect all relevant regions in cholesterol binding, we find enough justification for our extended simulation times in these late appearing statistical outliers which, despite possibly being overshadowed by more persistent residues, present the potential of meaningful cholesterol interactions. Moreover, as discussed in the main text, this method was designed to be flexible and can be easily adapted to fit specific needs with some minor modifications (e.g., if more replicas with shorter simulation times are desired).

Replica 1								
ResID	Timestamps (µs)							Longest contact (ps)
	1	5	10	15	20	25	30	
165	■	■	■	■	■	■	■	867100
241	■	■	■	■	■	■	■	663000
202		■	■	■	■	■	■	527420
245	■	■	■	■	■	■	■	499240
172	■	■	■	■	■	■	■	344700
198	■	■	■	■	■	■	■	274380
161		■	■	■	■	■	■	234120
201				■	■	■	■	213240
287	■		■	■	■	■	■	207480
240				■	■	■	■	202340
291	■		■	■	■	■	■	197760
353	■		■	■	■	■	■	197500
191	■	■	■	■	■	■	■	190060
169		■	■	■	■	■	■	137320
243	■	■		■	■	■	■	133140
282		■	■	■	■	■	■	131480
236					■	■	■	120340
168		■	■	■	■	■	■	120160
209					■	■	■	117780
360			■	■	■	■	■	115580
212					■	■	■	106180
350					■	■	■	102020
195	■					■	■	101340
232					■	■	■	97800
361			■	■	■	■	■	96720
139	■				■	■		93280
192				■	■	■		92300
208				■	■			92160
247				■				89640
124	■	■						73520
288	■							63000
354	■							55100
292	■							47180
142	■							35600
357	■							27720
135	■							27160

Replica 2								
ResID	Timestamps (µs)							Longest contact (ps)
	1	5	10	15	20	25	30	
241	■	■	■	■	■	■	■	727340
165		■	■	■	■	■	■	609020
245	■	■	■	■	■	■	■	410000
202		■	■	■	■	■	■	377980
161		■	■	■	■	■	■	299760
198		■	■	■	■	■	■	288700
240				■	■	■	■	267000
201						■	■	152200
191			■	■	■	■	■	149200
243	■	■	■	■	■	■	■	144040
282	■	■	■	■	■	■	■	129660
172	■	■	■	■	■	■	■	125240
142				■	■	■	■	118780
208	■					■	■	114120
291	■			■	■			99260
199				■	■			96720
195				■	■			96700
162				■	■			93120
351			■					69100
249			■					64940
353			■					63020
192		■	■					62880
278		■						60680
350	■	■						58920
255		■						57120
247	■							35320
139	■							31860
129	■							29340
381	■							26980
168	■							24140
135	■							23680

Figure S13. Outlier evolution on the CB_1 receptor considering multiple simulation time for the analysis.

Replica 1

ResID	Timestamps (µs)						Longest contact (ps)
	1	5	10	15	20	25	30	
256	■	■	■	■	■	■	■	265360
326	■	■	■	■	■	■	■	205000
171		■	■	■	■	■	■	186920
369	■			■	■	■	■	179560
479		■	■	■	■	■	■	161180
474		■	■	■	■	■	■	160040
170		■	■	■	■	■	■	151980
127		■	■	■	■	■	■	149000
174		■	■	■	■	■	■	125260
280		■	■	■		■	■	123380
478	■	■	■	■	■	■	■	112760
166					■	■	■	109560
514	■	■	■	■	■	■	■	109360
454					■	■	■	100500
293	■	■	■	■	■	■	■	99200
581	■			■	■		■	98560
283					■	■	■	97180
373	■	■					■	96120
584		■	■	■	■	■	■	95740
122			■	■	■		■	95720
191			■	■	■	■	■	93080
366	■		■	■			■	90740
289	■	■	■	■	■	■	■	89600
495				■	■	■	■	84920
123	■	■	■	■	■	■	■	82480
264					■	■	■	82400
363	■	■	■	■	■	■	■	81260
370	■					■	■	78860
188		■	■	■	■			73460
475				■	■			73360
374		■	■	■				71540
429		■	■	■				68280
268				■				66180
313			■					62440
251	■							42980
607	■							40920
126	■							35620
597	■							34240
546	■							33520
89	■							33140
367	■							31340
93	■							30200
158	■							28980
371	■							27400

Replica 2

ResID	Timestamps (µs)						Longest contact (ps)
	1	5	10	15	20	25	30	
474	■	■	■	■	■	■	■	422460
171		■	■	■	■	■	■	281660
369	■	■	■	■	■	■	■	272300
256	■	■	■	■	■	■	■	234620
289	■	■	■	■	■	■	■	217380
597					■	■	■	161960
122	■	■	■	■	■	■	■	149000
170		■	■	■	■	■	■	145760
283	■	■	■	■	■	■	■	144400
478	■		■	■	■	■	■	140780
127	■	■		■	■	■	■	138280
370	■	■	■	■	■	■	■	137240
280		■	■	■	■	■	■	134880
479		■	■	■	■	■	■	125680
174		■	■	■	■	■	■	119520
326	■	■	■	■	■	■	■	118520
373		■	■	■	■	■	■	117080
436		■	■	■	■	■	■	107900
265		■	■	■	■	■	■	106840
367	■	■	■	■	■	■	■	92680
126					■	■	■	91420
363	■	■	■	■	■	■	■	89440
584		■	■	■	■	■	■	88840
475	■	■				■	■	88660
268		■	■			■	■	86480
287	■	■	■	■	■	■	■	77680
93			■	■	■	■	■	77620
429	■	■	■	■	■	■	■	77500
366			■	■	■	■	■	76080
191	■		■	■	■	■	■	74520
123			■	■			■	74220
577		■	■	■	■	■	■	73720
425			■	■	■	■		71320
282				■				68980
269		■	■	■				68700
581		■	■	■				64420
187	■		■					62800
260			■					62720
476		■	■					62400
293	■		■					62200
89	■		■					60960
573		■						56580
188	■	■						55320
591		■						54820
271		■						54060
167		■						53360
163		■						52140
590	■							49000
550	■							35280
580	■							31060
586	■							30860
514	■							25020
576	■							22940
515	■							22860
553	■							22740

Figure S14. Outlier evolution on the serotonin transporter on the POPC:cholesterol membrane.

Replica 1								
ResID	Timestamps (µs)							Longest contact (ps)
	1	5	10	15	20	25	30	
171	■	■	■	■	■	■	■	460620
170		■	■	■	■	■	■	408180
474		■	■	■	■	■	■	311460
256		■	■	■	■	■	■	308740
280		■	■	■	■	■	■	211240
174		■	■	■	■	■	■	180340
478			■	■	■	■	■	168760
326			■	■	■	■	■	164860
167		■	■	■	■	■	■	154020
479	■	■	■	■	■	■	■	153720
163		■	■	■	■	■	■	130160
253					■	■	■	127940
584	■	■	■	■	■	■	■	116500
374					■	■	■	114780
127	■	■	■	■	■	■	■	106980
265				■	■	■	■	105960
269				■	■	■	■	105480
367					■	■	■	105220
597		■	■	■	■	■	■	104280
123			■		■	■	■	103700
373	■	■	■	■	■	■	■	100720
370	■	■			■	■	■	100620
495		■	■	■	■	■	■	97660
289		■	■	■	■	■	■	94720
313	■	■	■	■	■	■	■	93900
320					■	■	■	88840
514	■	■	■	■	■	■	■	88260
187					■	■		83220
260					■	■		81740
366	■	■	■	■	■			80540
581			■					74260
369	■	■	■					72100
268	■		■					67460
122	■							44520
130	■							42440
126	■							41360
536	■							40960
547	■							38980
362	■							38520
158	■							34500
377	■							31300
119	■							30980
550	■							29360
506	■							27100

Replica 2								
ResID	Timestamps (µs)							Longest contact (ps)
	1	5	10	15	20	25	30	
171	■	■	■	■	■	■	■	471460
256		■	■	■	■	■	■	292000
474		■	■	■	■	■	■	289100
170	■	■	■	■	■	■	■	274320
478		■	■	■	■	■	■	182400
127	■			■	■	■	■	181000
326	■	■	■	■	■	■	■	163300
584	■		■	■	■	■	■	159240
369	■	■	■	■	■	■	■	157700
479		■	■	■	■	■	■	147500
174	■	■	■	■	■	■	■	145680
167		■	■	■	■	■	■	140560
468			■	■	■	■	■	132560
163			■	■	■	■	■	129580
280		■	■	■	■	■	■	113740
191		■					■	110480
283	■	■	■	■	■	■	■	109280
370	■	■	■	■	■	■	■	106760
597	■				■	■	■	103280
363	■						■	100040
289	■	■	■	■	■	■	■	95900
366	■					■	■	94940
436		■	■	■	■	■	■	85260
122	■	■	■	■		■	■	84440
514	■						■	84080
373	■	■	■	■			■	83360
590					■	■	■	82660
268	■	■	■	■				75780
475	■	■	■	■				75100
126				■				73600
472			■					71960
374	■	■						59600
367	■	■						55280
581	■	■						53280
125		■						49520
377	■	■						46080
93		■						45400
265		■						44180
118	■							40800
495	■							37780
123	■							34440
119	■							33360
293	■							30880
535	■							28020
317	■							25900
269	■							22980
121	■							22900
544	■							21320
264	■							20680
282	■							20260
313	■							19720
120	■							18960
260	■							18960

Figure S15. Outlier evolution on the serotonin transporter on the ternary, raft-like membrane.

S7. Comparison of the cholesterol pockets found with the pipeline with the positions in experimental structures

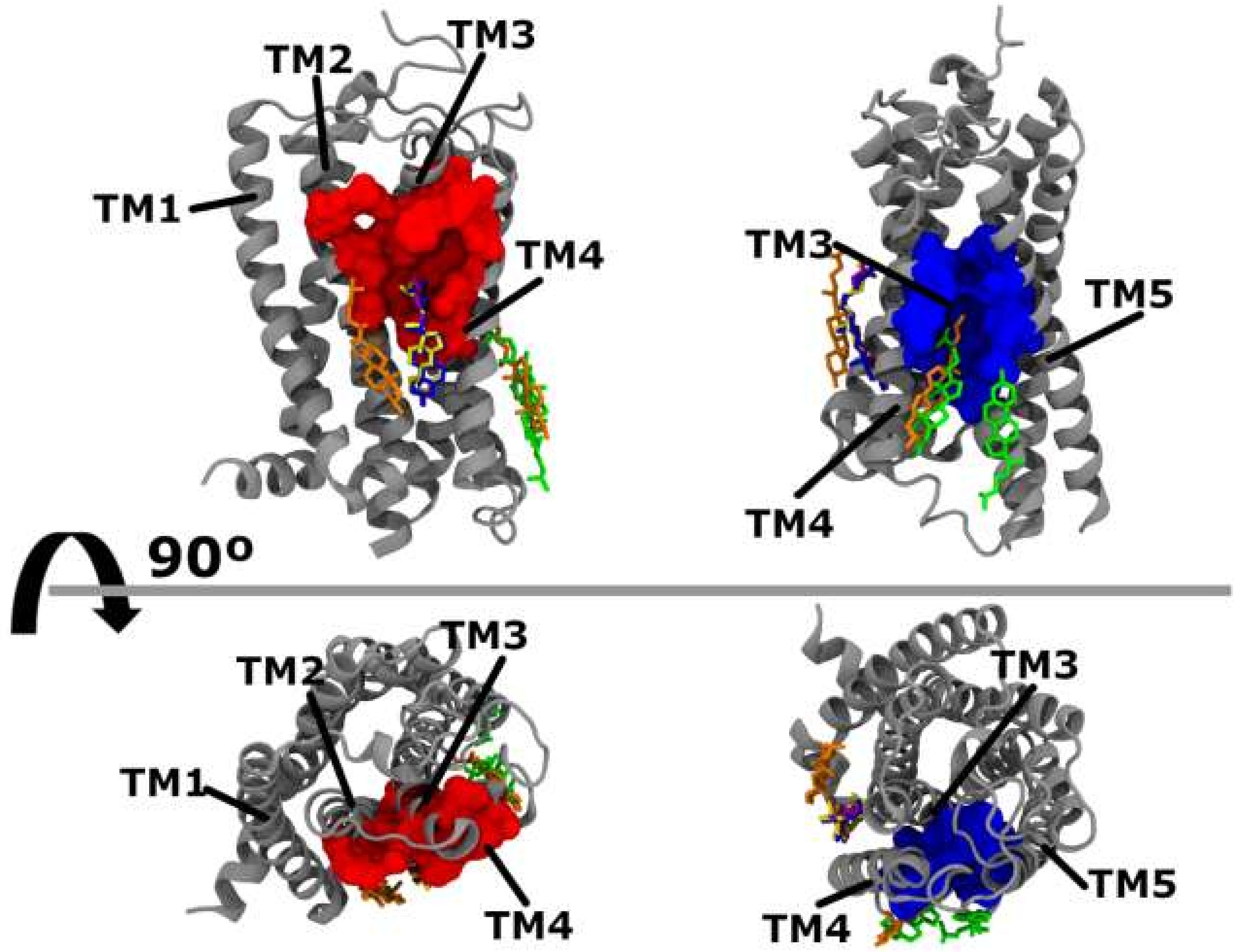

Figure S16. Overlap of every available CB1 receptor structure with bound cholesterol. Cholesterols included in the picture correspond to the structures with PDB IDs: 7FEE (orange), 5XR8 (purple), 5XRA (blue), 6NB4 (green) and 7V3Z (yellow). As shown, the cholesterol is clustered in two distinct locations, delimited by TMs 1, 2, 3 and 4 (left, colored in red) and TMs 3, 4 and 5 (right, colored in blue).

S8. Org27569 dynamics within the orthosteric binding site

To further investigate ligand behavior in relation to this site, we extracted the frames in which the ligand entered the orthosteric pocket in the three replicas where this occurred (replicas 2, 3, and 9). From these subsets, the ligand RMSD was computed both by self-alignment and by alignment on the interacting residue side chains, to assess the stability of the internal configuration within the pocket and of the interaction with the residue. Replica 2 (Figure S17) showed an average value of 2.1 and 8.0 Å for the self- and binding site–aligned RMSD, suggesting a stable conformation of the ligand that can adopt multiple conformations within the binding site. This picture is consistent across the three replicas where the interaction appears. Replica 3 (Figure S18), which displayed the longest contact within the orthosteric site, returned an average self-aligned RMSD of 2.93 Å and a side chain–aligned RMSD of 7.33 Å. Finally, replica 9 (Figure S19), which showed the shortest contact duration, had an average self-aligned RMSD of 1.90 Å and the highest side chain–aligned RMSD of 12.90 Å.

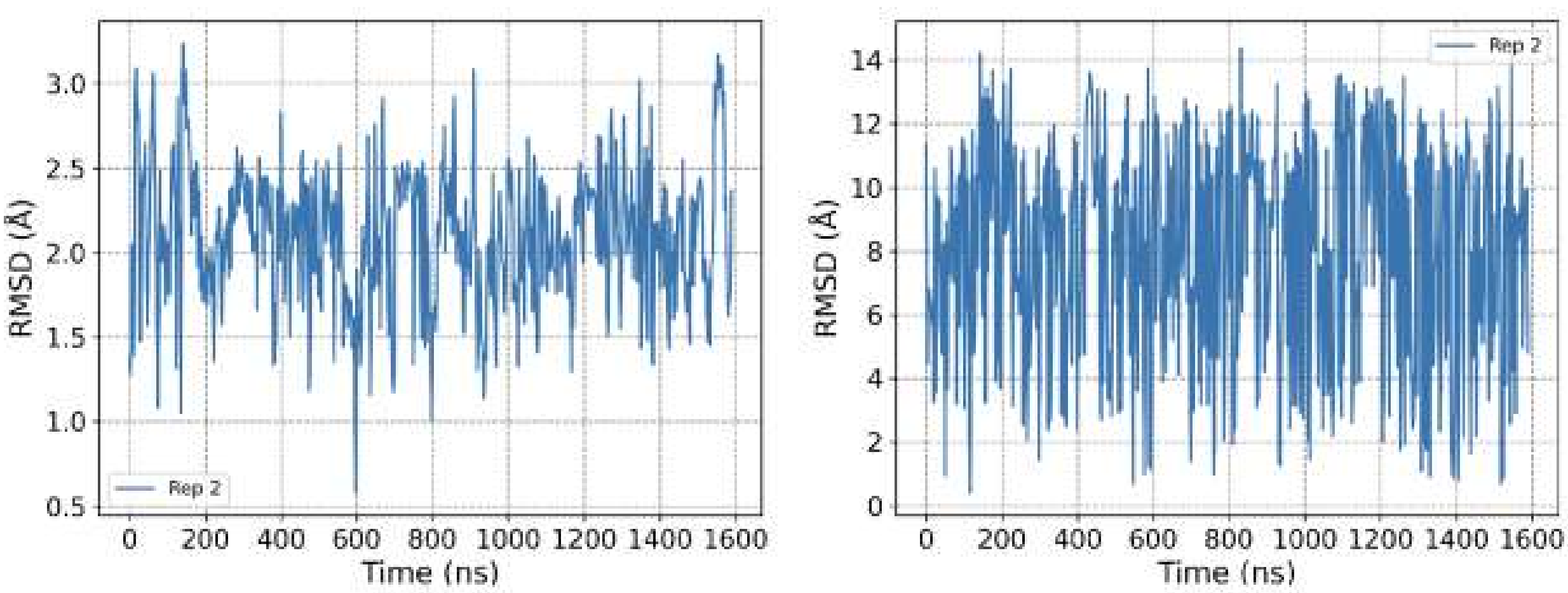

Figure S17. Replicate 2, ligand self-aligned RMSD (left) and RMSD aligned on the 379-PHE side chain (right) inside the orthosteric binding site.

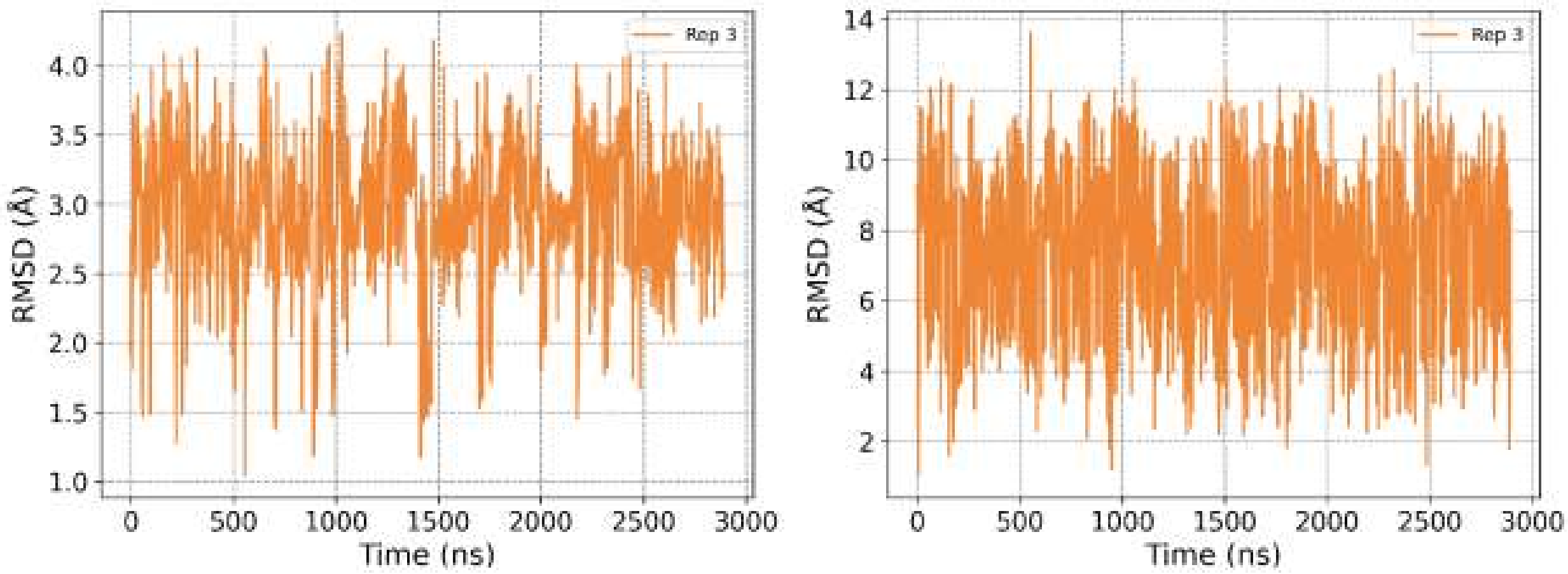

Figure S18. Replicate 3, ligand self-aligned RMSD (left) and RMSD aligned on the 379-PHE side chain (right) inside the orthosteric binding site.

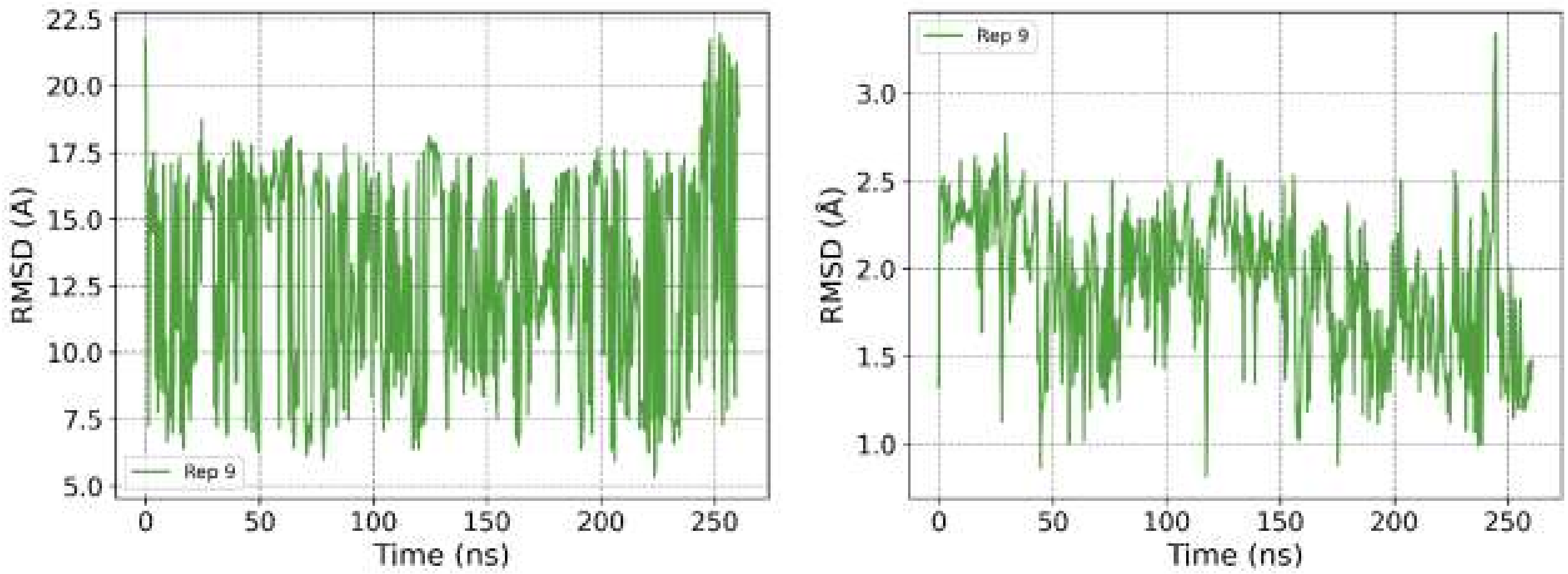

Figure S19. Replicate 9, ligand self-aligned RMSD (left) and RMSD aligned on the 379-PHE side chain (right) inside the orthosteric binding site.

S9. Org27569 contact analysis from the CG MD simulations with occupied orthosteric site

Table S12. Residues from the CB1 receptor establishing the most persistent contact in each of the 5 replicas of CG MD simulations conducted with presence of THC in the orthosteric site.

Replica Index	Main Contact	Duration (ns)
1	GLU133$^{1.49}$	309
2	TRP279$^{5.43}$	1140
3	LEU399$^{7.55}$	429
4	LEU360$^{6.52}$	722
5	VAL171$^{2.58}$	300

Table S13. Ranking of the most persistent contacts established by residues from the CB1 receptor with the Org27569 ligand in the CG MD simulations conducted with presence of THC in the orthosteric site. The top ten residues are reported, plus additional residues that displayed highest persistence in at least one of the simulations.

Residue	Frequency	Average Duration (ns)	Shortest duration (ns)	Longest duration (ns)
GLU133$^{1.49}$	5	340	236	417
LEU399$^{7.55}$	5	319	139	429
ILE395$^{7.51}$	5	276	228	309
TRP241$^{4.50}$	5	240	172	333
LEU136$^{1.52}$	5	215	127	308
VAL161$^{2.48}$	5	214	133	424
PHE237$^{4.46}$	5	204	124	366
PRO394$^{7.50}$	5	150	131	170
PHE408$^{8.54}$	5	145	109	202
TYR172$^{2.59}$	4	180	127	212
LEU360$^{6.52}$	2	473	174	772
TRP279$^{5.43}$	1	1140	1140	1140
VAL171$^{2.58}$	1	300	300	300

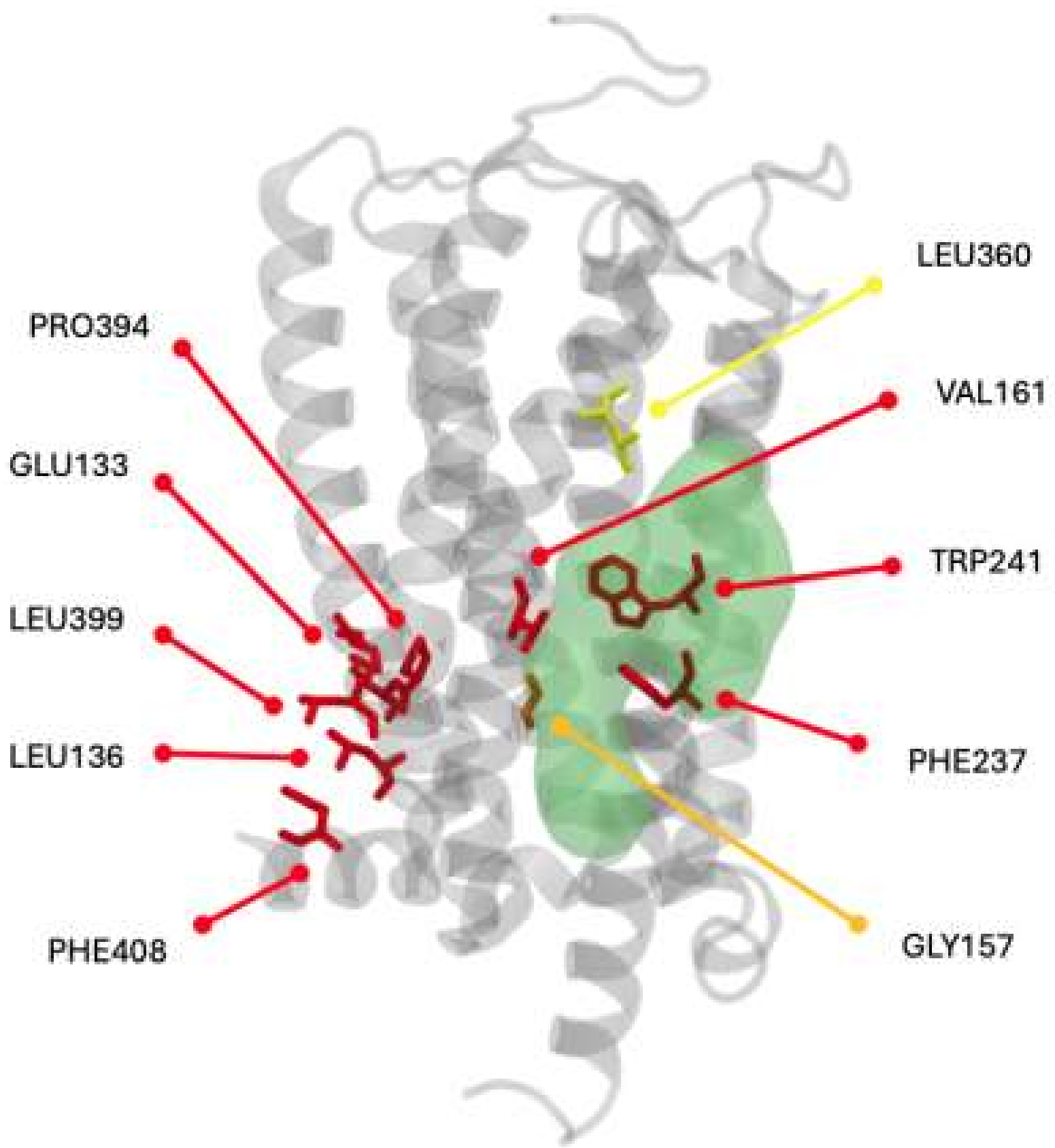

Figure S20. CB_1 receptor residues involved in the most persistent contacts with the allosteric modulator Org27569. The residues are labelled and highlighted in licorice on the cartoon representation of the receptor. Residue color scale ranging from yellow to red indicates increasing persistence of the contact. To orient the reader, the allosteric binding site is highlighted as green transparent surface.
