# Supplementary material for: Cholesterol Hot Spot Automated Mapping Protocol (CHAMP): Identifying Cholesterol-Binding Hot Spots in Membrane Proteins
Source: J Chem Inf Model. 2026 Sep 23;66(19):12667–81. doi: 10.1021/acs.jcim.6c01419 (PMC13693706; doi:10.1021/acs.jcim.6c01419)
Supplement: Supplementary file 2 [file ci6c01419_si_002.pdf]

SUPPORTING INFORMATION FILE 2

# *Cholesterol Hot spot Automated Mapping Protocol: Identifying Cholesterol-Binding Hot Spots in Membrane Proteins*

*David Sotillo-Núñez[1], Matteo Nardi Cesarini[1], Gian Marco Elisi[1], Mattia Bernetti[1], Giovanni Bottegoni[1,2*]*

[1]Dipartimento di Scienze Biomolecolari, Università degli Studi di Urbino Carlo Bo, Via Ca' Le Suore, 2/4, 61029 Urbino, Italy.

[2]School of Pharmacy, University of Birmingham, Edgbaston, B15 2TT, Birmingham, United Kingdom.

[*] Corresponding author.

Email address: giovanni.bottegoni@uniurb.it

# CHAMP usage: step-by-step tutorial

The Cholesterol Hot spot Automated Mapping Protocol (CHAMP) is designed as an end-to-end automated workflow that starts from a protein structure in the standard PDB format and performs the complete process of system preparation, membrane construction, quality assessment of the generated bilayer, simulation setup, execution of the MD simulations, and hotspot identification. CHAMP automatizes the entire procedure and minimizes manual intervention to facilitate standardized and reproducible analyses across multiple membrane proteins and membrane compositions.

CHAMP can be used in two complementary modes: i) the standard, fully-automated mode, in which the entire workflow is executed starting from a PDB structure with minimal user intervention, and ii) the modular mode, intended for advanced users, in which each stage of the workflow can be executed independently, allowing users to inspect, modify, or replace intermediate files (e.g., mdp or itp files) and customize simulation settings before proceeding to the subsequent steps. The two modes of operation are described below. Since some of the dependencies used by CHAMP might require specific or older versions of software that may already be installed in some machines, we also provide with a .yml file, available in the `tutorial_and_outputs` of this repository, which can be used by **conda** to create a working environment.

```
$ conda env create -f CHAMP.yml
```

By running the previous command conda will create a clean environment with the minimum requirements to run CHAMP. **NOTE:** You will still need to manually install GROMACS with GPU acceleration support (CUDA).

If you encounter any issues when installing or running CHAMP, do not hesitate to contact us at camd@uniurb.it. We will be happy to provide assistance and help troubleshoot any problems.

# Standard execution

### Pre-requisites and inputs

For this example tutorial we will work with the cannabinoid 1 receptor, using the the PDB structure 7FEE. We will use the pre-oriented structure provided in the OPM (Orientations of Proteins in Membranes) database, and the GPCRdb structure for the residue annotation. Thus, let's start by downloading:

- the OPM structure [1]: you will get the 7fee.pdb file
- the GPCRdb structure [2]: you will get the pdb_structures.zip file

For the structure pre-processing we will make use of any text editor, as well as the PDBFixer python package; for the latter, visit the link with installation instructions via the Conda package manager.

## 1. System preparation

Start by creating a directory to work on and move the downloaded input structures (OPM and GPCRdb) inside of it:

```
$ mkdir CHAMP_tutorial
$ cd CHAMP_tutorial
$ cp ~/Downloads/7fee.pdb 7fee_opm.pdb
$ unzip ~/Downloads/pdb_structures.zip
$ cp ~/Downloads/cnr1_human_7FEE.pdb 7fee_gpcrdb.pdb
```

The first step for cleaning up the oriented structure is to remove any non-atom components, such as heteroatom lines (HETATM) and the DUM residues used by OPM to represent membrane boundaries. To do so, you can use your preferred method; the easiest way is to open the PDB with a text editor (e.g. vim) and manually remove said lines. This specific PDB structure also contains the T4 lysozyme fusion protein inserted in the intracellular loop 3 (ICL3), which we will also remove. Fortunately, as shown below, authors annotated the atoms corresponding to the T4L in a very evident manner, but this can prove more challenging for other structures and the clean-up might require more investigation. Make sure to save the changes or store them in a new file. In this case we are saving the changes in `7fee_opm.pdb`.

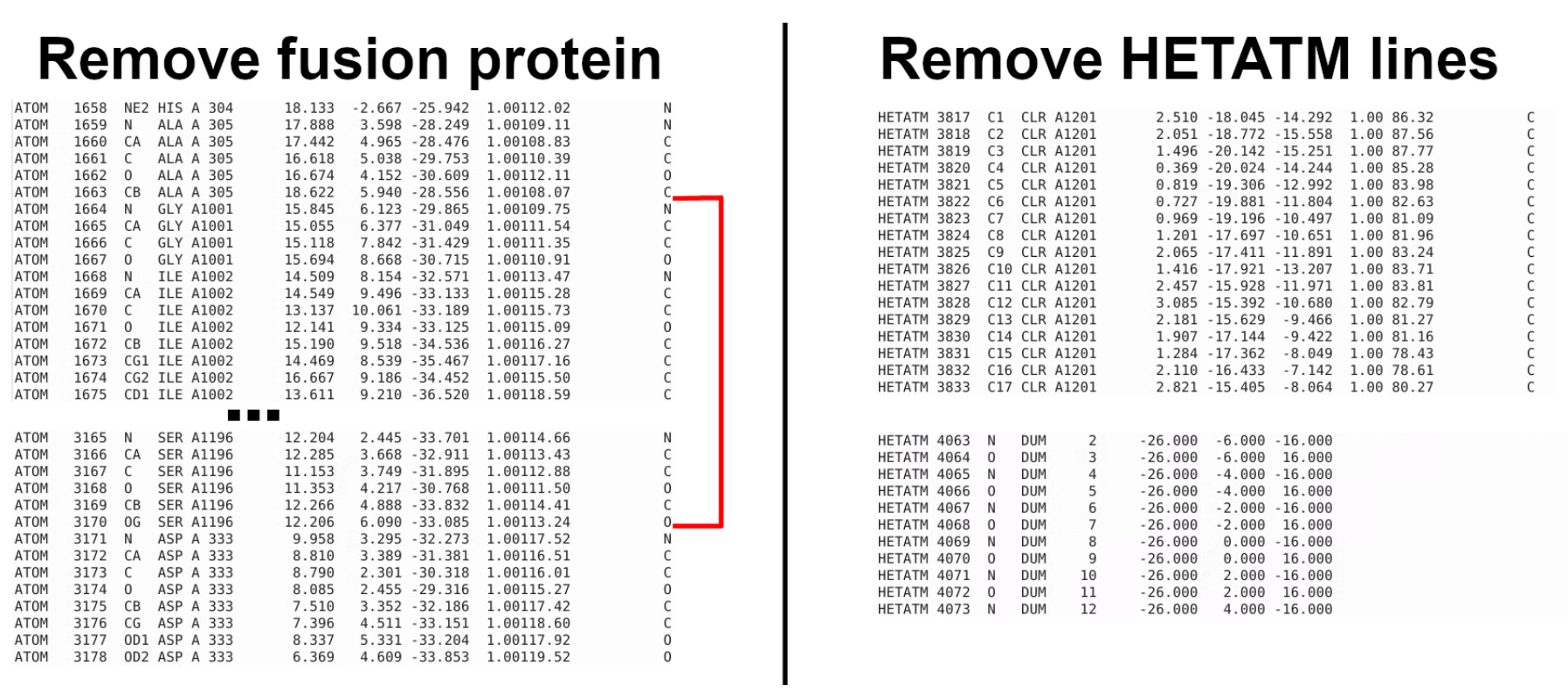


Figure 1: PDB pre-processing

For your reference, the image above shows what we removed from the example PDB used in this tutorial. The changes were then saved to `7fee_clean.pdb`.

Once the PDB file is clean of non-necessary atoms we can run PDBFixer [3] to fix incomplete residues and/or other inconsistencies in the structure:

```
$ pdbfixer 7fee_clean.pdb --output=7fee_fixed.pdb
```

The structure input and all intermediates mentioned in the previous steps are available for download in the tutorial_inputs directory.

**NOTE:** a standard run with default parameters was sufficient for this structure. If using a different system you encounter issues in subsequent steps, especially during the CG conversion by *martinize2*, it might be indicative that some non-standard atoms could be affecting the conversion, and other PDBFixer options or other forms of manual curation might be necessary.

### 2. Creating a parameters file and launching CHAMP

Once the input structure is prepared, to run CHAMP we need a GROMACS installation (preferably versions >2020) **with CUDA support for GPU acceleration** and the parameters file from which CHAMP will read and apply its settings. You can find a full list of the available parameters with their possible options in the documentation at the front page of CHAMP's repository, but for this tutorial you can use the following:

```
run_mode = full
pdb_id = 7fee
starting_structure = /path/to/fixed/pdb/7fee_fixed.pdb
n_reps = 2
mb_comp = POPC:75,CHOL:25
martinize2_path = martinize2
gmx_path = gmx_mpi
gpu = 0
gpcrdb_file_path = /path/to/gpcrdb/pdb/7fee_gpcrdb.pdb
bw_notation = True
output_dir = ./7fee
n_cores = 16
```

Write down these parameters inside a text file, and save it as 7fee.prm.

**NOTE:** always adjust with the correct paths where your files and executables are, as well as the number of cores to use in your simulations and the GPU ID where it will run. We highly encourage the use of **absolute paths** for inputs to avoid issues when managing files.

You can now proceed with the full run of CHAMP, which will execute all the steps explained in the previous sections, from start to finish and produce results equivalent to those provided in the example run, found in the corresponding directory of this repository. Execute the following command to launch the full run of CHAMP:

```
$ CHAMP 7fee.prm
```

# Modular execution

While the great advantage of CHAMP is its ability to provide results in an automatic fashion by running the full protocol from the starting inputs, we also provide with the option of a modular run, allowing to repeat or customize

specific steps if necessary, or to perform the sole analysis stages on already generated trajectories.

The pipeline assumes specific naming conventions for the files. Consequently, in order to use custom trajectories the user must adapt file names and directory structure to match the standard CHAMP conventions.

An example of how working directories are organized and the format naming that CHAMP expects can be found by referring to the `example_outputs` directory of this same repository. For convenience, we also reproduce the complete directory tree generated by CHAMP below:

```
[PDB_ID]/
└── sims/
    ├── [PDB_ID]_cg_aligned.pdb
    ├── [PDB_ID]_martinized.pdb
    ├── [PDB_ID]_POPC_75_CHOL_25_density_MERGE.xyz
    ├── [PDB_ID]_POPC_75_CHOL_25_dens_per_pocket/
    │   ├── pocket_10.xyz
    │   ├── pocket_13.xyz
    │   ├── pocket_14.xyz
    │   ├── pocket_15.xyz
    │   ├── pocket_8.xyz
    │   └── pocket_9.xyz
    ├── [PDB_ID]_POPC_75_CHOL_25_lf.pdb
    ├── [PDB_ID]_POPC_75_CHOL_25_outlier_density.pdb
    ├── [PDB_ID]_POPC_75_CHOL_25_outliers_MERGE.csv
    ├── [PDB_ID]_POPC_75_CHOL_25_ranked_pockets.csv
    ├── [PDB_ID].top
    ├── mdp_files/
    │   ├── em.mdp
    │   ├── npt_const.mdp
    │   ├── npt_noconst_1fs.mdp
    │   ├── npt_noconst_20fs.mdp
    │   ├── npt_prod.mdp
    │   └── nvt.mdp
    ├── molecule_0.itp
    ├── rep_1/
    │   ├── [PDB_ID]_POPC_75_CHOL_25_bad.top
    │   ├── [PDB_ID]_POPC_75_CHOL_25_em.edr
    │   ├── [PDB_ID]_POPC_75_CHOL_25_em.gro
    │   ├── [PDB_ID]_POPC_75_CHOL_25_em.log
    │   ├── [PDB_ID]_POPC_75_CHOL_25_em.tpr
    │   ├── [PDB_ID]_POPC_75_CHOL_25_em.trr
    │   ├── [PDB_ID]_POPC_75_CHOL_25.gro
    │   ├── [PDB_ID]_POPC_75_CHOL_25.ndx
    │   ├── [PDB_ID]_POPC_75_CHOL_25_npt_const.{cpt,edr,gro,log,tpr,xtc}
```

```
│   ├── [PDB_ID]_POPC_75_CHOL_25_npt_const_prev.cpt
│   ├── [PDB_ID]_POPC_75_CHOL_25_npt_noconst_1fs.{cpt,edr,gro,log,tpr,xtc}
│   ├── [PDB_ID]_POPC_75_CHOL_25_npt_noconst_20fs.{cpt,edr,gro,log,tpr,xtc}
│   ├── [PDB_ID]_POPC_75_CHOL_25_nvt.{cpt,edr,gro,log,tpr,xtc}
│   ├── [PDB_ID]_POPC_75_CHOL_25_prod.{cpt,edr,gro,log,tpr,xtc}
│   ├── [PDB_ID]_POPC_75_CHOL_25_prod_prev.cpt
│   ├── [PDB_ID]_POPC_75_CHOL_25.top
│   ├── analysis/
│   │   ├── [PDB_ID]_POPC_75_CHOL_25_all.xyz
│   │   ├── [PDB_ID]_POPC_75_CHOL_25_prod_sdf_1.xtc
│   │   ├── [PDB_ID]_POPC_75_CHOL_25_prod_sdf_2.xtc
│   │   ├── [PDB_ID]_POPC_75_CHOL_25_res_max_contacts.csv
│   │   ├── [PDB_ID]_POPC_75_CHOL_25_res_outliers.csv
│   │   ├── [PDB_ID]_POPC_75_CHOL_25_res_total_contacts.txt
│   │   ├── coords_tl.txt
│   │   └── grid.cube
│   ├── mdout.mdp
│   ├── membrane_check/
│   │   ├── density_profiles.png
│   │   └── membrane_parameters.info
│   └── molecule_0.itp
├── rep_2/
│   ├── [PDB_ID]_POPC_75_CHOL_25_bad.top
│   ├── [PDB_ID]_POPC_75_CHOL_25_em.edr
│   ├── [PDB_ID]_POPC_75_CHOL_25_em.gro
│   ├── [PDB_ID]_POPC_75_CHOL_25_em.log
│   ├── [PDB_ID]_POPC_75_CHOL_25_em.tpr
│   ├── [PDB_ID]_POPC_75_CHOL_25_em.trr
│   ├── [PDB_ID]_POPC_75_CHOL_25.gro
│   ├── [PDB_ID]_POPC_75_CHOL_25.ndx
│   ├── [PDB_ID]_POPC_75_CHOL_25_npt_const.{cpt,edr,gro,log,tpr,xtc}
│   ├── [PDB_ID]_POPC_75_CHOL_25_npt_const_prev.cpt
│   ├── [PDB_ID]_POPC_75_CHOL_25_npt_noconst_1fs.{cpt,edr,gro,log,tpr,xtc}
│   ├── [PDB_ID]_POPC_75_CHOL_25_npt_noconst_1fs_prev.cpt
│   ├── [PDB_ID]_POPC_75_CHOL_25_npt_noconst_20fs.{cpt,edr,gro,log,tpr,xtc}
│   ├── [PDB_ID]_POPC_75_CHOL_25_nvt.{cpt,edr,gro,log,tpr,xtc}
│   ├── [PDB_ID]_POPC_75_CHOL_25_prod.{cpt,edr,gro,log,tpr,xtc}
│   ├── [PDB_ID]_POPC_75_CHOL_25_prod_prev.cpt
│   ├── [PDB_ID]_POPC_75_CHOL_25.top
│   ├── analysis/
│   │   ├── [PDB_ID]_POPC_75_CHOL_25_all.xyz
│   │   ├── [PDB_ID]_POPC_75_CHOL_25_prod_sdf_1.xtc
│   │   ├── [PDB_ID]_POPC_75_CHOL_25_prod_sdf_2.xtc
│   │   ├── [PDB_ID]_POPC_75_CHOL_25_res_max_contacts.csv
│   │   ├── [PDB_ID]_POPC_75_CHOL_25_res_outliers.csv
│   │   ├── [PDB_ID]_POPC_75_CHOL_25_res_total_contacts.txt
```

```
│   │   ├── coords_tl.txt
│   │   └── grid.cube
│   ├── mdout.mdp
│   ├── membrane_check/
│   │   ├── density_profiles.png
│   │   └── membrane_parameters.info
│   └── molecule_0.itp
└── toppar/
    ├── martini_v3.0.0.itp
    ├── martini_v3.0.0_ions_v1.itp
    ├── martini_v3.0.0_phospholipids_v1.itp
    ├── martini_v3.0.0_solvents_v1.itp
    └── martini_v3.0_sterols_v1.0.itp
```

We will not go over how to run all the individual steps in this section, but we will remark some relevant points that can be useful if running standalone steps of CHAMP. Note that this is aimed to more advanced users and knowledge of MD simulations is encouraged, as it involved modifying parameters related to how the MD simulation behaves (length, timestep, writing frequency, etc).

CHAMP does not directly support manually setting different parameters for the MD simulations, as the protocol was carefully tailored to properly work with membrane proteins out of the box. However, some advanced users might have some trajectories available that they want analyzed that were produced with different settings or want to run the MD step by altering specific parameters. In the following (A) and (B) sections, we address both scenarios.

### (A) Manually modifying the MD parameters

The key parameters that might affect the result are the length of the simulation, the timestep and the output writing frequency.

- **Simulation length**: considering that our analysis skips the first 500 ns of the simulation to make sure that it is analysing an already valid, fully equilibrated ensemble of the system, the total length of the trajectory should be sufficient enough so that the 500 ns truncation does not affect the sampling in a relevant way.
- **Timestep**: the recommended timestep for Martini simulations is usually 20 fs [4]; however, longer or shorter timesteps might be used as well.
- **Trajectory writing frequency**: the key parameter is the time interval between trajectory frames. Users might opt to write frames less frequently. Our standard recommendation is to write trajectories every 1000 integration steps; considering the standard 20 fs MD timestep, this leads to a 20 ps interval between frames. To control this, we included the optional parameter `frame_timestep`. If the .mdp files are modified in such a way that the time between frames does not match the default 20 ps, the user

**MUST** provide it using this flag in the parameter file **in picoseconds** depending of the settings used. The way to calculate the time between frames is the following:

*Frame window* = *timestep* (**dt**) * *writing_frequency* (**nstxout-compressed**)

Example:

Frame window = 0.02 ps (20 fs) * 1000 = 20 ps

More advanced users might decide to run the MD step of the protocol but customizing the settings of the simulation. The proper way to do so is to run the `system_build` step, which will create all the necessary inputs for the MD in the working directory (system, topology files, mdp files). To do so, use a parameter file set up for that specific step. Following in the previous example:

```
run_mode = standalone
step = system_build
pdb_id = 7fee
starting_structure = /path_to_fixed_pdb/7fee_fixed.pdb
n_reps = 2
mb_comp = POPC:75,CHOL:25
martinize2_path = martinize2
gmx_path = gmx_mpi
gpu = 0
gpcrdb_file_path = /path_to_gpcrdb_pdb/7fee_gpcrdb.pdb
bw_notation = True
output_dir = ./7fee
n_cores = 16
```

which can be saved as `7fee_system_build.prm` and used for the preparation:

```
$ CHAMP 7fee_system_build.prm
```

Once the inputs are in place, the user can choose to access them and modify as desired. Some examples of this are:

- **Extending/shortening steps of the MD protocol**: Simply access the working directory (following the example above: `7fee/sims/mdp_files/`) and modify the `nsteps` parameter (simulation time is calculated by `nsteps * dt`).
- **Modifying timestep and/or writing frequency**: Access mdp files and modify `dt` and/or `nstxout-compressed`.
- **Modifying the elastic network**: This is a critical setting that will directly affect the interactions between the beads (atoms) of the system, so it should be carefully manipulated, as altering it might cause the system to break. That said, these modifications can be achieved by accessing the

protein topology, available in `7fee/sims/molecule_0.itp`. Two examples of what can be done are:

- **Altering the force of the "rubber band" bond between the atoms**: The default force for the elastic network pairs is 700 kJ/mol/nm^2, but this value can be modified for any atom pair (see the selected text in the image below).

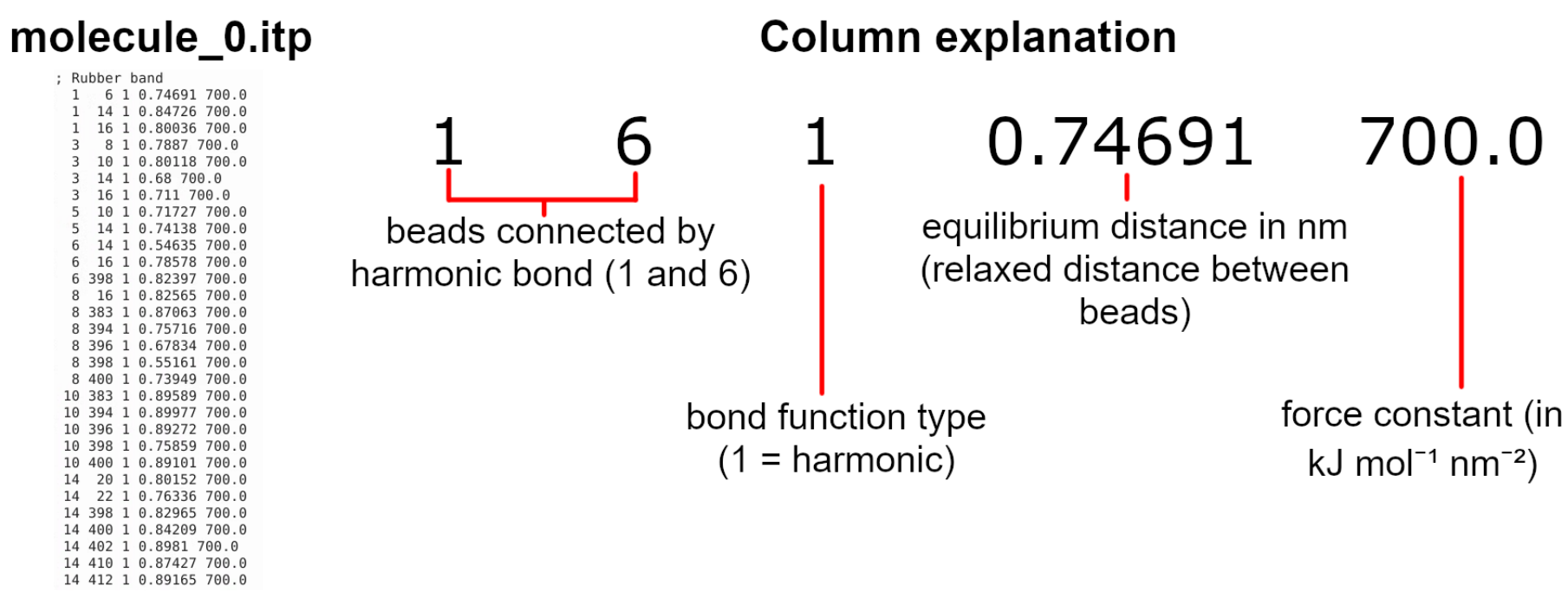


Figure 2: Altering the Elastic Network

- **Partially releasing the elastic network**: Since the elastic network in Martini representations limits side chain movement and protein conformation changes, some users might decide to release or partially relax the force of elastic bonds between certain pairs of atoms to allow movement in a specific part of the protein. This can be achieved in a similar way as before by individually modifying the values for the desired pairs of atoms rather than bulk changing the value for all.

After the desired changes on the files are set and saved, the MD step can be run as usual, either modifying the previous parameter file by updating the step to run or by creating a new one:

```
run_mode = standalone
step = MD_simulation
pdb_id = 7fee
starting_structure = /path/to/fixed/pdb/7fee_fixed.pdb
n_reps = 2
mb_comp = POPC:75,CHOL:25
martinize2_path = martinize2
gmx_path = gmx_mpi
gpu = 0
gpcrdb_file_path = /path/to/gpcrdb/pdb/7fee_gpcrdb.pdb
bw_notation = True
output_dir = ./7fee
```

```
n_cores = 16
```

We saved the previous parameter file as `7fee_MD.prm`, which can now be used to run the simulation:

```
$ CHAMP 7fee_MD.prm
```

What follows is the analysis step. However, as in the previous situation, the same considerations remain true: the length of the production run of the simulation has to be properly considered and the time between frames used in the MD simulation has to be provided via the `frame_timestep` parameter for the analysis step. An example of how that would look like in our example is the following:

```
run_mode = standalone
step = analysis
pdb_id = 7fee
starting_structure = /path/to/fixed/pdb/7fee_fixed.pdb
n_reps = 2
mb_comp = POPC:75,CHOL:25
martinize2_path = martinize2
gmx_path = gmx_mpi
gpu = 0
gpcrdb_file_path = /path/to/gpcrdb/pdb/7fee_gpcrdb.pdb
bw_notation = True
output_dir = ./7fee
n_cores = 16
frame_timestep = 20
```

You can save the previous file as `7fee_analysis.prm` and execute the analysis.

```
$ CHAMP 7fee_analysis.prm
```

### (B) Using your own simulations

Same as in the previous situation, you might want to use your own trajectories, rather than simulating them again with CHAMP. In that case, the necessary considerations regarding length, timestep and writing frequency still stand valid and the `frame_timestep` parameter needs to be provided in case it does not match the expected 20 ps. As mentioned above, make sure to match the CHAMP directory structure and naming conventions for a smooth run. Once your inputs have been adapted, to perform the analyses on your trajectories simply run CHAMP as usual using the appropriate parameter file:

```
$ CHAMP 7fee_analysis.prm
```

## Input for pocket ranking (Optional)

As an optional step to be executed at the end of our protocol, we offer the possibility of ranking a list of geometrical pockets detected by external tools.

We tailored this tool to work with NanoShaper, but any other software that provides a list of residues contributing to the pocket can be used as long as the formatting of the pocket file complies with what CHAMP expects. For this example we used the web interface for NanoShaper and filtered the pocket list to avoid irrelevant results.

Feel free to used your preferred method, knowing that the format required is a basic CSV file with two columns as shown below, where the first one corresponds to the pocket ID (assigned by NanoShaper in our case) and the second to a list of residues contributing to each pocket (string of residue IDs, separated by white spaces). As an example, find below the pocket list we used in this tutorial, which we saved in a text file named `7fee_filtered_pockets.csv`:

```
6,133 136 137 140 394 395 397 398 404 405 408
7,292 295 296 299 300 303
9,138 141 142 148 153 154 157 158
11,165 169 172 190 191 192 194 195 198 199 241 245 248 249 252
12,283 284 287 288 291 352 353 356 357 360
14,291 294 295 346 349 350 353
15,201 204 205 208 209 240 243 244 247 279 282 283 286
```

The pocket file can be fed directly to a standard CHAMP run or to the corresponding standalone script by simply adding the following settings to the parameters file:

```
rank_pockets = True
pockets_file = /path_to_pockets_file/7fee_filtered_pockets.csv
```

Enabling this setting (and assuming a membrane composition of POPC(75%):CHOL(25%) was used) will produce the file `7fee_POPC_75_CHOL_75_ranked_pockets.csv` containing the pockets sorted by score, based on the propensity of cholesterol binding:

```
Pocket id,ResIDs,Contributing Points,Outlier Score,Total Score
11,165 169 172 190 191 192 194 195 198 199 241 245 248 249 252,1761,8000,9761
15,201 204 205 208 209 240 243 244 247 279 282 283 286,1634,6000,7634
12,283 284 287 288 291 352 353 356 357 360,1986,4000,5986
14,291 294 295 346 349 350 353,1232,3000,4232
9,138 141 142 148 153 154 157 158,287,1000,1287
7,292 295 296 299 300 303,473,0,473
6,133 136 137 140 394 395 397 398 404 405 408,349,0,349
```

## Cleaning up intermediate files

Considering the size of the data produced by a standard run of CHAMP (~750 GB), some housekeeping might be necessary in order to not overflow your disk space. Technically, while you can manually delete any file deemed as unnecessary

for your needs, CHAMP is shipped with a clean-up script ready to run once work is done and you are happy with the results.

Depending on how much data you intend to keep, the script can be run with one of the two options:

A) A full wipe command will remove all of the files of significant size (>1 GB) that can be reproduced again if re-running the analysis, efectively freeing up ~500 GB of space. To this end, you can run:

   $ CHAMP_tools cleanup full

B) Alternatively, a minimum wipe of the intermediate files `*_prod_sdf_1.xtc` can be performed for both replicas, freeing up ~250 GB and retaining the centered trajectory and the density cube file to inspect. Simply run:

   $ CHAMP_tools cleanup minimum

# Conclusive remarks

Finally, it is important to stress that, while changes and modifications of standard runs are possible, the results obtained by heavily altering the default parameters for which this tool was conceived might deviate substantially from what is expected. This is especially relevant regarding the length of the persistent contacts, as dramatically increasing the time between frames might lead to merging contacts that should otherwise be considered indepent events. The same can be said about shortening the simulation length, as the sampling of contacts might not be enough to distinguish relevant outliers.

# References

[1] Lomize, M. A.; Pogozheva, I. D.; Joo, H.; Mosberg, H. I.; Lomize, A. L. *OPM Database and PPM Web Server: Resources for Positioning of Proteins in Membranes.* **Nucleic Acids Research 2012**, *40* (D1), D370–D376. https://doi.org/10.1093/nar/gkr703

[2] Kooistra, A. J.; Mordalski, S.; Pándy-Szekeres, G.; Esguerra, M.; Mamyrbekov, A.; Munk, C.; Keserű, G. M.; Gloriam, D. E. *GPCRdb in 2021: Integrating GPCR Sequence, Structure and Function.* **Nucleic Acids Research 2021**, *49* (D1), D335–D343. https://doi.org/10.1093/nar/gkaa1080

[3] Eastman, P. *PDBFixer*; OpenMM Project. GitHub Repository. https://github.com/openmm/pdbfixer.

[4] Martini Force Field. *Frequently Asked Questions – How Large Can the Time Step Be?* https://cgmartini.nl/docs/faq/.
